# Pinene-Based Chiral Bipyridine Ligands Drive Potent Antibacterial Activity in Rhenium(I) Complexes

**DOI:** 10.3390/molecules30153183

**Published:** 2025-07-29

**Authors:** Justine Horner, Gozde Demirci, Aurelien Crochet, Aleksandar Pavic, Olimpia Mamula Steiner, Fabio Zobi

**Affiliations:** 1 Department of Chemistry, University of Fribourg, Chemin du Musée 9, CH-1700 Fribourg, Switzerland; justine.horner@hefr.ch (J.H.); goezde.demirci@unifr.ch (G.D.); aurelien.crochet@unifr.ch (A.C.); 2 Haute Ecole d’Ingénierie et d’Architecture Fribourg, HEIA-FR, HES-SO, Institute of Chemical Technology, University of Applied Sciences of Western Switzerland, Pérolles 80, CH-1705 Fribourg, Switzerland; 3 Institute of Molecular Genetics and Genetic Engineering, University of Belgrade, Vojvode Stepe 444a, 11042 Belgrade, Serbia; sasapavic@imgge.bg.ac.rs

**Keywords:** rhenium, tricarbonyl, chiral, pinene, antimicrobial

## Abstract

Antimicrobial resistance (AMR) poses a critical global health threat by rendering existing antibiotics ineffective against infections, leading to increased mortality, prolonged illnesses, and higher healthcare costs. Developing new antibiotics is essential to combat resistant pathogens, safeguard modern medical procedures, and prevent a return to a pre-antibiotic era where common infections become untreatable. We report a series of chiral tricarbonyl rhenium(I) complexes incorporating enantiopure pinene-substituted bipyridine ligands (L#) of the general formula *fac*-[Re(CO)_3_L#X] and *fac*-[Re(CO)_3_L#Py]^+^ (where X = Cl or Br and Py = pyridine). These complexes were isolated as mixtures of two diastereomers, characterized by standard techniques, and evaluated for cytotoxic activity against methicillin-resistant and methicillin-sensitive *Staphylococcus aureus* (MRSA and MSSA). The results revealed notable antibacterial efficacy (MIC = 1.6 μM), reflected in high therapeutic indices (Ti > 10). In contrast, analogous complexes bearing non-chiral 2,2′-bipyridine ligands exhibited no activity, underscoring the critical role of chirality in modulating biological interactions at the molecular level. These findings highlight the potential of chiral Re(I) complexes as promising scaffolds for the development of more potent and selective antibacterial agents.

## 1. Introduction

Bioactive rhenium complexes bearing chiral ligands have garnered significant interest in medicinal chemistry. These complexes can be synthesized using a variety of chiral ligands, such as alcoholates, amidates, and iminopyridines, resulting in diastereomeric mixtures [1]. For instance, the synthesis of tetracarbonyl (pyrrolylimine) rhenium complexes with chiral pyrrolyl ligands has been reported, demonstrating potential as CO-releasing molecules [2]. Chiral rhenium thiolate complexes have been obtained using functionalized thiolate ligands, including amino acid derivatives such as cysteine and proline [3]. The chirality of both the metal center and the ligands has a pronounced impact on the biological activity of these compounds, as shown in cytotoxicity studies against various cancer cell lines, particularly glioblastoma [1]. These findings highlight the potential of chiral rhenium complexes in bioorganometallic chemistry and their applications in medicinal research.

Several studies have already highlighted the significant potential of rhenium complexes as anticancer and/or antimicrobial agents [4,5,6,7,8,9,10,11,12,13,14]. While the mechanisms underlying their bioactivity are diverse and not yet fully understood, they are believed to involve interactions with key biomolecules such as proteins or DNA [5,9,14,15]. These interactions are modulated by the stereochemistry of the complexes, as demonstrated in numerous studies [16,17,18,19].

**Figure 1:**
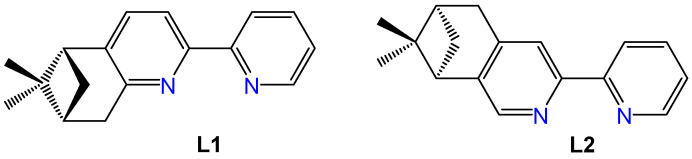
Structures of the ligands (−)-5,6- pinene bipyridine (**L1**) and (−)-4,5-pinene bipyridine (**L2**).

Enantiopure pinene derivatives of bi, tri, or tetrapyridine ligands have been widely studied for their ability to predetermine the chirality in coordination compounds possessing d or f metal centers [20,21]. Among these, a manganese complex incorporating the (−)-5,6-pinene bipyridine ligand (**L1**, Figure 1) has shown notable anticancer activity and strong selectivity toward tumor cells [22]. The first Re(I) complexes featuring pinene-polypyridine ligands were reported by Yeung et al. in 2009 employing 4,5 or 5,6 pinene-functionalized quaterpyridine ligands to generate dinuclear, monostranded helical structures [23]. Later, in 2013, Zheng et al. synthesized the complex [Re(CO)_3_**L2**Cl] (see Figure 1 for the structure of (−)-4,5-pinene bipyridine, **L2**) and investigated its ferroelectric properties [24]. However, none of these early complexes were evaluated for potential biomedical applications.

Recently, our groups initiated a systematic study of Re(I) complexes bearing pinene-based (poly)pyridine ligands. We explored the anticancer properties of dinuclear tricarbonyl Re(I) complexes incorporating bis-bipyridine ligands functionalized with pinene units, highlighting the crucial role of chirality in mediating cytotoxicity effects against HCT-116 and MCF-7 cancer cell lines [25]. We also examined the antimicrobial activity of clotrimazole tricarbonyl Re(I) complexes containing **L1**, **L2**, or bis-pinene bipyridine derivatives [26]. These compounds, alongside achiral analogs, were tested against methicillin-sensitive and -resistant *S. aureus* strains. The results were part of a computer-aided drug design (CADD) model using a de novo ‘scaffold-hopping’ strategy for antibacterial evaluation. This approach identified chiral **L1** and **L2** pinene bipyridine derivatives as the most promising candidates.

Building on these insights, here, we present the synthesis, characterization, and antimicrobial evaluation of a series of Re(I) tricarbonyl complexes, containing a single bidentate ligand (**L1** or **L2**, Figure 1). We studied four neutral complexes, each bearing a coordinated halide ligand (Cl or Br). In addition, four cationic complexes (+1 charge) were synthesized in which the halide is substituted by pyridine. These species were isolated as hexafluorophosphate or triflate salts. It should be noted here that the choice of preparing two different salts of the cationic complexes was dictated by the fact that anionic counter-ions significantly influence the antibiotic activity of cationic metal complexes by affecting solubility, dissociation, and adsorption onto bacterial cell surfaces [27]. Studies indicate that the choice of counter-ion can enhance or diminish antimicrobial efficacy, with smaller atomic radii potentially allowing better separation from the cation, thus improving activity [28,29,30,31,32]. However, no consistent trends have yet been established, as factors like atomic radii, charge density, and solubility all contribute to the complex interactions affecting antimicrobial effectiveness.

The results obtained from their antibiotic evaluation in vitro point to a remarkable increase in the antimicrobial activity (up to ~60-fold improvement in the activity) when compared to closely related achiral complexes. In addition, we present mode of action studies which revealed not only that neutral and cationic complexes have different functional mechanisms but that the chiral nature of the ligand remarkably influences the antimicrobial efficacy of the compounds in the structurally related series evaluated.

## 2. Results and Discussion

### 2.1. Synthesis and Characterization of Re(I) Tricarbonyl Complexes

**Figure 2:**
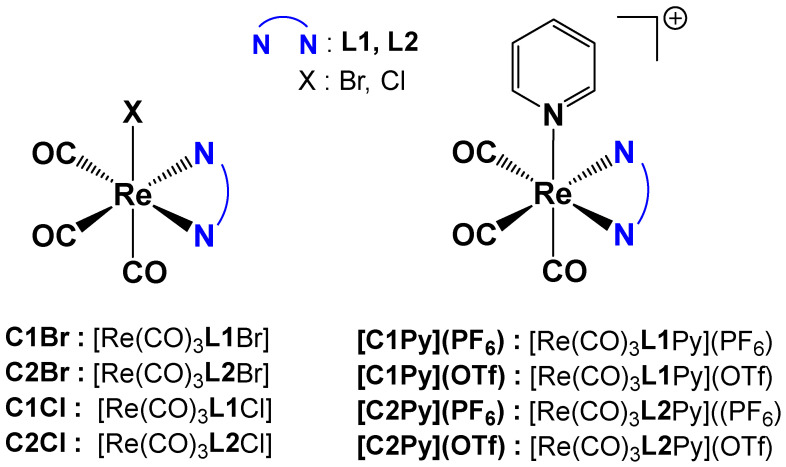
General formula of the Re(I) tricarbonyl complexes and their assigned names.

Re(I) tricarbonyl complexes were prepared following a modified procedure from the literature [6]. A total of eight different complexes (Figure 2) were synthesized. Complexes **C2Cl**, **C1Cl**, **[C2Py](PF_6_)** and **[C1Py](PF_6_)** were prepared starting from the commercially available [Re(CO)_5_Cl] (Figure 2). All the other tricarbonyl complexes were prepared from [Re(CO)_5_Br], which is easily synthesized by the oxidation of commercial dirhenium decacarbonyl with bromine [33].

Neutral complexes **C1Br**, **C2Br**, **C2Cl**, and **C1Cl** were obtained in a one-step reaction by overnight heating of [Re(CO)_5_X] with one equivalent of the pinene bipyridine ligand in toluene (Figure 2). After workup, the yellow Re(I) complexes were obtained in good yields (60–80%) and high purity. The cationic complexes with triflate counterions, **[C2Py](OTf)** and **[C1Py](OTf)**, were obtained from previously synthesized bromo-derivatives of **C1Br** or **C2Br** complexes, with the bromo-ligand being substituted by pyridine in the presence of AgOTf (Figure 2). The reaction conditions were inspired by our previous work [5]. The cationic complexes possessing hexafluorophosphate counterions **[C2Py](PF_6_)** and **[C1Py](PF_6_)** were obtained by substitution of the triflate counterions with hexafluorophosphate in the presence of NH_4_PF_6_ (Figure 2). After careful elimination of the silver salts, pure **[C2Py](PF_6_)** (55% yield) and **[C1Py](PF_6_)** (67% yield) were obtained. Each complex (as a diastereoisomeric mixture) was characterized by ^1^H NMR spectroscopy, mass spectrometry, and FTIR spectroscopy. In addition, several complexes formed single crystals, and their structure was determined by X-ray diffraction (vide infra). Neutral complexes are soluble in organic solvents like DMSO, CHCl_3_, or CH_3_CN but not in water. In a 2:8 DMSO:water mixture, saturation is reached at ca. 0.1 mM. Cationic complexes show a similar behavior, with **[C2Py](OTf)** and **[C1Py](OTf)** being the most hydrophilic species.

With a coordination number of 6, *fac*-Re^I^(CO)_3_ complexes possess three other coordinating sites in addition to the three carbonyls, respecting a classical octahedral geometry. The three carbonyl ligands could be considered as a unit, and the geometry of the complex will therefore be comparable to a pseudo-tetrahedral geometry, inevitably creating a stereogenic Re(I) metal center as long as the other three monodentate ligands are different. If a non-chiral bidentate ligand is used, i.e., 2,2′-bipyridine, the metal center is not chiral. But if the bidentate ligand is chiral, as are the two enantiopure ligands **L1** and **L2**, the metal center can have two configurations. In this case, two diastereoisomers can be obtained (same chirality of the ligand but opposite configurations of the metal center).

**Figure 3:**
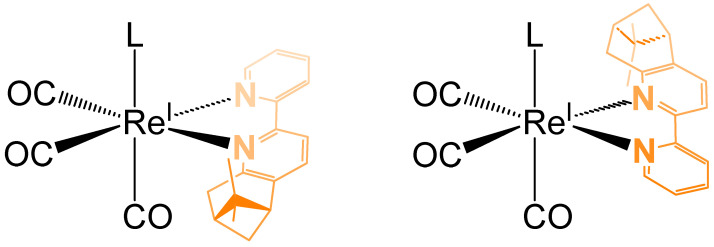
The two diastereomeric forms of the chiral complex **C1Br** (left: *cis*, right: *trans*).

In Figure 3, the two diastereomeric forms of the complex **C1Br** are represented. Taking in consideration the relative position of the two methyl groups from the pinene moiety in relation with the monodentate ligand (in our case Br, Cl or pyridine), the *cis* diastereoisomer is the one in which the axial ligand (L) is located in the same hemisphere as the two methyl groups of pinene (Figure 3, left). If this is not the case (Figure 3, right), then the distereoisomer possesses a *trans* configuration.

The ^1^H-NMR spectra indeed demonstrate that the **L1** and **L2** complexation led to equimolar mixtures of the two diastereomers (Figures S1 and S2). As expected, the position of the protons signals of the ligand are shifted when coordinated to the Re(I) center, and some of the signals are clearly split, denoting the presence of the two diastereoisomers. Instead of two singlet signals for the two diastereotopic methyl groups present in the free ligand, four singlets of equal intensity are present in the Re(I) complexes. The other very affected protons are the diastereotopic ones in the alpha-position to the bipyridine. The replacement of the halide ligand in the coordination sphere by pyridine results in more complex spectra. This is due to the presence of additional signals from the pyridine protons in the aromatic region, as well as their interactions with protons from the chiral ligands. (Figures S3 and S4). Attempts to separate the diastereoisomers of the neutral complexes by achiral chromatography led to enriched fractions of one or another diastereoisomer (Figure S5) but never to pure compounds. In the case of cationic complexes, the tedious procedures to eliminate any trace of silver cations that would have compromised the antimicrobial test results led to chemically pure complexes. The high resolution and the ESI-MS spectra of all complexes show the presence of the molecular peak [M-Na]^+^ for the neutral complexes or [M]^+^ for the cationic ones with the characteristic isotopic pattern for Re species (see Figures S6–S13 in Supplementary Matherial). Some additional signals were visible, corresponding to complexes having lost their halide: [M-X]^+^ or complexes having substituted their halide by the solvent (methanol): [M-X+MeOH]^+^. Finally, the IR spectra of the complexes show the typical tricarbonyl stretching vibrational pattern of the *fac*-[Re(CO)_3_]^+^ core in the 2010–2030 and 1900 cm^−1^ region for the symmetric and asymmetric stretching modes, respectively. These bands are known to be very sensitive to the electronic environment and oxidation state of the metal ion [34,35,36], in addition to the ligand system. If one considers the frequencies of the symmetric stretching modes of the complexes, it is apparent that **L1** and **L2** have similar electronic effects to bipyridine [37,38]. In addition, the IR spectra of **[C1Py](PF_6_)** and **[C2Py](PF_6_)** show peaks in the 830–850 and 555–560 cm^−1^ region attributed, respectively, to the asymmetric stretching vibrations of the P-F bonds and the bending vibrations within the PF_6_^−^ ion.

### 2.2. Single-Crystal X-Ray Diffraction

**Figure 4:**
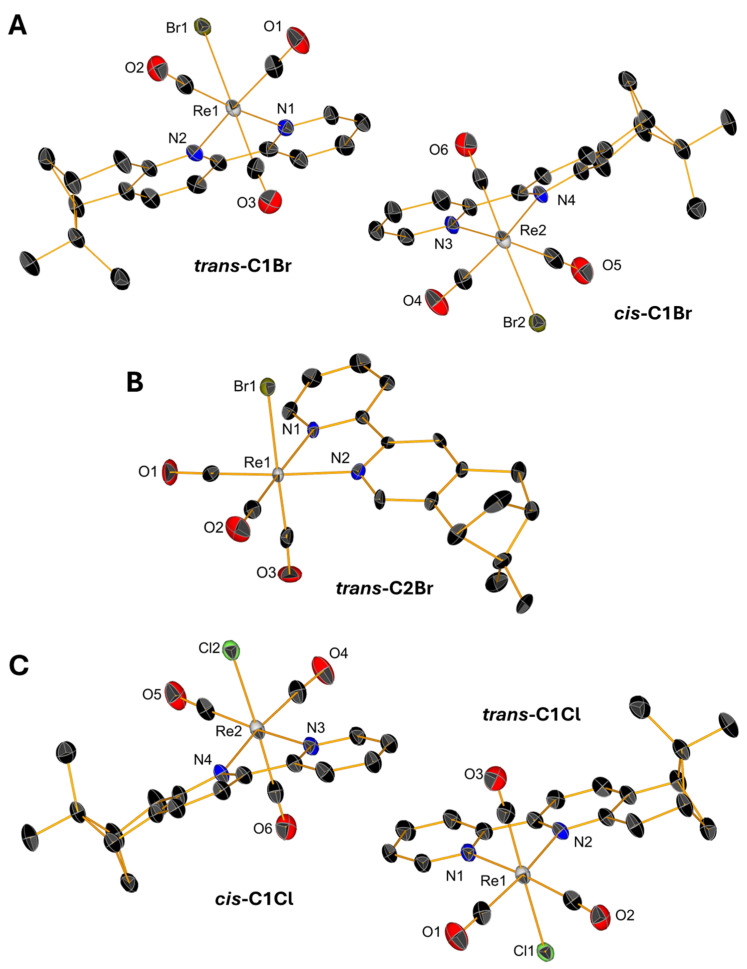
Single-crystal X-ray diffraction structures of **C1Br** (**A**), **C2Br** (**B**), and **C1Cl** (**C**) depicting the coordination sphere of the pair of diastereoisomers present in the asymmetric unit. Thermal ellipsoids are at 30% probability. Hydrogen atoms are omitted for clarity.

**Figure 5:**
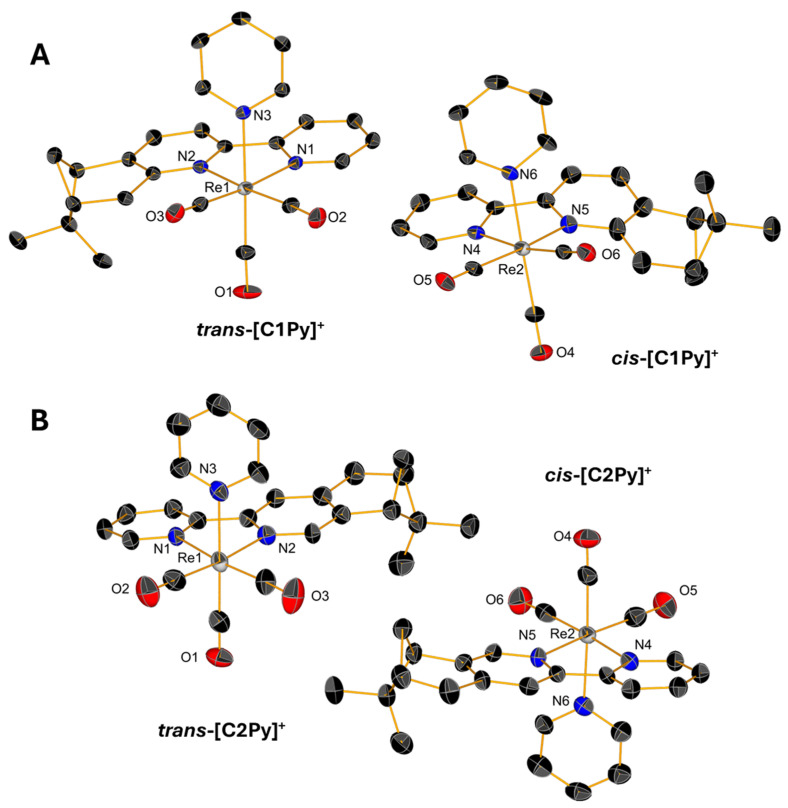
Single-crystal X-ray diffraction structures of cations of **[C1Py](OTf)** (**A**) and **[C2Py](OTf)** (**B**) depicting the coordination sphere of the pair of diastereoisomers present in the asymmetric unit. Thermal ellipsoids are at 30% probability. Hydrogen atoms are omitted for clarity.

Slow evaporations of the CDCl_3_ solutions of **C1Br**, **C1Cl**, and **C2Br** in NMR tubes lead to the formation of yellow, single crystals whose structures were determined by X-ray diffraction. Single crystals of triflate complexes **[C1Py](OTf)** and **[C2Py](OTf)** were obtained from concentrated solutions of methanol containing water. All the complexes crystallize in the P2_1_ space group with the exception of **[C1Py](OTf)**, which crystallizes in the P2_1_2_1_2_1_ space group (Table S1 in Supplementary Material). The crystals contain two molecules in the asymmetric unit (Figure 4 and Figure 5), one corresponding to the diastereoisomer *cis* and the other to the diastereoisomer *trans* (vide supra). Only the crystal measured from a sample of **C2Br** containing equimolar amounts of both diastereoisomers showed only one molecule in the unit cell, which is the *trans* diastereoisomer (Figure 4B). In all the complexes, the Re(I) center adopts, as expected, a pseudo-octahedral geometry with a *fac* configuration. The lengths of the coordination bonds are all in the expected range, the longest ones being those between the halogens and the Re(I) with values between 2.773 (6) and 2.5730 (12) (see Table S2) [21]. As observed in the dinuclear complexes reported by Yeung et al. [23] and Solea et al. [25], intermolecular C-H∙∙∙X (Cl, Br) electrostatic interactions involving aromatic Hs are present in the crystalline structures of **C1Cl**, **C2Cl**, and **C2Br**. The measured values for these intermolecular distances are between 2.865 (1) and 2.984 (1) Å in **C1Br**, 2.765 (1)–2.901 (2) Å in **C1Cl**, and 3.057 (1)–3.124 (1) Å in **C2Br** (Figures S13–S15).

### 2.3. Antimicrobial Evaluation

The antimicrobial activity of the rhenium tricarbonyl mononuclear complexes and their corresponding pinene-bipyridine ligands was determined against two *S. aureus* strain: methicillin-resistant *S. aureus* (MRSA 43300) and methicillin-sensitive *S. aureus* (MSSA 25923). These microorganisms are responsible for most nosocomial infections [39]. The results are summarized in Table 1. Minimum inhibitory concentration (MIC) values ≤ 3.13 μM are an indication of good antimicrobial activity. Cationic rhenium tricarbonyl complexes were expected to be the most active of the series, as observed in previous studies [6]. Their mechanism of action is not yet fully understood, but we hypothesized that their positive charge allows them to interact more easily with biomolecules than neutral complexes, and/or that they more strongly interact with the negatively charged cellular membrane [40,41]. Indeed, the four cationic complexes tested (**[C1Py](OTf)**, **[C2Py](OTf)**, **[C1Py](PF_6_)**, and **[C1Py](PF_6_)**) were very active against both MRSA and MSSA strains, with a remarkable MIC value of 1.6 μM.

Unexpectedly, the two neutral complexes, **C2Br** and **C2Cl**, also demonstrated very good activity against *S. aureus*, with MIC values of 6.25 μM. It seems that the structure of the bidentate diimine ligand plays an important role, since the neutral complexes [6] and **C1Br** and **C1Cl** featuring the 5,6-pinbpy ligand are inactive. A comparison between neutral and cationic complexes is risky, since their structure–activity relationship could be very different, as well as their mode of action. In fact, a look in the literature revealed that in other contexts, it has already been observed that neutral rhenium tricarbonyl complexes can also be active, as an anti-COVID agent [42] or as an anticancer agent [43]. Both Cohen and Amezquita Valencia argued that the activity of neutral compounds can be correlated with the lability of the monodentate ligand in ligand exchange mechanisms [42,43]. Once in a biological condition, the halide ligand would dissociate and the remaining active species [Re(CO)_3_**L2**]^+^ would then be able to coordinate to biomolecules’ heteroatoms, particularly to specific amino-acid residues.

To make a simple evaluation of the potential of using chiral ligands for bio applications, it is interesting to compare the results obtained here to those of corresponding complexes containing achiral simple bipyridine as a bidentate ligand. The MIC value against MRSA obtained for **C2Br** was 6.25 μM. In comparison, the MIC value of the non-chiral [Re(CO)_3_(bpy)Br] is >50 μM, which is ca. a 10-fold higher score for the same core structure. Similarly, by comparing the activity of the cationic complexes **[C1Py]^+^** and **[C2Py]^+^** to that of the achiral [Re(CO)_3_(bpy)(Py)]^+^ species (MIC = 100 μM), a remarkable 62-fold improvement in the antibiotic activity of the complexes was observed. This observation highlights the potential of chiral ligands and the need for further in-depth study to understand how their use may allow an order of magnitude increase in the complexes’ activity.

Furthermore, it is worth mentioning that free 4,5-pinbpy and 5,6-pinbpy ligands were both active against the microorganisms tested (MIC = 3.13 μM). Unfortunately, their high toxicity toward healthy eukaryotic cells (IC_50_ of ca. 2.5–5 µM for both ligands) rendered them unsuitable as potential candidates alone. In contrast, when coordinated to a rhenium tricarbonyl core, the resulting complexes were much less toxic (IC_50_ of complexes between 18 and 24 µM). A similar observation was already made by Sovari et al. in 2021 [6]. The binding of bioactive organic molecules to metal complexes can modify their biological properties. The reasons are multiple (different mechanisms of action, different cellular uptake, different interactions with biomolecules). This is one of the strategies currently used for the improvement in anticancer and infection treatments [44,45].

### 2.4. Effects of Complexes on the Functionality and Integrity of the Cytoplasmic Membrane

**Figure 6:**
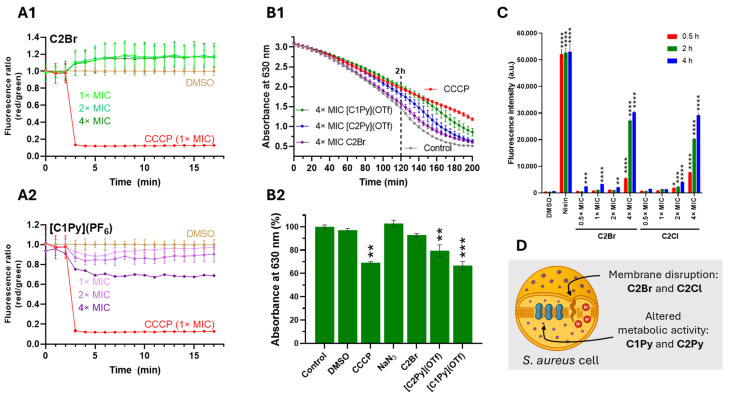
Complexes do not induce membrane depolarization in *S. aureus* MRSA. Complexes **[C1Py]^+^** and **[C2Py]^+^** reduce the reductive capacity of the bacteria, while **C2Br** and **C2Cl** facilitate propidium iodide uptake by the bacterium. (**A**). Examples of the time-dependent effects of **C2Br** and **[C1Py](PF_6_)** (**A1** and **A2**, respectively) on the membrane potential of *S. aureus* cells. Membrane potential was measured as a function of the red to green fluorescence ratio of [DiOC_2_(3)]. Protonophore CCCP (12.5 μM, 1× MIC) was used as a positive control and DMSO as a negative control. Data represent the mean of three biological replicates, with error bars indicating the standard deviation of the mean (see Supplementary Material for all datasets). (**B**). Time-dependent activity of selected complexes at 4× MIC on the respiratory chain of *S. aureus* MRSA cells (**B1**) and relative effect of the complexes at time 120 min (**B2**). The metabolic activity of *S. aureus* was measured by the reduction of resazurin to resorufin. CCCP and NaN_3_ were used as positive and negative controls. CCCP uncouples the respiratory chain from the proton gradient, while NaN_3_ is an inhibitor of cytochrome c oxidase (absent in *S. aureus*). Data represent the mean of three biological replicates, with error bars in B2 indicating the standard deviation of the mean. Statistical significance: (**) *p* < 0.02, (***) *p* < 0.0002. (**C**). PI fluorescent intensity of *S. aureus* cells treated with DMSO (1%), Nisin (100 µg/mL), and 4 different concentrations of complexes **C2Br** and **C2Cl** at three different time points. Data represent the mean of three biological replicates, with error bars indicating the standard deviation of the mean (see Supplementary Material for all datasets). Statistical analysis was performed against DMSO-treated control cells. Statistical significance: (*) *p* < 0.03, (**) *p* < 0.02, (***) *p* < 0.0002, (****) *p* < 0.0001. (**D**). Cartoon representation of a single *S. aureus* cell with the main effects of active complexes.

Having identified highly active complexes, we decided to evaluate how the compounds affect the functionality and integrity of the cytoplasmic bacterial membrane. Data on the mechanism of action of antibiotic rhenium complexes are scarce, but effective compounds are reported to either inhibit membrane-associated stages of peptidoglycan (PG) synthesis [15] or affect the cytoplasmic membrane by disrupting its architecture [9,46]. First, we inspected the effects on the membrane potential using the fluorescent probe 3,3′-diethyloxacarbocyanine iodide [DiOC_2_(3)]. Under normal conditions, with optimal membrane potential, DiOC_2_(3) emits green fluorescence, but when the bacterial membrane potential is disrupted, the dye aggregates and shifts to red fluorescence [47]. Changes in membrane potential were monitored by calculating the red to green fluorescence ratio in response to the different complexes, using the protonophore CCCP as a positive control [48]. Our results indicate that none of the molecules significantly altered the membrane potential of *S. aureus* MRSA bacteria (Figure 6A and Figure S21).

Since membrane depolarization may stem either from direct membrane disruption or from interference with the electron transport chain [49], we evaluated the cells’ reducing capacity by monitoring the conversion rate of resazurin to resorufin [50,51]. The incubation of *S. aureus* MRSA cells with the complexes did not significantly reduce the reductive capacity of the bacteria (Figure S22). Only **[C1Py](OTf)** at 4× MIC showed an effect comparable to that of the electron transport chain decoupler CCCP, after 2 h to 3 h of incubation (Figure 6B). Next, we examined the integrity of the cytoplasmic membrane by incubating *S. aureus* MRSA cells with the complexes along with the non-permeant dye propidium iodide (PI) in a concentration- and time-dependent assay. PI, which cannot cross intact cell membranes, enters only when the membrane is compromised; once inside, it intercalates with DNA to emit red fluorescence [52]. Of all the active molecules tested, only **C2Br** and **C2Cl** facilitated PI uptake at a 4× MIC concentration (Figure 6C and Figure S23), showing comparable results to the positive control nisin [52].

### 2.5. In Silico Molecular Analysis

Having observed effects on the reductive capacity (or metabolic activity) of the bacteria by **[C1Py]^+^** and **[C2Py]^+^**, we computationally evaluated the interaction of the complexes with the bacterial oxidases. *Staphylococcus aureus* employs two terminal oxidases, *qoxABCD* and *cydAB*, which are critical for its aerobic respiration and play significant roles in bacterial fitness and pathogenicity during infections [53,54]. While the structural details of these oxidases remain uncharacterized in *S. aureus*, homologs from *E. coli* [55] and *Geobacillus* [56] species can be utilized as structural templates (e.g., AlphaFold) to model the docking interactions of compounds. Our in silico analysis aimed to assess whether the complexes could selectively bind to ubiquinol interaction sites (*qoxABCD*) or oxygen entry channels, potentially disrupting the respiratory chain. We independently evaluated the diastereoisomers of all complexes, including inactive species (e.g., **C1Br** and **C2Br**), to check if these gave similar results to the active compounds. In all cases, interaction of the complexes with *cydAB* revealed the identical non-specific binding site of the enzyme in the cytoplasmic side of the polypeptide (Figure S24), excluding the possibility that active compounds may act by interfering with the action of this oxidase. With reference to *qoxABCD*, we found similar results, except for the *cis* diastereomers of **[C1Py]^+^** and **[C2Py]^+^**. Indeed, docking simulations with these diastereomers revealed that they could localize near the ubiquinol-binding pocket in the *E. coli qoxABCD* model (Figure S24), suggesting a potential site-specific interaction. Whether or not this interaction may play a role in the mode of action of the complexes, the results again underscore the critical role of chirality in modulating biological interactions at the molecular level.

**Figure 7:**
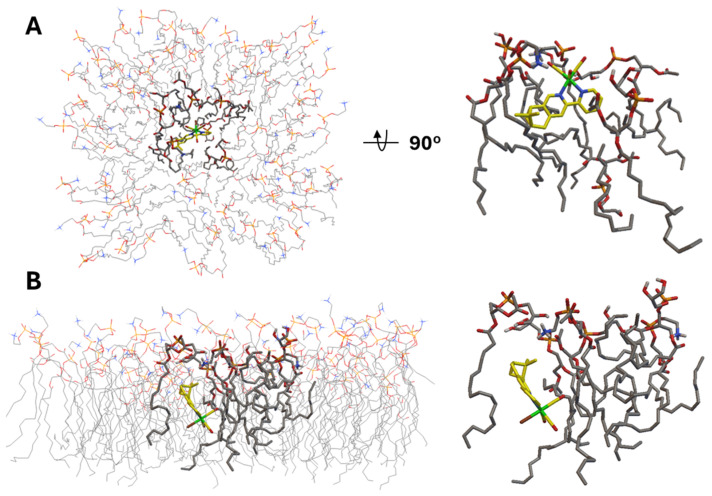
(**A**). Top and side views of the lowest-energy insertion conformation (−10.3 kcal/mol) of **C2Br** in the semi-flexible POPG:POPE membrane model. (**B**). Side views of the lowest-energy embedded conformation (−13.2 kcal/mol) of **C2Br** in the same membrane model.

Next, we moved to the interaction analysis of **C2Br** and **C2Cl** with the bacterial cytoplasmic membrane. We mentioned above that the two complexes uniquely disrupt the cytoplasmic membrane integrity. Therefore, we analyzed the in silico interaction of **C2Br** with a semi-flexible POPG:POPE membrane model constructed on the basis of what was reported by Jämbeck and Lyubartsev (Figure 7) [57]. The results of this analysis revealed two major interactions of **C2Br** with the membrane model, which we refer to as insertion (Figure 7A, −10.3 kcal/mol) and embedding (Figure 7B, −13.2 kcal/mol). Insertion of **C2Br** in the lipid layer occurs via the **L2** ligand, with the structure being stabilized by weak, electrostatic H-bonding interactions between the lipids’ amino and glycero head groups and the Re-bound Br and CO ligands (2.7 and 3.5–4 Å, respectively, Figure S25). In addition to insertion, a lower-energy model interaction reflects the embedding of the complex in the lipid layer (Figure 7B). In this conformation, disruption of lipid compactness in favor of a pocket housing the complex is evident in Figure 7B and may help to explain the experimental data collected in the PI assay mentioned in the previous section.

## 3. Materials and Methods

Reagent grade chemicals were purchased from Sigma Aldrich (Darmstadt, Germany) and Acros Organics (Geel, Belgium) and used without further purification. The ligands L1 and L2 were synthesized following the literature [58]. [Re(CO)_5_Br] was obtained from the commercially available dirhenium decacarbonyl and bromine using a reported procedure [33]. Yields reported are for isolated, spectroscopically pure compounds. NMR spectra were recorded on a Bruker Advance DPX 300 spectrometer (Bruker Corporation, Billerica, MA, USA) using TMS or the residual solvent proton as an internal standard. Mass spectra were obtained using a Bruker Esquire HCT mass spectrometer fitted with an electro-spray ion (ESI) source. The experiments were performed with spray solvent methanol, injected with a micro-syringe connected to an automatic pump. HRMS spectra were recorded on a Bruker FTMS 4.7T BioAPEX II and Waters SynaptG2-Si (Waters Corp., Milford, MA, USA) or at the Mass Services of the Universities of Bern and Zurich. IR spectra were recorded on a Bruker TENSOR II spectrometer, and samples were measured with OPUS using a Golden Gate. The parameters were the same for all samples, 16 scans for backgrounds and 32 scans for samples, with a resolution of 4 cm^−1^ in the 4000–600 cm^−1^ region. Single crystals were selected and mounted on a loop with oil, measured on a Stoe StadiVari or Stoe IPDS2 diffractometer (STOE & Cie GmbH, Darmstadt, Germany). Crystals were kept at 250 (2) K during data collection. Using Olex2 [59], the structures were solved with the SHELXT [60] structure solution program using Intrinsic Phasing and refined with the SHELXL [61] refinement package using Least Squares minimization. Antimicrobial MIC determination was performed as previously described [62]. Time-dependent membrane potential measurement was performed according to the reported procedure using the *S. aureus* MecA R2954 (MRSA) strain [15]. Cell cytotoxicity measurements were performed according to Rahmani et al. [63].

### 3.1. Synthesis of C1X and C2X (Where X = Br, Cl)

A mixture of the ligand (L1 for C1X or L2 for C2X) (62 mg, 0.25 mmol, 1 eq) and Re(CO)_5_Br or Re(CO)_5_Cl (for X = Br and Cl, respectively, 102 mg (Br) 92 mg (Cl), 0.25 mmol, 1 eq) were dissolved under inert atmosphere in THF (4 mL, HPLC grade). The reaction mixture was refluxed under an inert atmosphere for 16 h at 85 °C and afterward left to cool to RT and then cooled to 4 °C under gentle stirring. A yellow precipitate formed rapidly and was recovered by filtration after 2 h of stirring at 4 °C, washed with cold THF (3 × 2 mL), and finally dried under high vacuum to give a yellow solid.

Crystals of **C1Br** (as a stereoisomer mixture) were grown by layering pentane on the compound in CDCl_3_ solution in an NMR tube. Crystals of **C2Br** (as a stereoisomer mixture) were grown by layering hexane on the compound in CDCl_3_ solution in an NMR tube. Crystals of **C2Cl** (as a stereoisomer mixture) were grown by layering hexane on the compound in CDCl_3_ solution in an NMR tube. The crystals of the complexes were obtained in 10–14 days as yellow needles.

**C1Br**. Yield: 98 mg (66%). IR (cm^−1^): 2011s (νCO, sym), 1880w (νCO, asym). ESI-MS (MeOH): *m*/*z*, 622.6 [Re(CO)_3_L1Br + Na]^+^. ^1^H NMR (300 MHz, CDCl_3_) δ 9.15–9.07 (m, 1H, H1), 8.10 (dd, J = 8.3 Hz, 1H, H4), 8.04–7.95 (m, 1H, H7), 7.92 (d, J = 8.0 Hz, 1H, H3), 7.53 (dd, J = 8.0 Hz, 1H, H8), 7.50–7.42 (m, 1H, H2), 3.74–3.40 (m, 2H, H13), 2.99–2.88 (m, 1H, H10), 2.83–2.69 (m, 1H, H15b), 2.62–2.50 (m, 1H, H12), 1.46 and 1.45 (s, J = 3.8 Hz, 3H, H17), 1.37 (dd, J = 17.7 Hz, 1H, H15a), 0.74 and 0.72 (s, 3H, H16).

**C2Br**. Yield: 117 mg (79%). IR (cm^−1^): 2015s (νCO, sym), 1911s (νCO, asym), 1883s (νCO, asym). ESI-MS (MeOH): *m*/*z*, 622.6 [Re(CO)_3_L2)Br + Na]^+^. ^1^H NMR (300 MHz, CDCl_3_) δ 9.06 (dd, J = 5.5 Hz, 1H, H1), 8.58 (d, J = 1.1 Hz, 1H, H8), 8.13 (d, J = 8.1 Hz, 1H, H4), 8.02 (ddd, J = 8.2 Hz, 1H, H3), 7.93 (s, 1H, H7), 7.48 (ddd, J = 7.5 Hz, 1H, H2), 3.15 (d, J = 2.8 Hz, 2H, H13), 3.00 (td, J = 5.4 Hz, 1H), H10, 2.89–2.73 (m, 1H, H15b), 2.40 (s, 1H, H12), 1.47 and 1.46 (s, J = 1.6 Hz, 3H, H17), 1.28 (dd, J = 10.1 Hz, 1H, H15a), 0.73 and 0.72 (s, 3H, H16).

**C1Cl**. Yield: 101 mg (72%). IR (cm^−1^): 2012s (νCO, sym), 1906s (νCO, asym), 1889s (νCO, asym), 1875s (νCO, asym). ESI-MS (MeOH): *m*/*z*, 578.7 [Re(CO)_3_L1)Cl + Na]^+^. ^1^H NMR (300 MHz, CDCl_3_) δ 9.09 (m, J = 5.5 Hz, 1H, H1), 8.09 (m, J = 8.3 Hz, 1H, H4), 8.05–7.94 (m, 1H, H7), 7.91 (d, J = 8.0 Hz, 1H, H3), 7.54 (d, J = 8.0 Hz, 1H, H8), 7.51–7.40 (m, 1H, H2), 3.75–3.40 (m, 2H, H13), 2.94 (t, J = 5.7 Hz, 1H, H10), 2.83–2.69 (m, 1H, H15b), 2.60–2.52 (m, 1H, H12), 1.45 and 1.46 (s, J = 3.1 Hz, 3H, H17), 1.37 (dd, J = 22.2 Hz, 1H, H15a), 0.73 and 0.71 (s, 3H, H16).

**C2Cl**. Yield: 100 mg (72%). IR (cm^−1^): 2018s (νCO, sym), 1911s (νCO, asym), 1885w (νCO, asym). ESI-MS (MeOH): *m*/*z*, 578.7 [Re(CO)_3_L2Cl + Na]^+^. ^1^H NMR (300 MHz, CDCl_3_) δ 9.04 (m, J = 5.5 Hz, 1H, H1), 8.57 (dd, J = 1.5 Hz, 1H, H8), 8.16–8.10 (m, 1H, H4), 8.07–7.99 (m, 1H, H3), 7.93 (s, 1H, H7), 7.54–7.43 (m, 1H, H2), 3.18–3.10 (m, 2H, H13), 3.04–2.94 (m, 1H, H10), 2.89–2.73 (m, 1H, H15b), 2.45–2.37 (m, 1H, H12), 1.47 and1.46 (s, J = 1.8 Hz, 3H, H17), 1.27 (dd, 1H, H15a), 0.73 and 0.71 (s, 3H, H16).

### 3.2. Synthesis of Cationic Complexes with Triflate Counterions: **[C1Py](OTf)**, **[C2Py](OTf)**

The previously prepared complexes (**C1Br** or **C2Br**) (62 mg, 0.1 mmol, 1 eq) were dissolved in anhydrous MeOH (6 mL). Pyridine (18 mg, 18.4 µL, 0.2 mmol, 2 eq) dissolved in MeOH (1 mL) was added, and the flask was covered with aluminum foil to protect the mixture from light. AgOTf (44 mg, 0.2 mmol, 1.6 eq) in MeOH (1 mL) was added. The reaction mixture was stirred at 65 °C under an inert atmosphere in the absence of light for 16h and then allowed to cool down at RT. The white precipitate formed, AgBr, was removed by filtration from the yellow solution. The filtrate was collected, the solvent eliminated under reduced pressure, and the solid was dried under high vacuum. No yield was calculated, as crystallization began after dissolving the solid in MeOH/H_2_O (for preparative HPLC injections). Only the supernatant was injected for HPLC purification, while the crystals that formed at the bottom of the vial were collected and dried. The same was used for X-ray measurements. The collected supernatant was purified by C18 reverse phase prep HPLC (Column: Macherey-Nagel VP 250/21 NUCLEODUR C18 HTec, 5 μm) (Macherey-Nagel, Düren, Germany) in order to eliminate the traces of silver inorganic salts (mainly AgBr), resulting from the reaction. The analyses and the biological tests were performed on both the collected crystals and the HPLC-purified product. No differences were observed in the results. Single crystals of triflate complexes **[C1Py](OTf)** and **[C2Py](OTf)** were obtained from concentrated solutions of methanol containing water in a 1:1 volume ratio.

**[C1Py](OTf)**. Yield: 50.1 mg (64%). IR (cm^−1^): 2028s (νCO, sym), 1902w (νCO, asym). ESI-MS (MeOH): *m*/*z*, 599.7 [Re(CO)_3_L1Py]^+^. ^1^H NMR (300 MHz, CD_3_CN) δ 9.22–9.14 (m, 1H, H1), 8.34–8.06 (m, 5H), 7.93–7.82 (m, 1H), 7.80–7.75 (m, 1H), 7.74–7.67 (m, 1H), 7.33–7.27 (m, 2H), 3.74–3.34 (m, 2H, H13), 3.06 (m, J = 6.0 Hz, 1H, H10), 2.89–2.74 (m, 1H, H15b), 2.63–2.50 (m, 1H, H12), 1.46 and 1.47 (s, J = 8.5 Hz, 3H, H17), 1.47–1.32 (m, 1H, H15a), 0.69 and 0.58 (s, 3H, H16).

**[C2Py](OTf)**. Yield: 45 mg (60%). IR (cm^−1^): 2027s (νCO, sym), 1907w (νCO, asym). ESI-MS (MeOH): *m*/*z*, 599.7 [Re(CO)_3_L2Py]^+^. ^1^H NMR (300 MHz, CD_3_CN) δ 9.23–9.14 (m, 1H, H1), 8.33–8.06 (m, 6H), 7.91–7.78 (m, 1H), 7.72 (d, J = 1 Hz, 1H), 7.33–7.20 (m, 2H), 3.20–3.06 (m, H10, H13), 2.89–2.77 (m, 1H, H15b), 2.43–2.29 (m, 1H, H12), 1.47 and 1.46 (s, 3H, H17), 1.30–1.22 (m, 1H, H15a), 0.69 and 0.60 (s, 3H, H16).

### 3.3. Synthesis of Cationic Complexes with Hexafluorophosphate Counterions: **[C1Py](PF_6_)**, **[C2Py](PF_6_**)

**C1Cl** or **C2Cl** previously obtained (62 mg, 0.1 mmol, 1 eq) were dissolved in anhydrous MeOH (3 mL). Pyridine (18 mg, 18.4 µL, 0.2 mmol, 2 eq) dissolved in MeOH (1 mL) was added, and the flask was covered with aluminum foil to protect the mixture from light. Solid Ag(CF_3_SO_3_) (42 mg, 0.16 mmol, 1.5 eq) was added over this. The reaction mixture was stirred at 65 °C under an inert atmosphere in the absence of light for 16 h and afterward allowed to cool down at RT. The white precipitate, AgCl, was removed by filtration from the yellow solution. The filtrate was concentrated under reduced pressure and dried under high vacuum. The obtained yellow solid was dissolved in MeOH (20 mL) and a saturated NH_4_PF_6_ aqueous solution (5 mL) was added over this. The reaction mixture was stirred at RT for 16 h. The suspension obtained was separated by centrifugation and washed with H_2_O (5 × 10 mL). The solid residue was treated with CHCl_3_ (2 mL) and filtered. The filtrate was dried under high vacuum to give a yellow solid. Several crystallization tests were unsuccessfully attempted for **[C1Py](PF_6_)** and **[C2Py](PF_6_)** (e.g., hexane or pentane layering on CHCl_3_ or CH_2_Cl_2_ solutions of the compounds, slow solvent evaporation of CHCl_3_ or CH_2_Cl_2_ solutions, slow evaporation of the compounds in a 1:1 CDCl_3_:hexane solution or a 1:1 methanol:water solution). No single crystals suitable for X-ray diffraction were obtained.

**[C1Py](PF_6_)**. Yield: 55 mg (67%). IR (cm^−1^): 2027s (νCO, sym), 1900w (νCO, asym). ESI-MS (MeOH): *m*/*z*, 599.7 [Re(CO)_3_L1Py]^+^. ^1^H NMR (300 MHz, CDCl_3_) δ 9.06–9.00 (m, 1H, H1), 8.49–8.40 (m, 1H), 8.34–8.17 (m, 2H), 8.07–8.00 (m, 2H), 7.89–7.75 (m, 2H), 7.66–7.58 (m, 1H), 7.40–7.33 (m, 2H), 3.61–3.19 (m, 2H, H13), 3.04 (m, J = 5.3 Hz, 1H, H10), 2.89–2.74 (m, 1H, H15b), 2.62–2.49 (m, 1H, H12), 1.47 and1.46 (s, J = 9.5 Hz, 3H, H17), 1.31 (s, J = 10.4 Hz, 1H, H15a), 0.72 and 0.54 (s, 3H, H16).

**[C2Py](PF_6_)**. Yield: 46 mg (55%). IR (cm^−1^): 2028s (νCO, sym), 1908w (νCO, asym). ESI-MS (MeOH): *m*/*z*, 599.7 [Re(CO)_3_L2Py]^+^. ^1^H NMR (300 MHz, CDCl_3_) δ 9.05–8.95 (m, 1H, H1), 8.58–8.45 (m, 2H), 8.33 (d, J = 4.4 Hz, 1H), 8.30–8.19 (m, 1H), 8.15–8.04 (m, 2H), 7.88–7.75 (m, 1H), 7.71–7.58 (m, 1H), 7.42–7.29 (m, 2H), 3.44–3.12 (m, 2H, H13), 3.05–2.95 (m, 1H, H10), 2.90–2.72 (m, 1H, H15b), 2.42 (s, 1H, H12), 1.47 and 1.46 (s 3H, H17), 1.36–1.22 (m, 1H, H15b), 0.74 and 0.64 (s, 3H, H16).

### 3.4. Determination of the Minimal Inhibition Concentration (MIC)

The antimicrobial activity of the complexes was evaluated against two strains: *Staphylococcus aureus* SA113 (methicillin-susceptible, MSSA) and *S. aureus* MecA R2954 (methicillin-resistant, MRSA) [62]. Briefly, each complex was initially dissolved in sterile DMSO at a concentration of 10 mM to create a stock solution. The solutions were diluted with Mueller–Hinton Broth (MHB) culture medium and added (serial dilution) to the wells of a 96-well plate at final concentrations ranging from 100 µM to 0.2 µM. Simultaneously, bacterial cultures grown the previous day in non-cation-adjusted MHB (1×) were used to prepare suspensions at 1 × 10^6^ CFU/mL. An equal volume (50 µL) of these bacterial suspensions was added to each well containing the diluted complexes, resulting in final bacterial and compound concentrations of 5 × 10^5^ CFU/mL and 100 µM to 0.2 µM, respectively. The plates were incubated at 37 °C for 24 h. Minimum inhibitory concentrations (MICs) were determined by measuring optical density at 600 nm (OD600). All experiments were performed in triplicate.

### 3.5. Time-Dependent Membrane Potential Measurements

Time-dependent membrane potential measurements were conducted on the MRSA strain using the membrane-sensitive dye 3,3′-diethyloxacarbocyanine iodide (DiOC_2_(3), Sigma), following a previously reported method [15]. Briefly, bacterial cultures were grown to an OD600 of 0.5, then incubated with 30 µM DiOC_2_(3) (from a 3 mM stock in DMSO) for 15 min at 37 °C in the dark. After staining, the bacteria were transferred to a black 96-well plate, and baseline fluorescence was recorded for three minutes using a microplate reader. Following this, the cells were treated with 1% DMSO (negative control), 12.5 µM CCCP (positive control), or the test complexes at concentrations corresponding to 1×, 2×, and 4× the MIC. Fluorescence emissions of DiOC_2_(3) were recorded every minute at red (λ_ex_ = 485 nm, λ_em_ = 635 nm) and green (λ_ex_ = 485 nm, λ_em_ = 530 nm) wavelengths. Emission data were normalized against negative control, and the ratio of normalized red to green fluorescence was plotted.

### 3.6. Cell Toxicity Experiments

The cytotoxicity of the complexes was evaluated using L929 mouse fibroblast cells cultured in DMEM supplemented with 10% (*v*/*v*) fetal bovine serum (FBS), 1% (*v*/*v*) penicillin/streptomycin, and L-glutamine. Cytotoxicity was assessed using the standard MTT assay [63]. Briefly, cells were seeded into 96-well plates at a density of 3.75 × 10^4^ cells/mL and incubated for 24 h. The complexes, initially dissolved in DMSO, were diluted with culture medium and added to the wells at final concentrations ranging from 100 µM to 0.2 µM. After 24 h of treatment, MTT solution (5 mg/mL in PBS) diluted in fresh medium was added to each well at 25% of the total volume and incubated for an additional 4 h. Formazan crystals formed by metabolically active cells were then solubilized in a 1:1 (*v*/*v*) DMSO:ethanol solution. Control wells included untreated cells (100% viability). Relative cell viability was calculated by comparing absorbance values to those of the untreated control.

### 3.7. Bacterial Membrane Integrity Measurement

Propidium iodide (PI) was employed to assess how the complexes affect the bacterial membrane. *S. aureus* MecA R2954 was cultured overnight and then diluted to an OD600 of 0.3 before transferring 1 mL of the culture into Eppendorf tubes. The bacterial samples were then exposed to treatments of 1% DMSO, 100 µg/mL nisin (positive control), as well as to sample concentrations corresponding to 0.5, 1, 2, and 4 times their MIC. These samples were separately incubated at 37 °C with shaking for 30 min, 2 h, or 4 h. After each incubation period, a PI solution (10 µg/mL) was added, and the tubes were further incubated for 5 min. Following this, the bacteria were washed twice with preheated 1× PBS, resuspended in 1× PBS, and then dispensed in triplicate into a black 96-well plate. Finally, the fluorescence emission at 625 nm (485 nm excitation) was recorded using a plate reader.

### 3.8. Statistical Analysis

The bioactivities of the complexes were statistically evaluated using one-way ANOVA followed by Dunnett’s multiple comparisons test, performed with GraphPad Prism 9. The results are expressed as means ± standard deviations (SD), with a *p*-value below 0.05 considered significant. The significance levels are indicated as follows: (*) *p* < 0.03, (**) *p* < 0.02, (***) *p* < 0.0002, and (****) *p* < 0.0001.

### 3.9. In Silico Calculations

Conformation calculations were performed as previously described [5,8] using the AutoDock Vina version 1.2.0 (The Scripps Research Institute, La Jolla, San Diego, CA, USA) [64] and AutoDock4 version 4.2.6 (AD4, The Scripps Research Institute, La Jolla, San Diego, CA, USA) software [65]. Figures were prepared with the AutoDockTools software (version 1.5.7, The Scripps Research Institute, La Jolla, San Diego, CA, USA). Structural parameters of the complex were obtained from crystallographic data. Due to the fact that AD4 failed to assign a change to the metal ion, a charge of 0.320 (to Re) was assigned to the atom by editing the corresponding PDBQT file [66].

The POPE membrane model (fully equilibrated at 303 K in H_2_O) prepared by Jämbeck and Lyubartsev [57] (available at http://www.fos.su.se/~sasha/SLipids/Downloads.html, accessed on 5 February 2025) was used to prepare the semi-flexible 2:1 POPG:POPE membrane model in the conformation analysis. Six central lipids were selected as fully flexible residues (ca. 270 unlocked rotatable bonds), forming an area of ca. 400 Å^2^ (viewed from the top of the membrane facing the cellular exterior, Figure S25). The search space was defined by a box wrapped around the space of the receptors or the fully flexible lipids that also included rigid lipid units. For the membrane model, the search space was defined by a volume of 3.8640 Å3. The number of modes and the exhaustiveness parameter were set to 50 and 30 for the calculations. Such parameters were deemed sufficient for the calculations’ accuracy [67,68,69]. In AutoDock Vina, the electrostatic interactions were handled with the hydrophobic and the hydrogen bonding terms.

## 4. Conclusions

In this study, we synthesized and characterized a series of eight Re(I) tricarbonyl complexes incorporating two enantiopure pinene-bipyridine ligands. Structural elucidation by NMR, mass spectrometry, and X-ray diffraction confirmed the formation of diastereomeric mixtures, underscoring the stereochemical complexity imparted by the chiral ligands. Antimicrobial evaluation against methicillin-resistant and methicillin-sensitive *S. aureus* strains revealed that both cationic and select neutral complexes exhibit potent antibacterial activity, with minimum inhibitory concentrations (MICs) as low as 1.6 μM for the cationic species. Notably, the introduction of chirality via the pinene-bipyridine scaffold resulted in a dramatic enhancement of antibacterial efficacy, up to ca. 60-fold compared to analogous achiral bipyridine complexes, highlighting the pivotal role of ligand chirality in modulating biological activity. Mechanistic investigations demonstrated that while the majority of active complexes did not significantly perturb bacterial membrane potential or metabolic activity, the neutral complexes **C2Br** and **C2Cl** uniquely compromised membrane integrity. In silico docking studies further suggested that *cis* or *trans* diastereomers may interact differently with biological targets, providing a molecular rationale for their enhanced activity and underscoring the influence of stereochemistry on target engagement. Importantly, coordination of the chiral ligands to the rhenium core substantially reduced their cytotoxicity toward mammalian cells, improving the therapeutic window relative to the free ligands. Collectively, these findings establish the critical impact of molecular chirality on the antibiotic properties of Re(I) tricarbonyl complexes and advocate for the continued exploration of chiral ligand scaffolds in the design of next-generation metallopharmaceuticals with improved selectivity and potency.

## Figures and Tables

**Table 1:** In vitro antimicrobial activity and cytotoxicity data of molecules.

	MRSA		MSSA		L929
Compounds	MIC (μM)	Ti	MIC (μM)	Ti	IC_50_ (μM)
**C1Br**	>50	n.t.	>50	n.t.	n.t.
**C2Br**	6.25	1.6	6.25	1.6	10.2
**C1Cl**	>50	n.t.	>50	n.t.	n.t.
**C2Cl**	6.25	1.5	6.25	1.5	9.2
**[C1Py](OTf)**	1.6	11.9	1.6	11.9	19.1
**[C2Py](OTf)**	1.6	15.1	1.6	15.1	24.1
**[C1Py](PF_6_)**	1.6	11.4	1.6	11.4	18.3
**[C2Py](PF_6_)**	1.6	11.9	1.6	11.9	19.1
**[Re(CO)_3_(bpy)Br] ^1^**	>50	n.t.	>50	n.t.	n.t.
**[Re(CO)_3_(bpy)(Py)](OTf)**	100	n.t.	100	n.t.	n.t.

^1^ Mendes et al. reported for the same complex an MIC value of >32 μM against different MSSA and MRSA strains [15]. n.t. stands for “not tested”.

## Data Availability

Data is contained within the article or Supplementary Materials.

## Supplementary Materials

The following supporting information can be downloaded at https://www.mdpi.com/article/10.3390/molecules30153183/s1: Figure S1: ^1^H NMR spectra in CDCl_3_ of **L1**, **C1Cl**, and **C1Br** (from down to top) and peaks assignment.; Figure S2: ^1^H NMR spectra in CDCl_3_ of **L2**, **C2Cl**, and **C2Br** (from down to top) and peaks assignment.; Figure S3: ^1^H NMR spectra in CDCl_3_ of **[C1Py](OTf)** (up) and **[C2Py](OTf)** (down).; Figure S4: ^1^H NMR spectra in CDCl_3_ of **[C1Py](PF_6_)** (up) and **[C2Py](PF_6_)** (down).; Figure S5: ^1^H NMR spectra in CDCl_3_ of the equimolar mixture of the diastereoisomers **[C1Br]** (down) and enriched fractions in one or another diastereoisomer (up).; Figures S6–S13: ESI-MS and HR-MS spectra of compounds.; Figures S14–S17: IR spectra of compounds.; Figure S18: Intermolecular interactions in the single crystal structure of **C1Br**: Br…H contacts are represented as dashed bonds.; Figure S19: Intermolecular interactions in the single crystal structure of **C1Cl**: Cl…H contacts are represented as dashed bonds.; Figure S20: Intermolecular interactions in the single crystal structure of **C2Br**: Br…H contacts are represented as dashed bonds.; Figure S21: Time-dependent effect of selected complexes on the membrane potential of *S. aureus* cells.; Figure S22: Time- and concentration-dependent activity of selected complexes at 0.5×, 1×, and 2× their MIC values on the respiratory chain of *S. aureus* MRSA cells and relative effect of the complexes at time 120 min.; Figure S23: PI fluorescent intensity of *S. aureus* cells treated with DMSO (1%), Nisin (100 µg/mL), and 4 different concentrations of complexes **[C1Py]^+^** and **[C2Py]^+^** at three different time points. Figure S24: Left: lowest binding poses of ***cis*-[C1Py]^+^** and ***cis*-[C1Py]^+^** with *qoxABCD*. Only subunits I and II of *qoxABCD* are shown. Right: binding poses of complexes in *cydAB*. Note how all complexes bind in the same pocket.; Figure S25: Left: top view of the search space and fully flexible 20 × 20 Å area (green lipids) defined in the conformational analysis in the semi-flexible POPG:POPE membrane model. Right: view of the lowest-energy insertion conformation of **C2Br** in the semi-flexible POPG:POPE membrane model showing weak, electrostatic H-bonding interaction between the lipids’ amino and glycero head groups and the compound Br and CO ligands contributing to the conformation’s stabilization. Table S1: X-ray data; Table S2: Bond lengths and distances as determined by X-ray diffraction.
