# Supplementary material for: Pinene-Based Chiral Bipyridine Ligands Drive Potent Antibacterial Activity in Rhenium(I) Complexes
Source: Molecules. 2025 Jul 29;30(15):3183. doi: 10.3390/molecules30153183 (PMC12348105; doi:10.3390/molecules30153183)
Supplement: Supplementary file 1 [file molecules-30-03183-s001.zip › molecules-3739780-supplementary.pdf]

Supporting Information

Pinene-Based Chiral Bipyridine Ligands Drive Potent Antibacterial Activity in Rhenium(I) Complexes

Justine Horner[1,2], Gozde Demirci[1], Aurelien Crochet[1], Aleksandar Pavic[3], Olimpia Mamula Steiner[2,*], Fabio Zobi[1,*]

[1] Department of Chemistry, University of Fribourg, Chemin du Musée 9, CH-1700 Fribourg, Switzerland
[2] University of Applied Sciences of Western Switzerland, HES-SO, HEIA-FR, Pérolles 80, CH-1705 Fribourg, Switzerland
[3] Institute of Molecular Genetics and Genetic Engineering, University of Belgrade, Vojvode Stepe 444a, 11042 Belgrade, Serbia

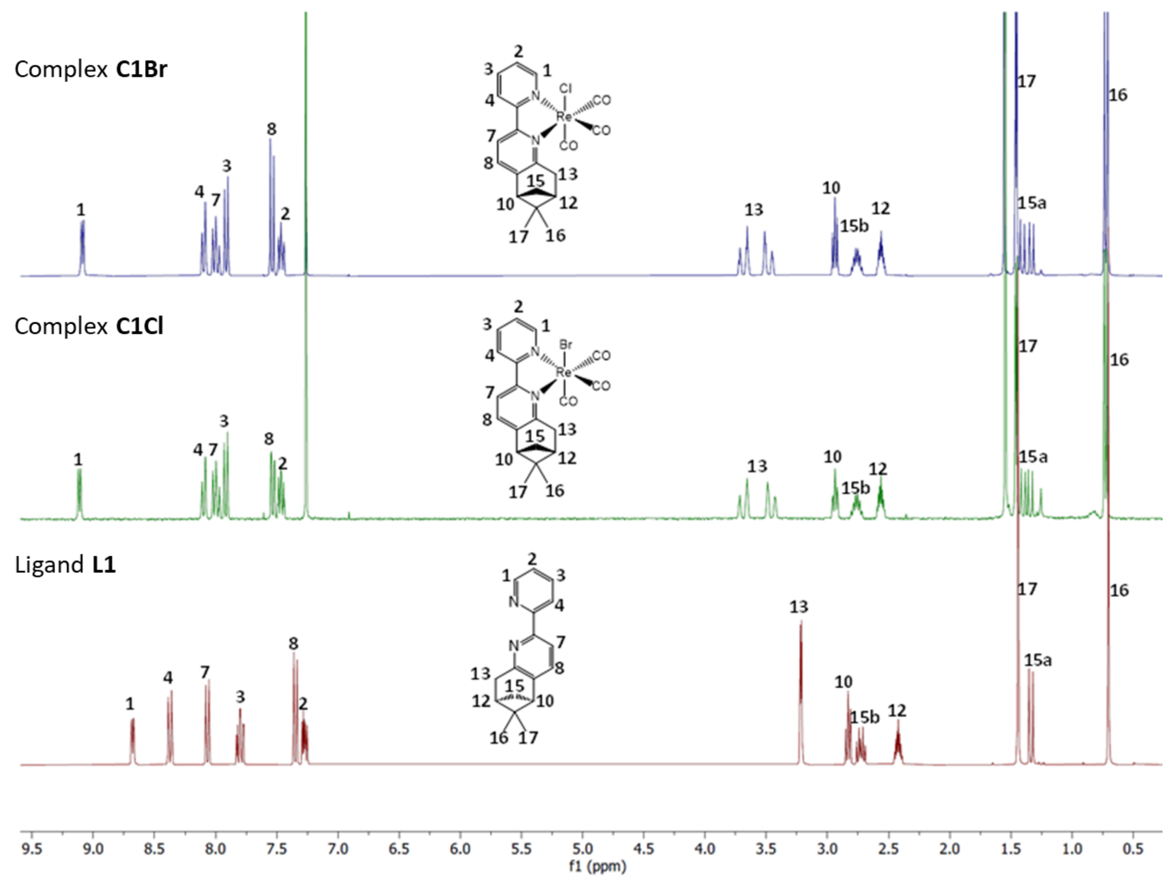

Figure S1. ^{1}H NMR spectra in $CDCl_3$ of **L1**, **C1Cl** and **C1Br** (from down to top) and peaks assignment.

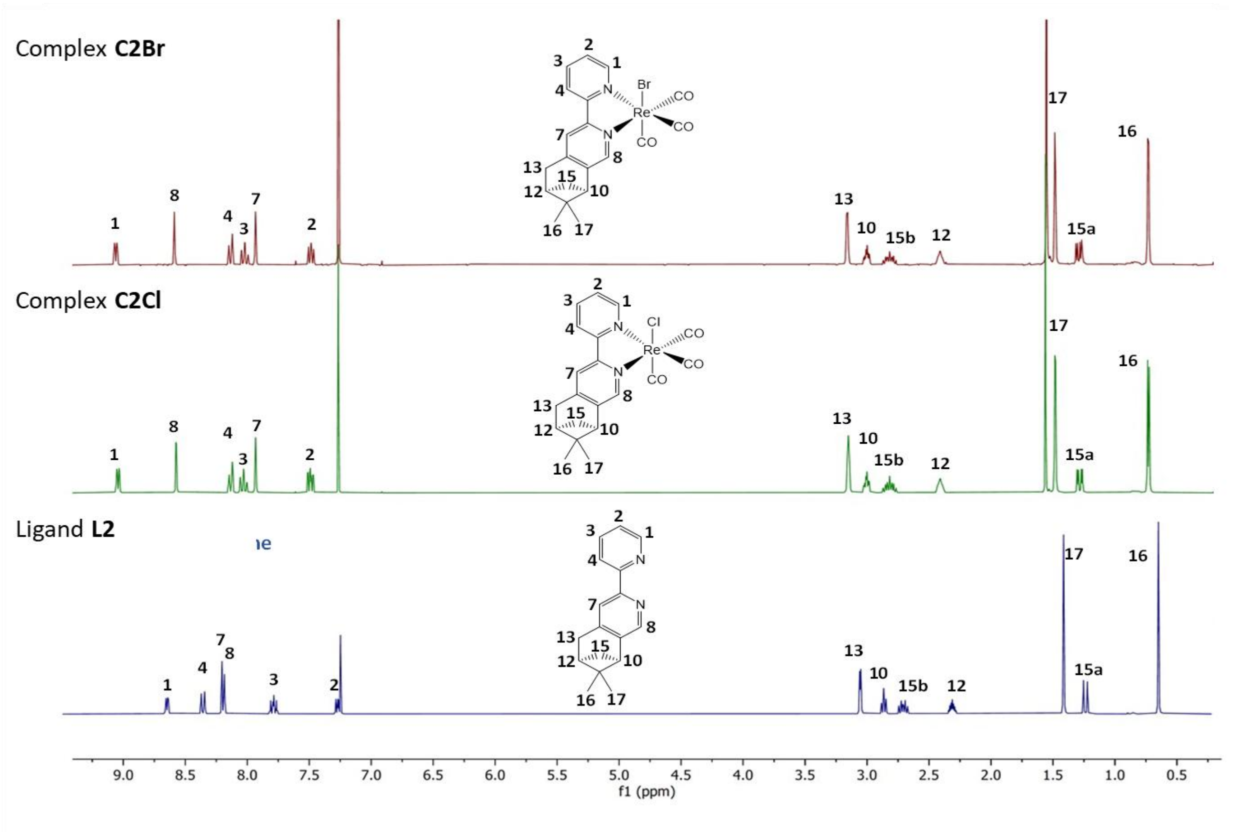

Figure S2. ^{1}H NMR spectra in $CDCl_3$ of **L2**, **C2Cl** and **C2Br** (from down to top) and peaks assignment.

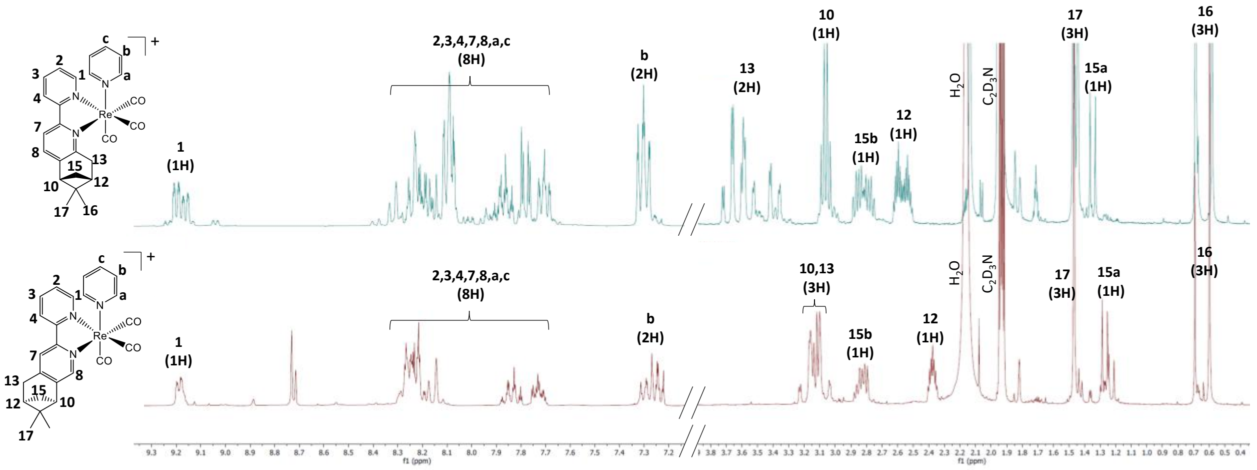

Figure S3. ^{1}H NMR spectra in $CDCl_3$ of **[C1Py](OTf)** (up) and **[C2Py](OTf)** (down).

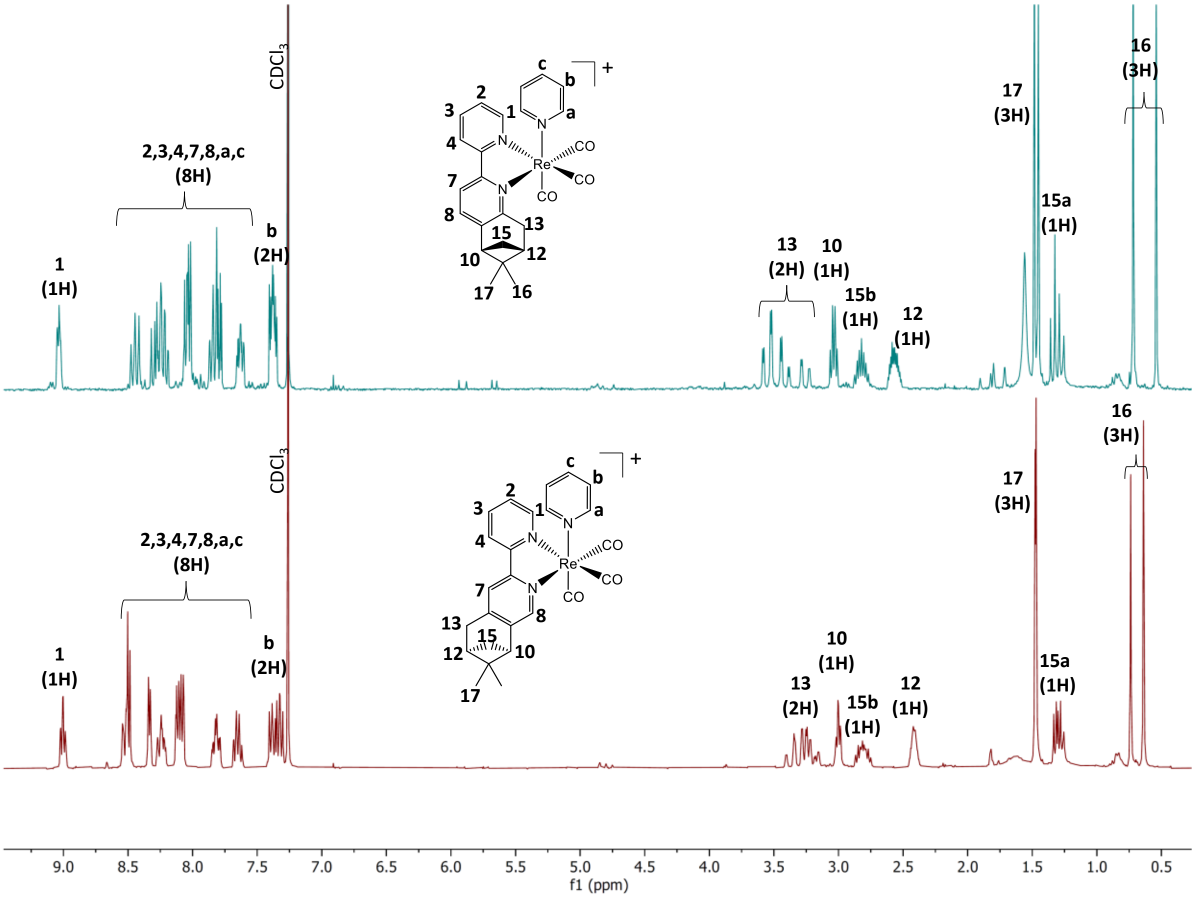

Figure S4. ^{1}H NMR spectra in $CDCl_3$ of **[C1Py](PF_6)** (up) and **[C2Py](PF_6)** (down).

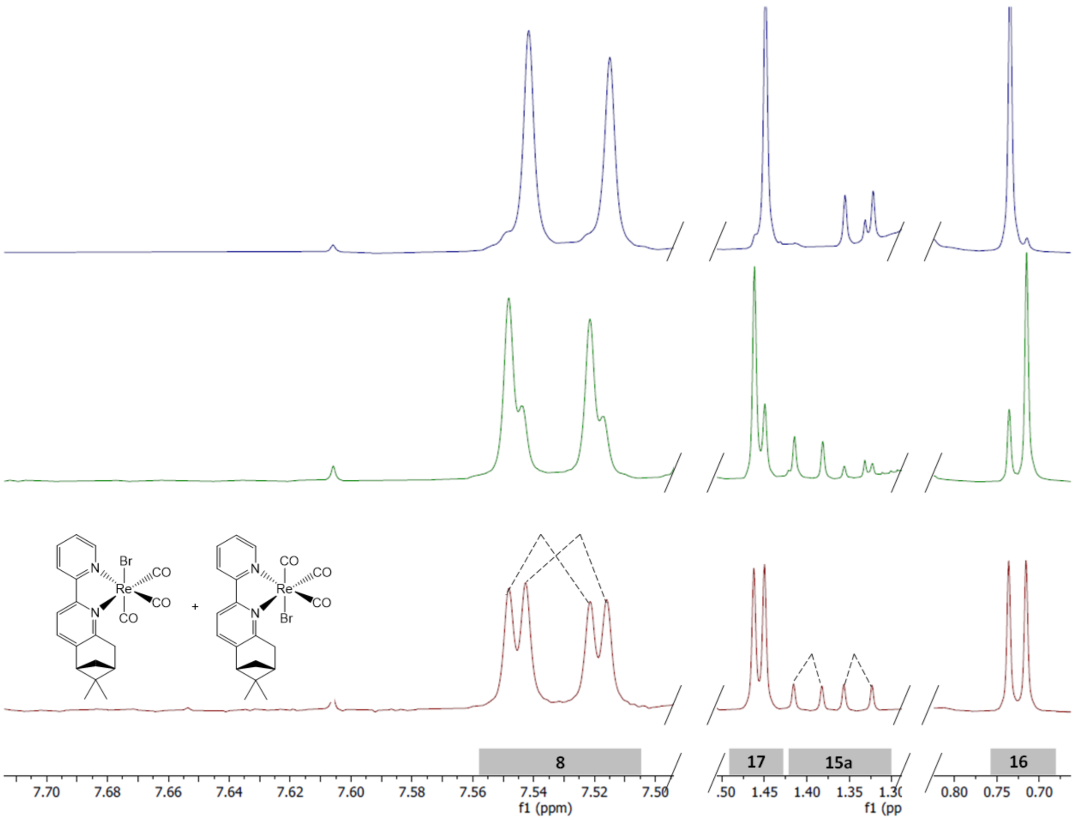

Figure S5. ^{1}H NMR spectra in $CDCl_3$ of the equimolar mixture of the diastereoisomers **[C1Br]** (down) and enriched fractions in one or another diastereoisomer (up)

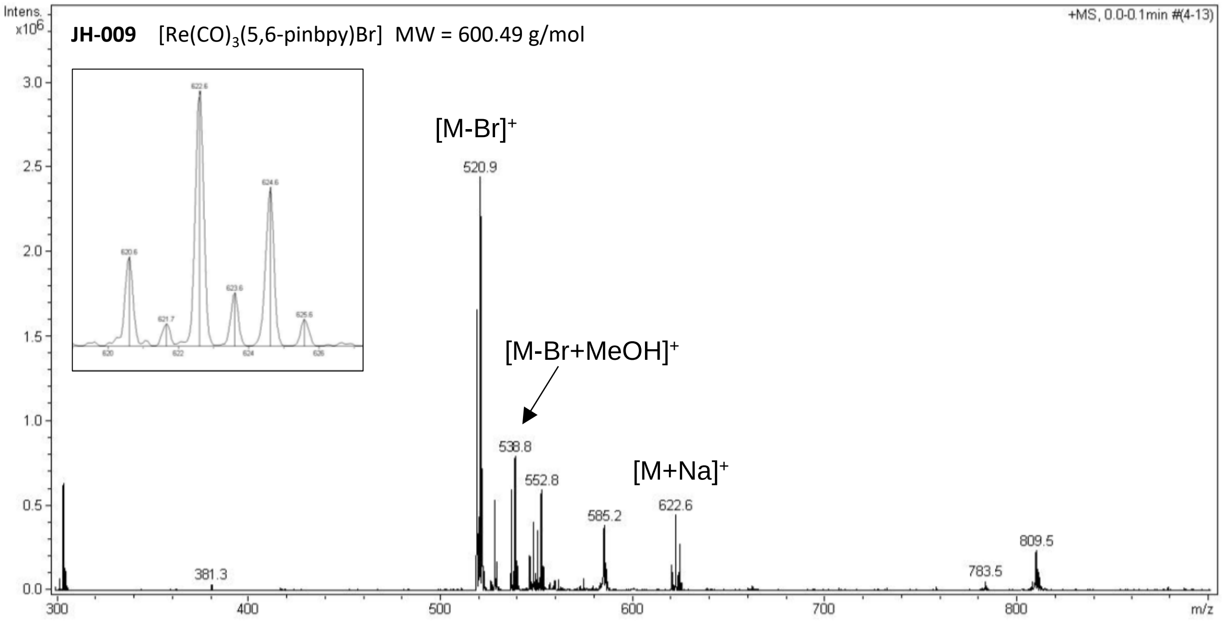

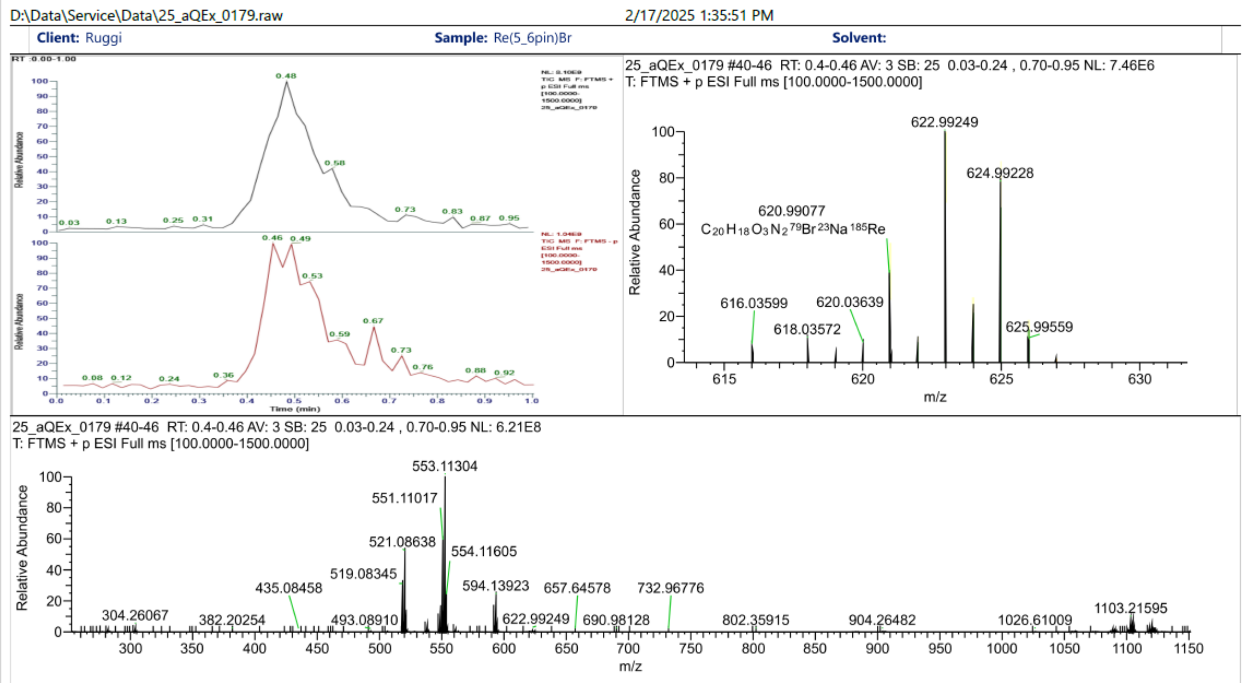

Peak Mass	Display Formula	Theo. mass	Delta [ppm]	RDB	# Matched Iso.	Combined Score	Rank	MSMS Matched Fr...
620.99077	$C_{20}H_{18}O_3N_2{}^{79}Br^{23}Na^{185}Re$	620.99226	-2.40	12.00	7	95.41	1	(Collection)
620.99077	$C_{18}H_{16}O_2N_5{}^{79}Br^{23}Na^{185}Re$	620.99091	-0.24	12.50	7	95.41	2	(Collection)
620.99077	$C_{17}H_{17}O_5N_4{}^{79}Br^{185}Re$	620.99064	0.21	11.00	7	95.25	3	(Collection)
620.99077	$C_{20}H_{15}O_2N_5{}^{79}Br^{185}Re$	620.99332	-4.11	15.50	7	90.76	4	(Collection)
620.99077	$C_{15}H_{18}O_5N_4{}^{79}Br^{23}Na^{185}Re$	620.98823	4.08	8.00	7	50.07	5	(Collection)

Figure S6. ESI-MS spectrum(top) and HR-MS (down) of **C1Br**.

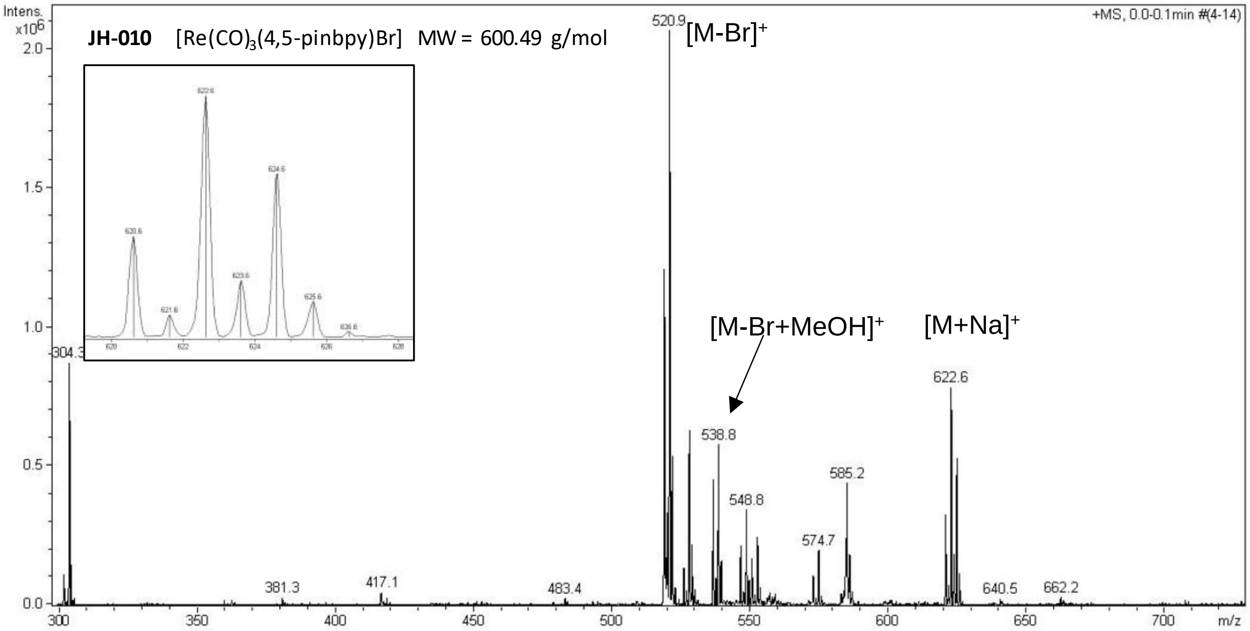

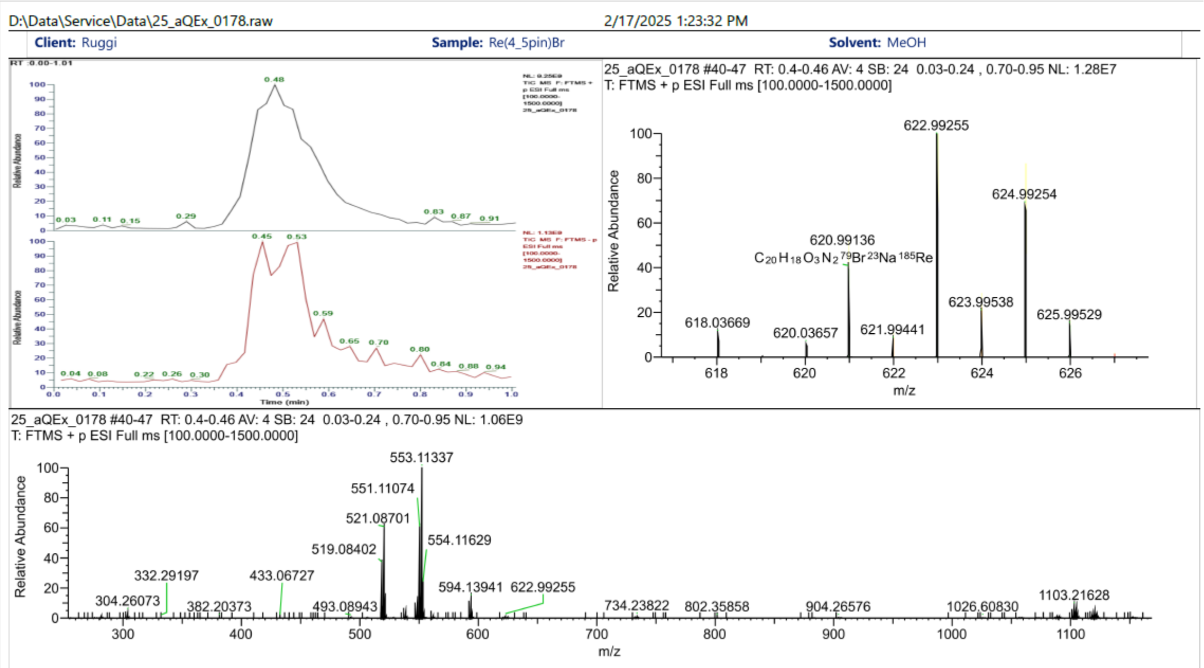

Peak Mass	Display Formula	Theo. mass	Delta [ppm]	RDB	# Matched Iso.	Combined Score	Rank	MSMS Matched Fr...
620.99136	$C_{18}H_{16}O_2N_5{}^{79}Br^{23}Na^{185}Re$	620.99091	0.72	12.50	6	97.41	1	(Collection)
620.99136	$C_{20}H_{18}O_3N_2{}^{79}Br^{23}Na^{185}Re$	620.99226	-1.44	12.00	6	85.74	2	(Collection)
620.99136	$C_3H_{12}O_3N_{17}{}^{79}Br^{23}Na^{185}Re$	620.99142	-0.09	5.50	3	80.53	3	(Collection)
620.99136	$C_4H_{18}O_8N_{10}{}^{79}Br^{23}Na^{185}Re$	620.99142	-0.10	0.00	3	80.29	4	(Collection)
620.99136	$C_{15}H_3O_3N_{13}{}^{23}Na^{185}Re$	620.99036	1.62	20.50	4	46.28	5	(Collection)

Figure S7. ESI-MS spectrum*(top) and HR-MS (down)* of **C2Br***.*

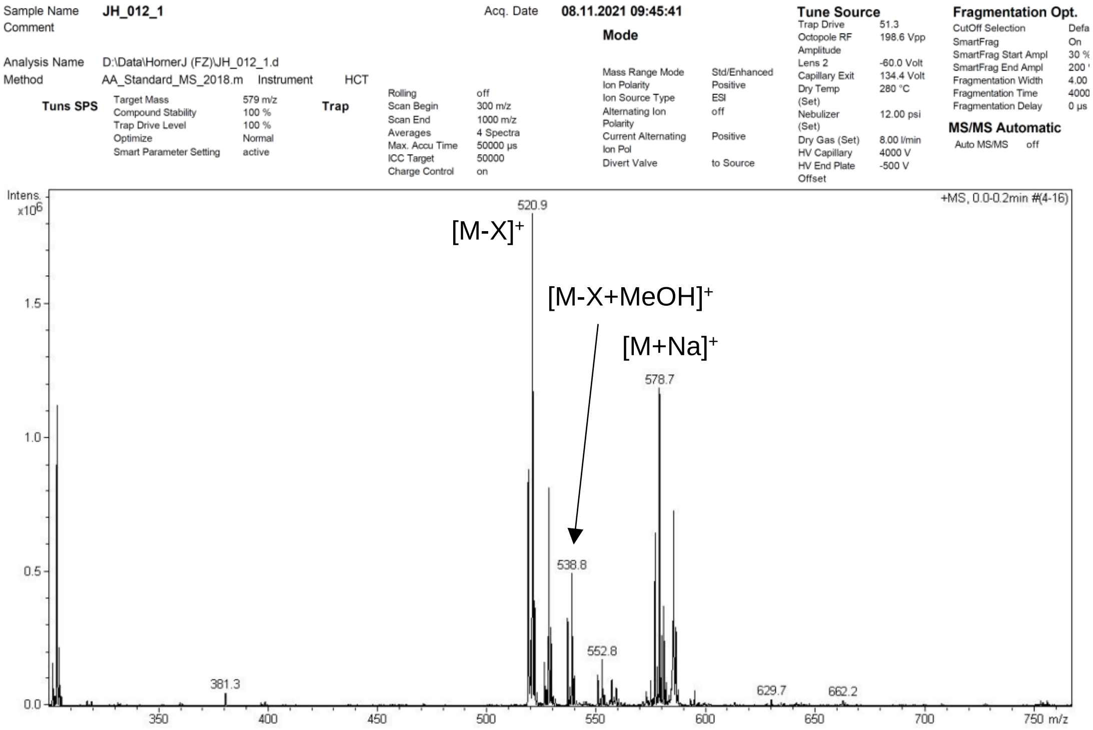

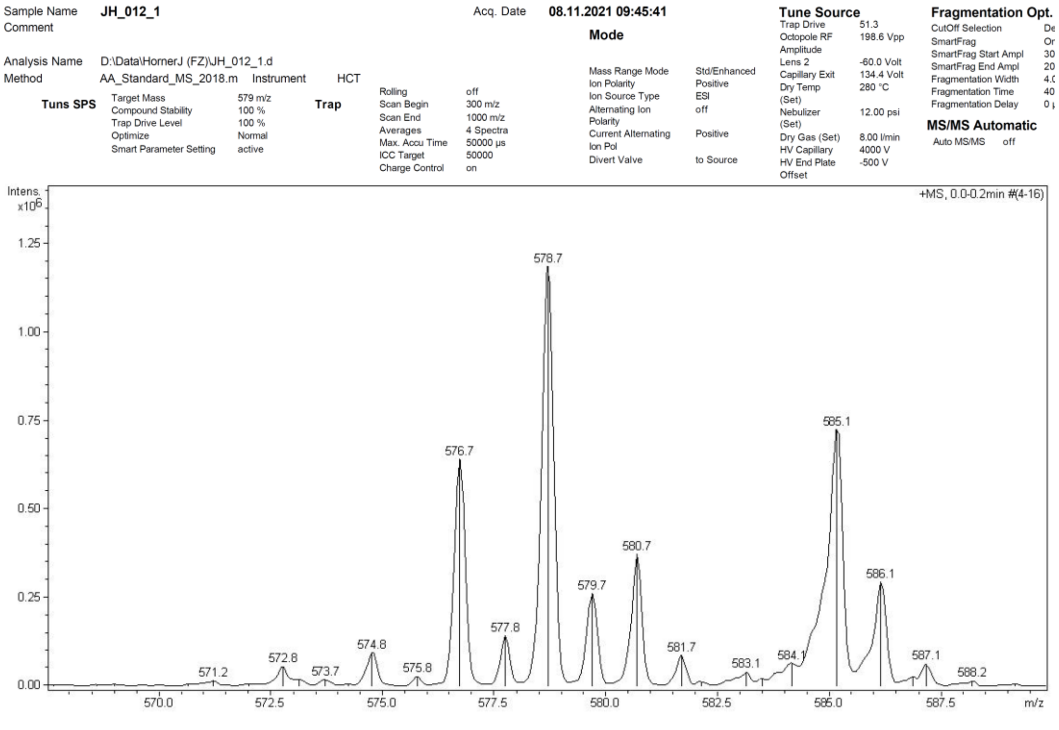

Figure S8. ESI-MS spectrum of **C1Cl**.

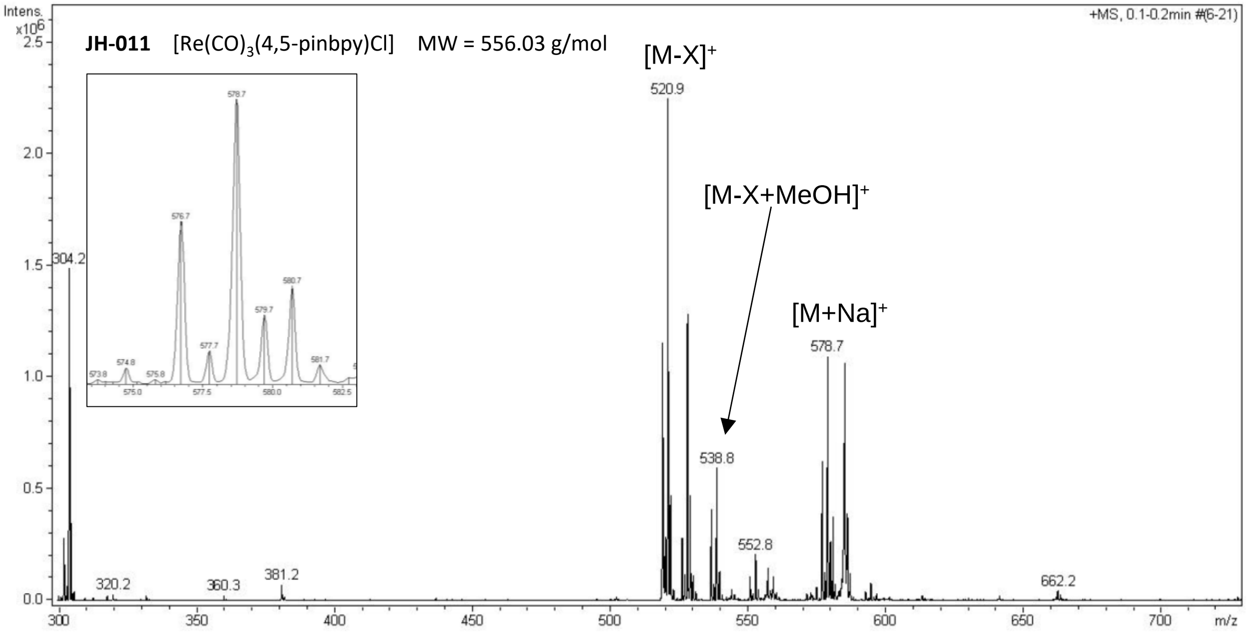

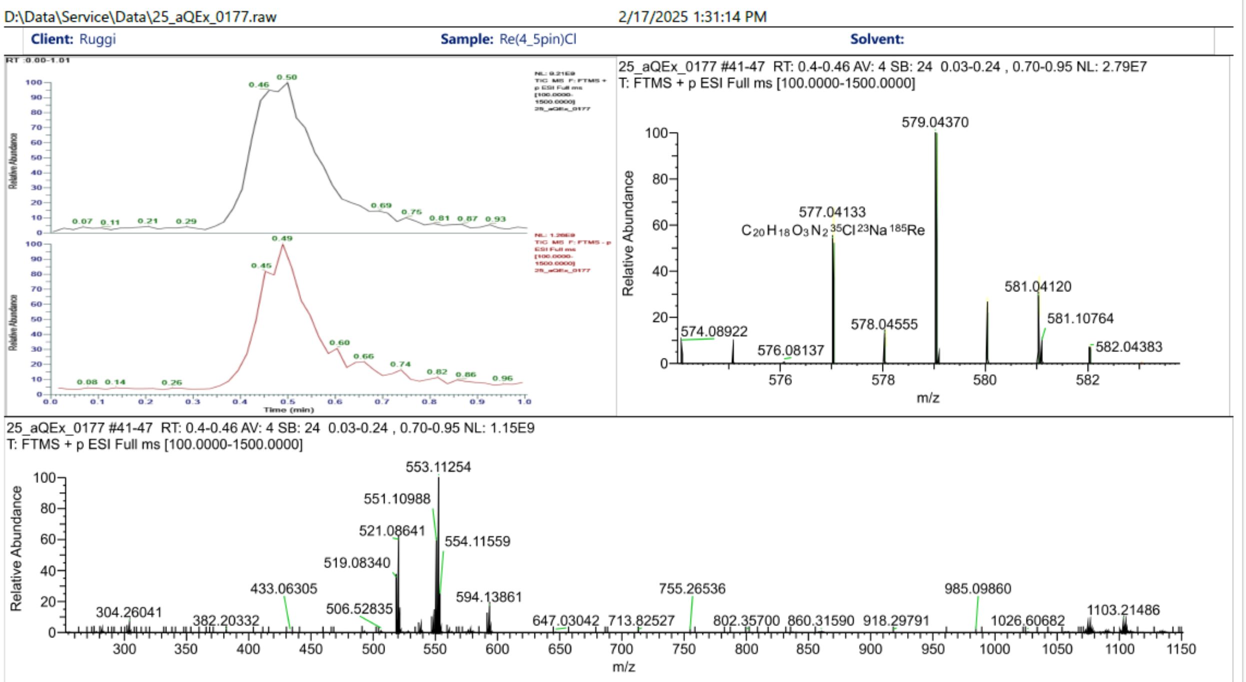

Peak Mass	Display Formula	Theo. mass	Delta [ppm]	RDB	# Matched Iso.	Combined Score	Rank	MSMS Matched Fr...
577.04133	$C_{18}H_{16}O_2N_5{}^{35}Cl^{23}Na{}^{185}Re$	577.04143	-0.17	12.50	6	92.23	1	(Collection)
577.04133	$C_{18}H_{13}ON_8{}^{35}Cl^{185}Re$	577.04249	-2.01	16.00	6	92.18	2	(Collection)
577.04133	$C_{20}H_{18}O_3N_2{}^{35}Cl^{23}Na{}^{185}Re$	577.04277	-2.50	12.00	6	92.07	3	(Collection)
577.04133	$C_{19}H_{19}O_6N^{35}Cl^{185}Re$	577.04250	-2.02	10.50	6	92.04	4	(Collection)
577.04133	$C_{17}H_{17}O_5N_4{}^{35}Cl^{185}Re$	577.04115	0.31	11.00	6	91.84	5	(Collection)

Figure S9. ESI-MS spectrum(top) and HR-MS (down) of **C2Cl**.

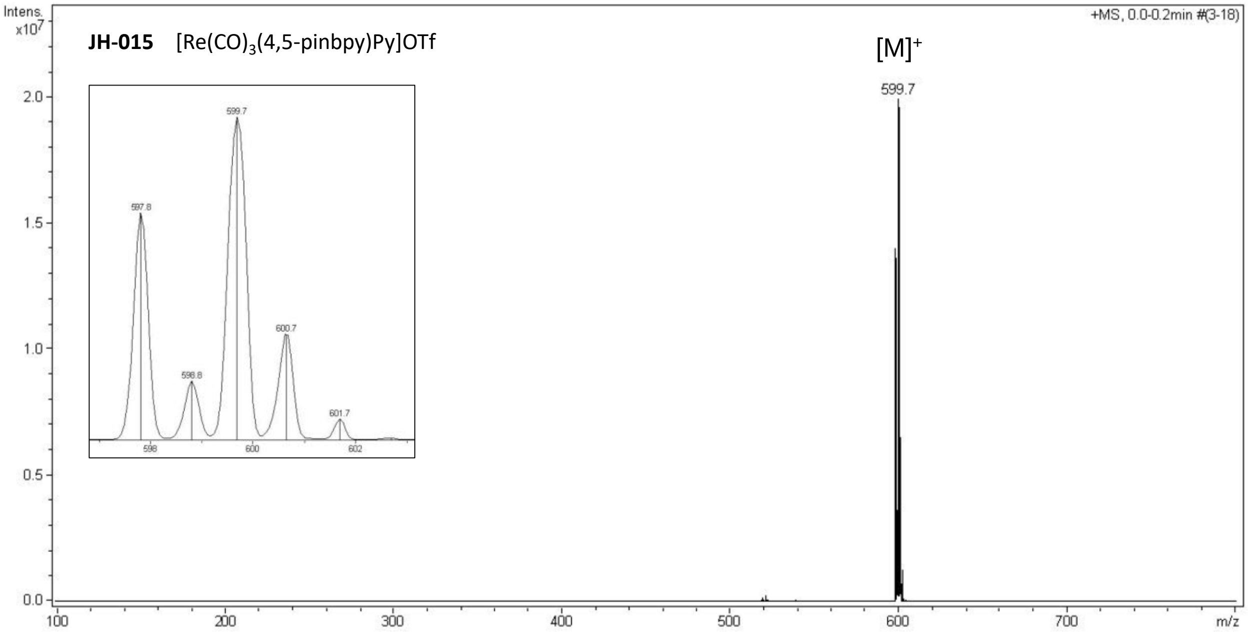

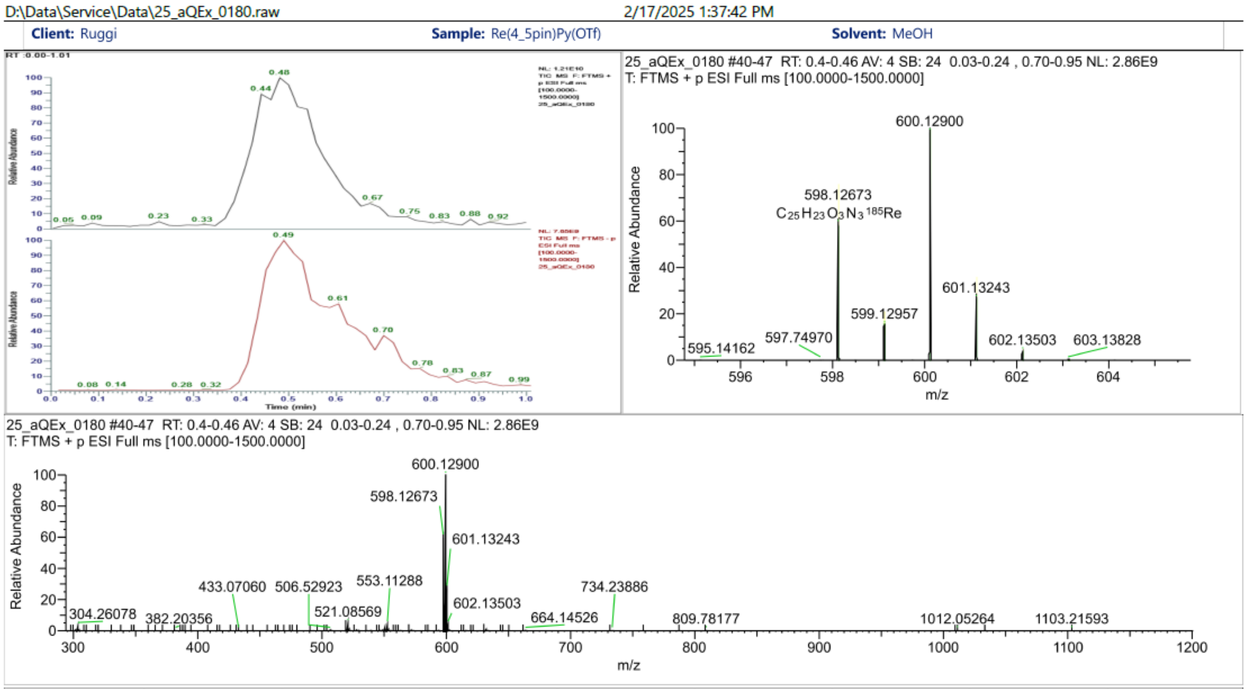

Peak Mass	Display Formula	Theo. mass	Delta [ppm]	RDB	# Matched Iso.	Combined Score	Rank	MSMS Matched Fr...
598.12673	$C_{25}H_{23}O_3N_3{}^{185}Re$	598.12635	0.64	16.00	6	98.91	1	(Collection)
598.12673	$C_{10}H_{19}O_4N_{15}{}^{185}Re$	598.12685	-0.20	9.00	5	74.22	2	(Collection)
598.12673	$C_{11}H_{25}O_9N_8{}^{185}Re$	598.12686	-0.21	3.50	6	74.22	3	(Collection)
598.12673	$C_{27}H_{25}O_4{}^{185}Re$	598.12769	-1.60	15.50	6	27.41	4	(Collection)
598.12673	$C_7H_{20}O_{16}N_{17}$	598.12684	-0.19	6.50	2	0	5	(Collection)

Figure S10. ESI-MS spectrum of **[C2Py]OTf** (in inset: isotopic distribution of the molecular peak $[M]^+$).

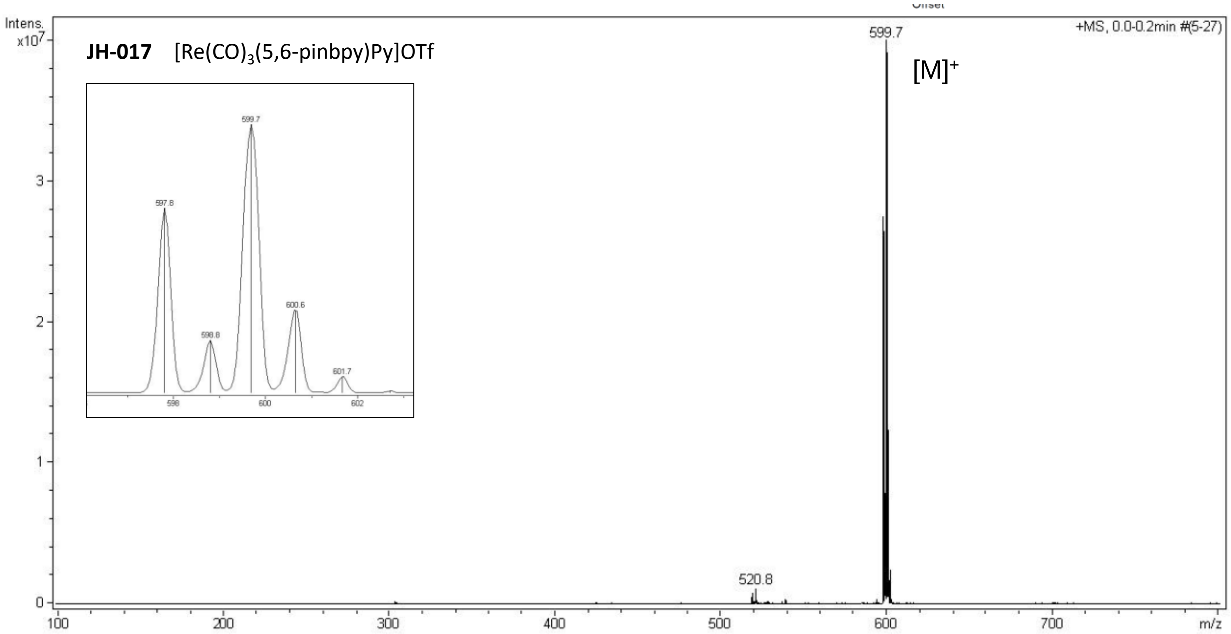

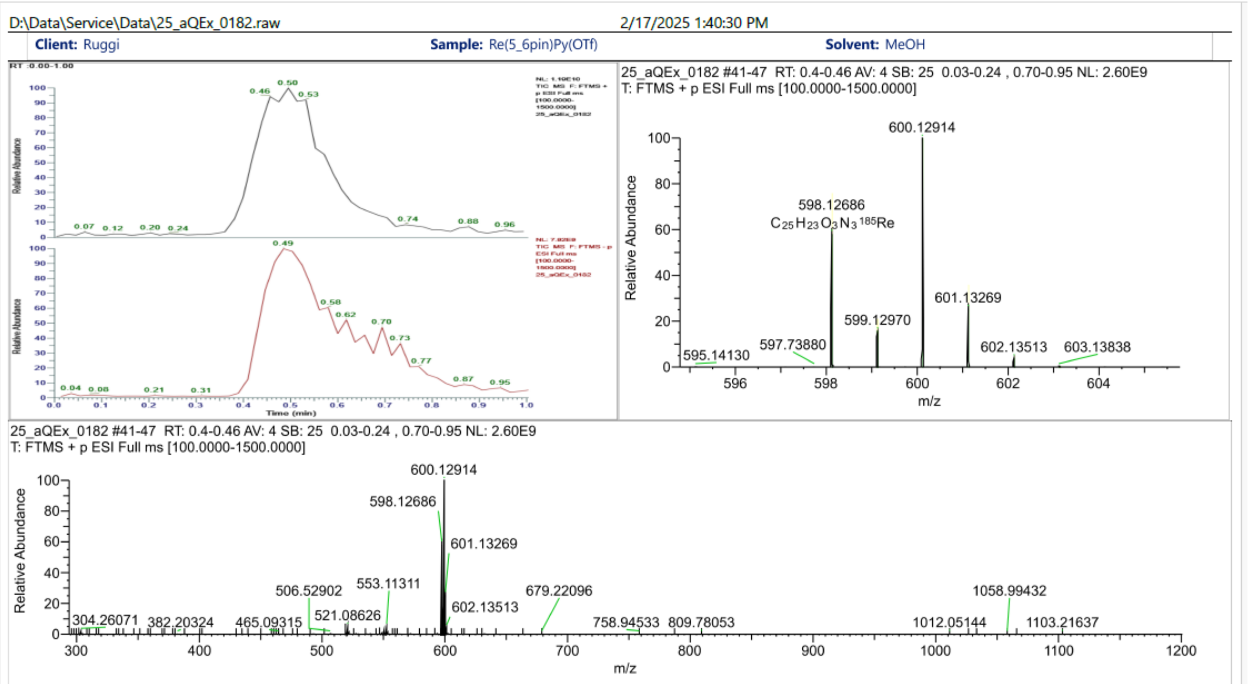

Peak Mass	Display Formula	Theo. mass	Delta [ppm]	RDB	# Matched Iso.	Combined Score	Rank	MSMS Matched Fr...
598.12686	$C_{25}H_{23}O_3N_3{}^{185}Re$	598.12635	0.86	16.00	6	98.84	1	(Collection)
598.12686	$C_{10}H_{19}O_4N_{15}{}^{185}Re$	598.12685	0.02	9.00	4	74.25	2	(Collection)
598.12686	$C_{11}H_{25}O_9N_8{}^{185}Re$	598.12686	0.01	3.50	6	74.24	3	(Collection)
598.12686	$C_{27}H_{25}O_4{}^{185}Re$	598.12769	-1.39	15.50	6	27.43	4	(Collection)
598.12686	$C_7H_{20}O_{16}N_{17}$	598.12684	0.03	6.50	2	0	5	(Collection)

Figure S11. ESI-MS spectrum(top) and HR-MS (down) of **[C1Py]OTf**.

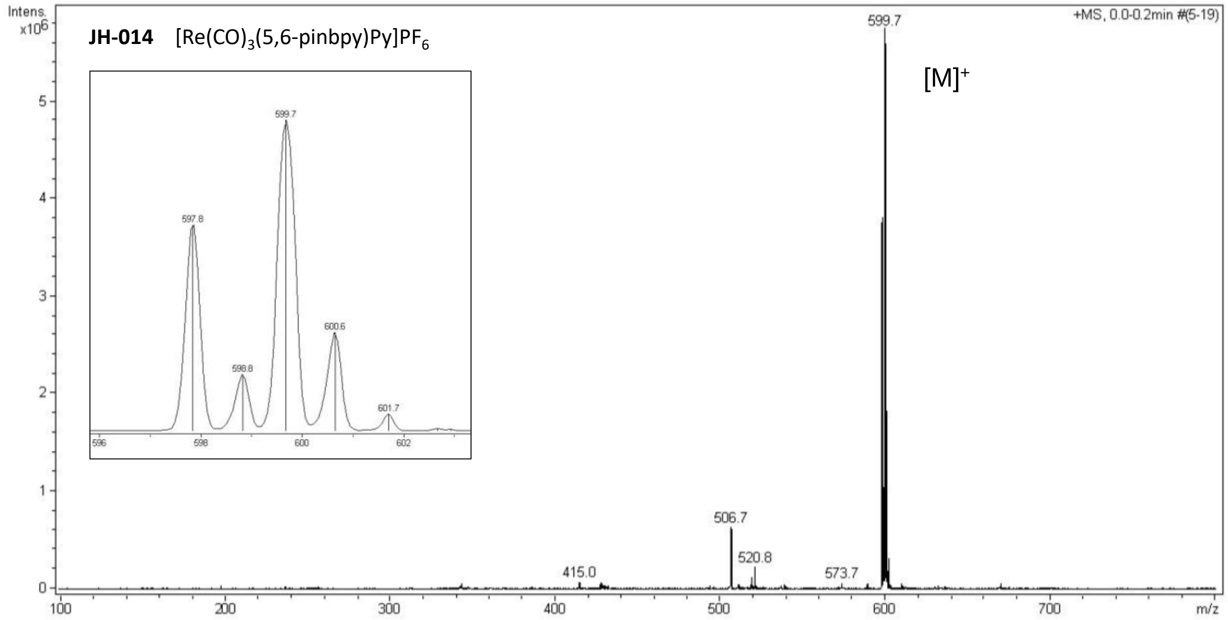

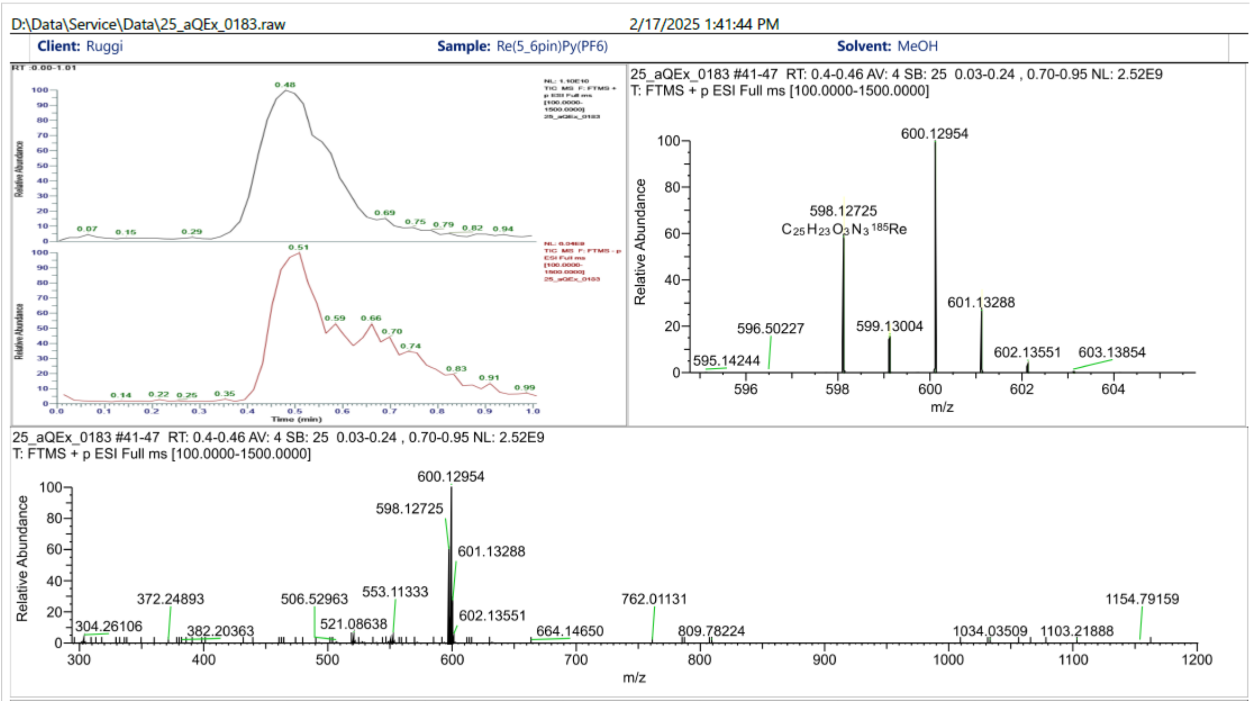

Peak Mass	Display Formula	Theo. mass	Delta [ppm]	RDB	# Matched Iso.	Combined Score	Rank	MSMS Matched Fr...
598.12725	$C_{25}H_{23}O_3N_3{}^{185}Re$	598.12635	1.50	16.00	6	98.11	1	(Collection)
598.12725	$C_{27}H_{25}O_4{}^{185}Re$	598.12769	-0.74	15.50	6	93.99	2	(Collection)
598.12725	$C_{11}H_{25}O_9N_8{}^{185}Re$	598.12686	0.66	3.50	6	74.52	3	(Collection)
598.12725	$C_{10}H_{19}O_4N_{15}{}^{185}Re$	598.12685	0.66	9.00	4	74.52	4	(Collection)
598.12725	$C_{13}H_{27}O_{10}N_5{}^{185}Re$	598.12820	-1.59	3.00	6	29.82	5	(Collection)

Figure S12. ESI-MS spectrum(top) and HR-MS (down) of **[C1Py](PF$_6$)**.

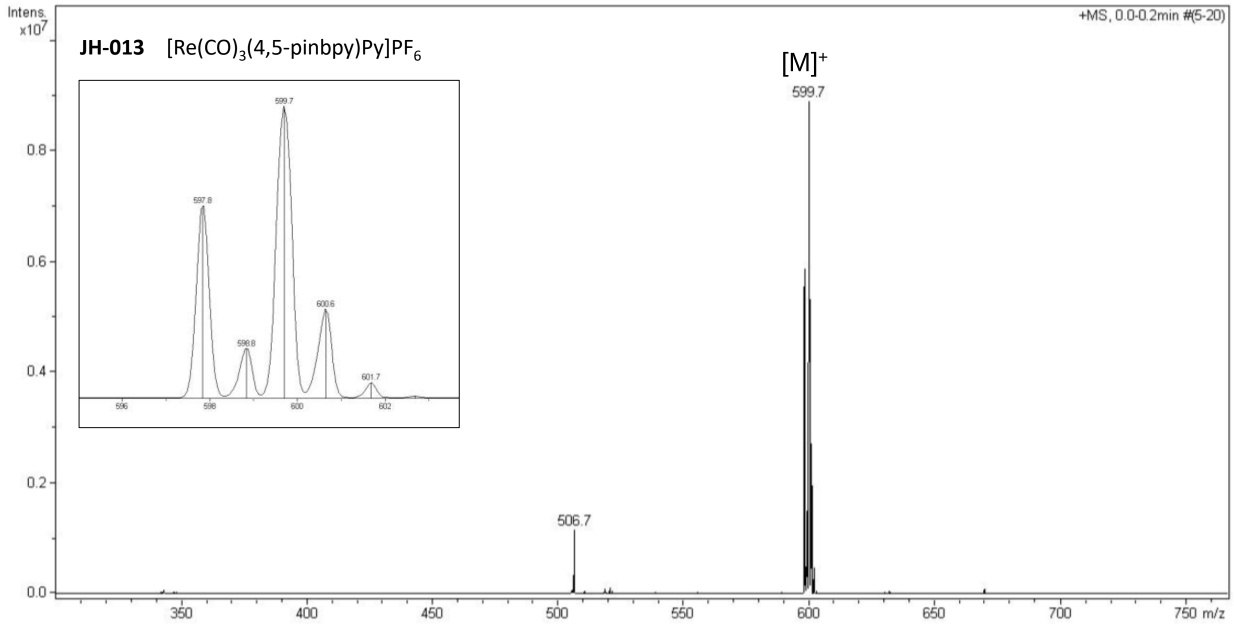

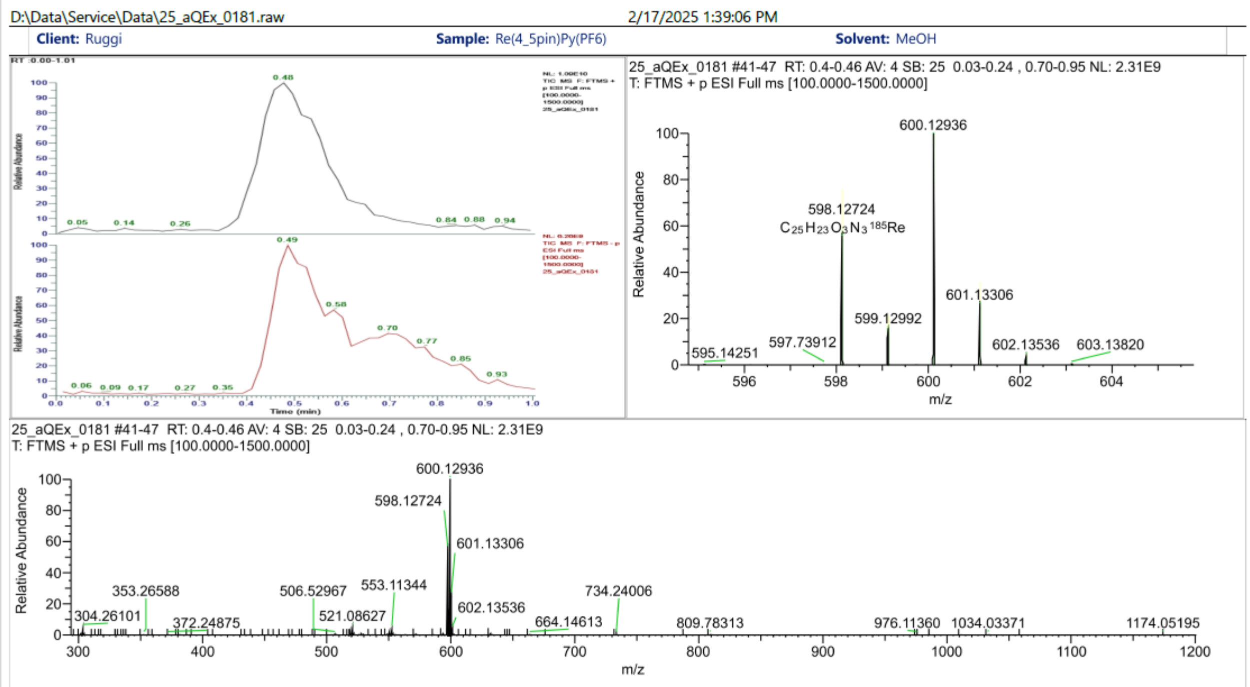

Peak Mass	Display Formula	Theo. mass	Delta [ppm]	RDB	# Matched Iso.	Combined Score	Rank	MSMS Matched Fr...
598.12724	$C_{25}H_{23}O_3N_3{}^{185}Re$	598.12635	1.49	16.00	6	98.2	1	(Collection)
598.12724	$C_{10}H_{19}O_4N_{15}{}^{185}Re$	598.12685	0.65	9.00	5	74.51	2	(Collection)
598.12724	$C_{11}H_{25}O_9N_8{}^{185}Re$	598.12686	0.64	3.50	6	74.51	3	(Collection)
598.12724	$C_{27}H_{25}O_4{}^{185}Re$	598.12769	-0.76	15.50	6	39.14	4	(Collection)
598.12724	$C_{13}H_{27}O_{10}N_5{}^{185}Re$	598.12820	-1.60	3.00	6	29.43	5	(Collection)

Figure S13. ESI-MS spectrum(top) and HR-MS (down) of **[C2Py](PF$_6$)**.

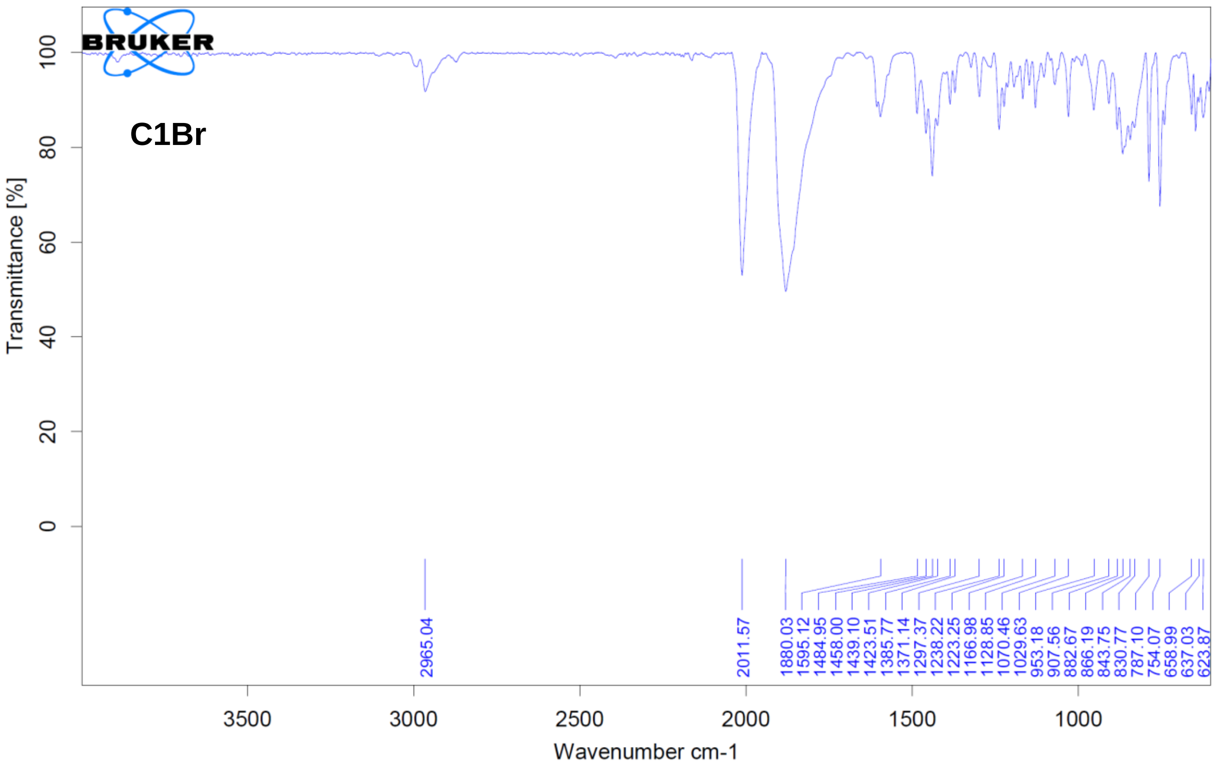

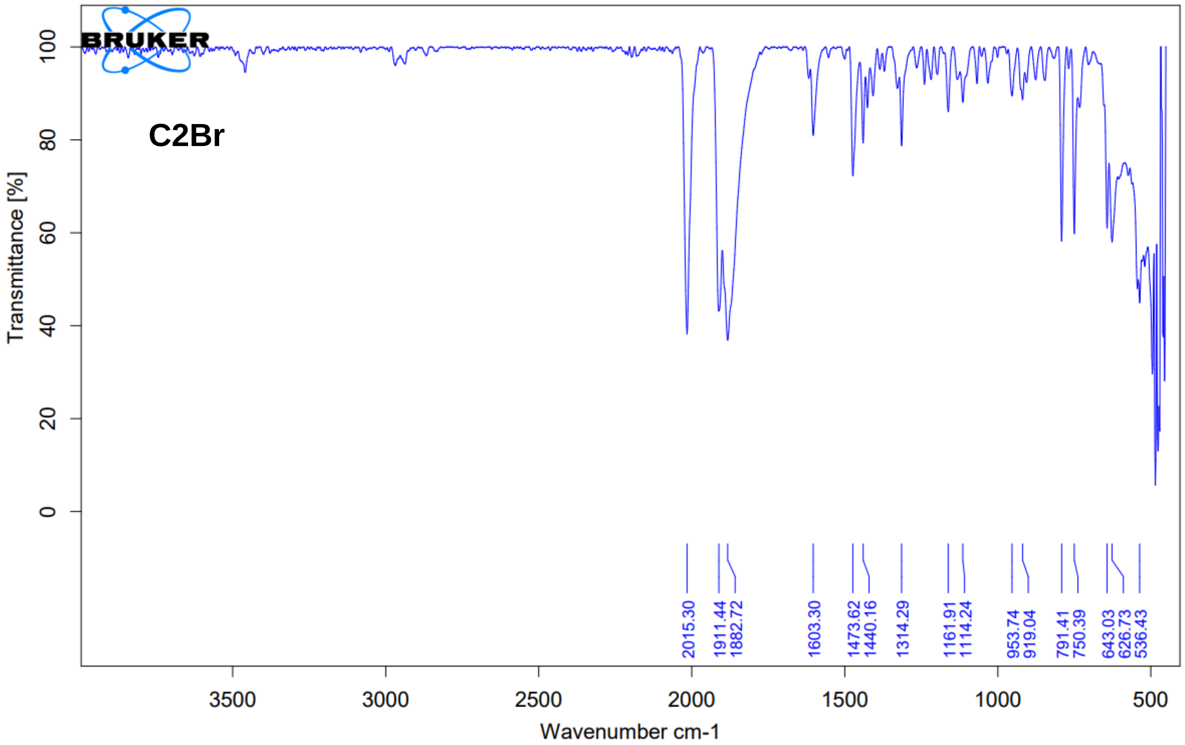

Figure S14. IR specta of **C1Br** and **C2Br**.

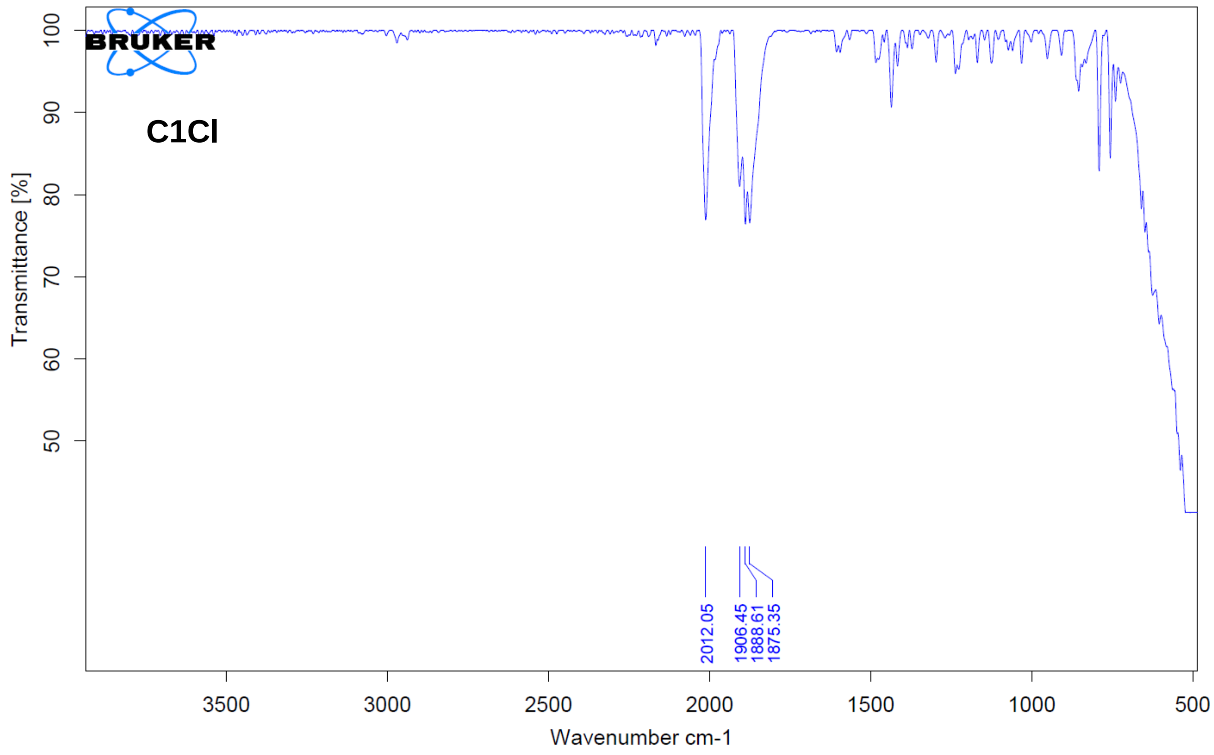

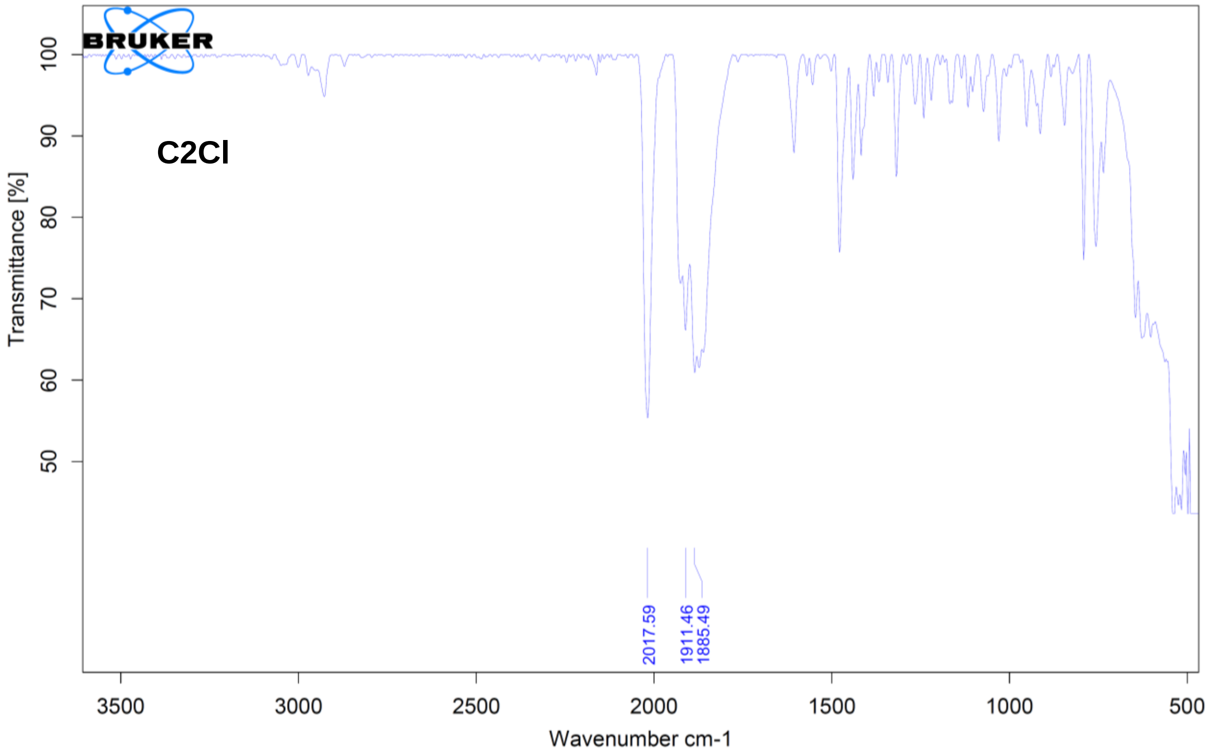

Figure S15. IR specta of **C1Cl** and **C2Cl**.

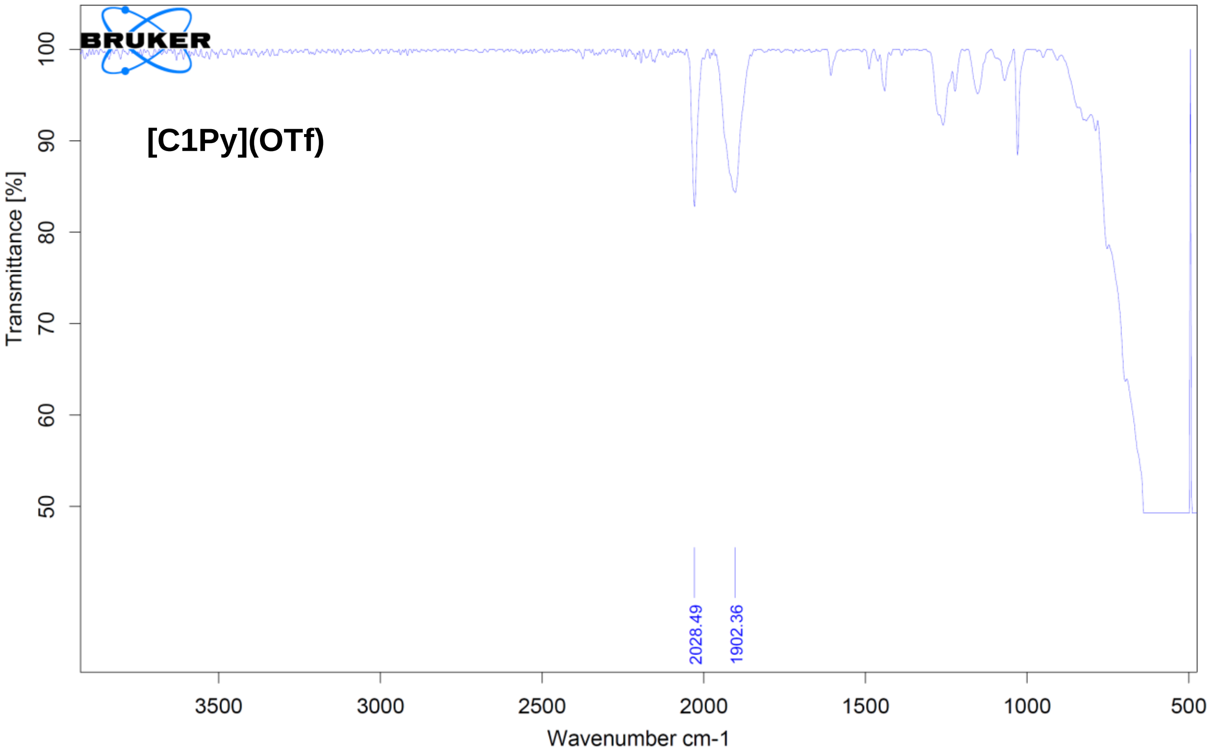

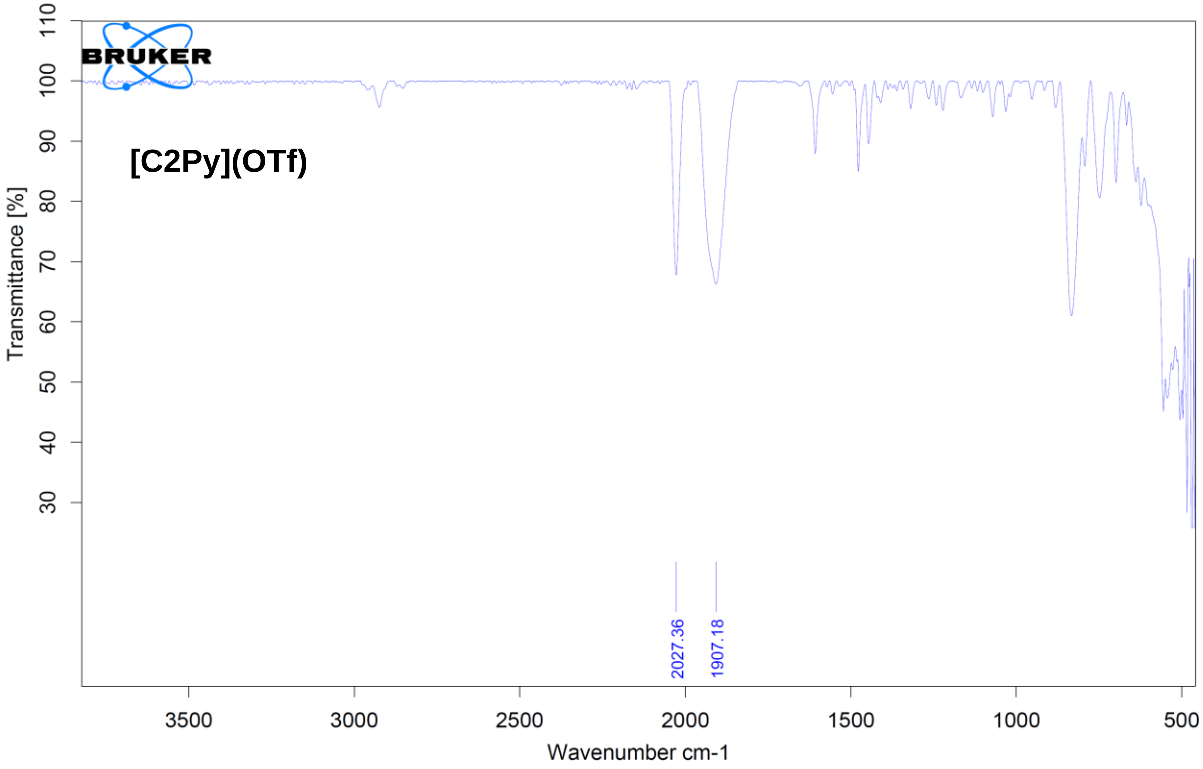

Figure S16. IR specta of **[C1Py](OTf)** and **[C2Py](OTf)**.

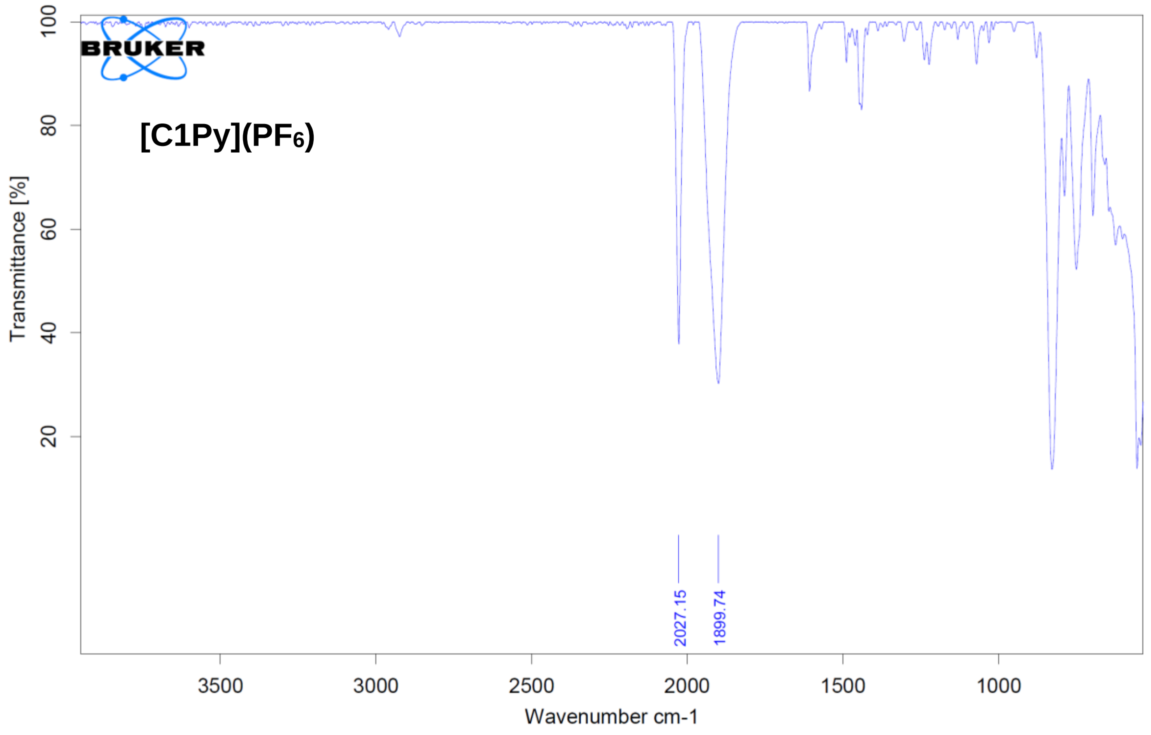

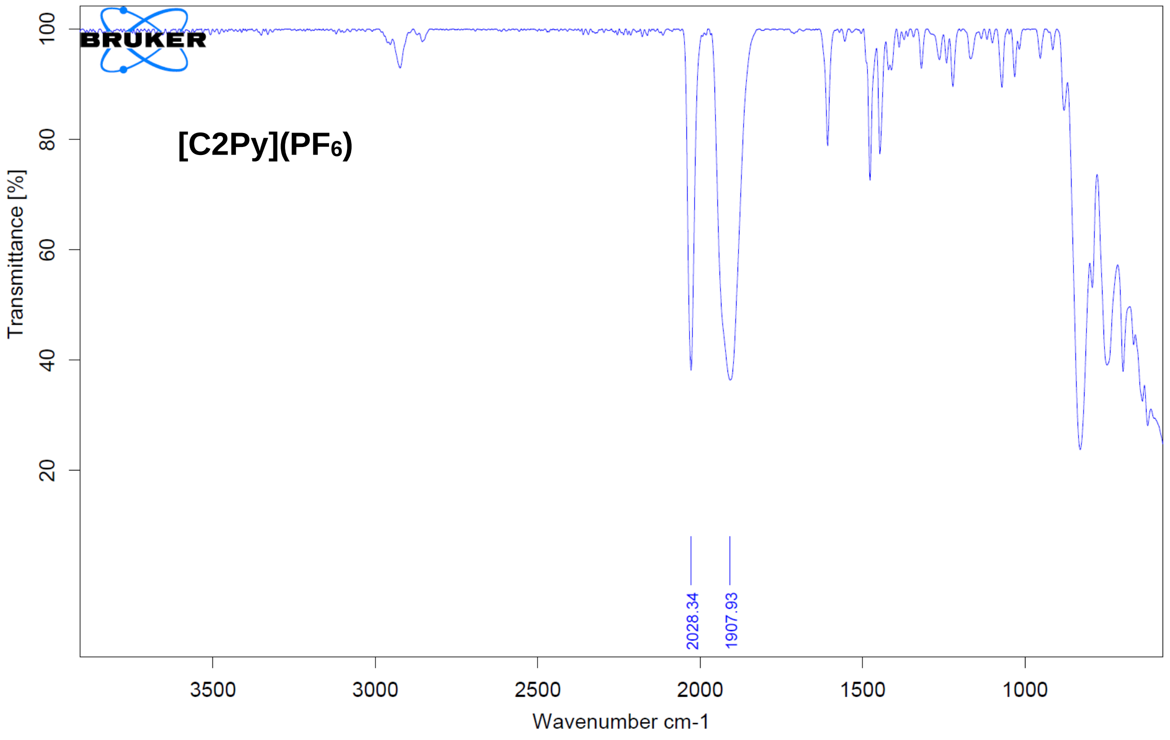

Figure S17. IR specta of **[C1Py](PF$_6$)** and **[C2Py](PF$_6$)**.

Table S1. X-ray data

Composition	[Re(CO)3L1Br]	[Re(CO)$_3$**L1**Cl]	[Re(CO)$_3$**L2**Br]	[Re(CO)$_3$**L2**Py](OTf)	[Re(CO)$_3$**L1**Py](OTf)
ID	**C1Br**	**C1Cl**	**C2Br**	**[C2Py](OTf)**	**[C1Py](OTf)**
CCDC number	2471728	2471729	2471730	2471731	2471738
Formula	$C_{40}H_{36}Br_2N_4O_6Re_2$	$C_{20}H_{18}ClN_2O_3Re$	$C_{21}H_{19}BrCl_3N_2O_3Re$	$C_{26}H_{23}F_3N_3O_6ReS$	$C_{52}H_{46}F_6N_6O_{12}Re_2S_2$
Mol. Weight	1200.95	556.01	719.84	748.73	1497.47
Temperature [K]	250	250	250	250	250(2)
Crystal system	monoclinic	monoclinic	monoclinic	monoclinic	orthorhombic
Space group	$P2_1$	$P2_1$	$P2_1$	$P2_1$	$P2_12_12_1$
a [Å]	13.0509(3)	12.9643(3)	6.8637(2)	10.3305(9)	27.7001(3)
b [Å]	11.1063(2)	11.0520(2)	15.8821(4)	13.2858(8)	15.2103(2)
c [Å]	14.5477(3)	14.4107(3)	11.3929(3)	20.5032(19)	12.91730(10)
α [°]	90	90	90	90	90
β [°]	111.2630(10)	110.669(2)	96.524(2)	91.585(7)	90
γ [°]	90	90	90	90	90
Volume [Å^3]	1965.10(7)	1931.89(7)	1233.90(6)	2813.0(4)	5442.41(10)
Z	2	4	2	4	4
ρ_{calc} [g/cm^3]	2.030	1.912	1.937	1.768	1.828
μ [mm^{-1}]	14.670	13.771	14.730	4.458	10.048
F(000)	1144.0	1072.0	688.0	1464.0	2928.0
Crystal size [mm^3]	0.5 × 0.2 × 0.03	0.666 × 0.25 × 0.03	0.3 × 0.27 × 0.2	0.42 × 0.15 × 0.05	0.65 × 0.293 × 0.1
Radiation [Å]	Cu Kα (λ = 1.54186)	Cu Kα (λ = 1.54186)	Cu Kα (λ = 1.54186)	Mo Kα (λ = 0.71073)	Cu Kα (λ = 1.54186)
2Θ range for data collection [°]	6.52 to 172.192	7.288 to 176.212	11.142 to 135.106	3.654 to 52.432	12.068 to 136.282
Index ranges	$-16 \le h \le 16$, $-12 \le k \le 9$, $-15 \le l \le 15$	$-16 \le h \le 16$, $-12 \le k \le 9$, $-15 \le l \le 14$	$-8 \le h \le 7$, $-18 \le k \le 13$, $-13 \le l \le 13$	$-12 \le h \le 12$, $-16 \le k \le 15$, $-25 \le l \le 25$	$-32 \le h \le 32$, $-17 \le k \le 17$, $-11 \le l \le 14$
Reflections collected	40927	52240	19681	37918	72809
Independent reflections	5132 [R_{int} = 0.0245, R_{sigma} = 0.0127]	5408 [R_{int} = 0.0259, R_{sigma} = 0.0111]	3344 [R_{int} = 0.0505, R_{sigma} = 0.0296]	10795 [R_{int} = 0.0533, R_{sigma} = 0.0405]	9422 [R_{int} = 0.0291, R_{sigma} = 0.0129]
Data/restraints/parameters	5132/1/491	5408/1/492	3344/1/283	10795/1/726	9422/0/725
Goodness-of-fit on F^2	1.080	1.094	1.116	1.068	1.027
Final *R* indexes [I>=2σ (I)]	R_1 = 0.0203, wR_2 = 0.0504	R_1 = 0.0173, wR_2 = 0.0450	R_1 = 0.0439, wR_2 = 0.1179	R_1 = 0.0404, wR_2 = 0.0944	R_1 = 0.0276, wR_2 = 0.0745
Final R indexes (all data)	R_1 = 0.0206, wR_2 = 0.0506	R_1 = 0.0174, wR_2 = 0.0451	R_1 = 0.0440, wR_2 = 0.1181	R_1 = 0.0498, wR_2 = 0.0985	R_1 = 0.0279, wR_2 = 0.0747
Largest diff. peak/hole [e Å^{-3}]	0.38/-1.23	0.94/-0.73	1.93/-1.68	0.72/-0.96	0.87/-0.69
Flack parameter	-0.005(7)	-0.037(8)	0.08(2)	-0.010(13)	-0.012(4)

Table S2. Bond lengths and distances as determined by X-ray diffraction.

	**C1Br**		**C1Cl**		**C2Br**	**[C1Py](OTf)**		**[C2Py](OTf)**	
Diastereomer	Trans	Cis	Trans	Cis	Trans	Trans	Cis	Trans	Cis
M-N(Bpy-pin)	2.222(5)	2.232(5)	2.235(4)	2.231(4)	2.196(11)	2.180(9)	2.180(8)	2.219(5)	2.204(6)
M-N(Bpy)	2.071(4)	2.056(4)	2.076(4)	2.056(4)	2.146(10)	2.177(9)	2.176(9)	2.169(6)	2.160(7)
M-Br	2.7355(6)	2.7737(6)	-	-	2.62(13)	-	-	-	-
M-Cl	-		2.5730(12)	2.6062(13)				-	-
M-Py	-	-	-	-	-	2.218(12)	2.218(11)	2.214(6)	2.211(7)
M-CO(1)	1.925(8)	1.908(8)	1.921(7)	1.912(7)	1.887(15)	1.912(18)	1.881(18)	1.903(8)	1.930(9)
M-CO(2)	1.875(6)	1.853(7)	1.875(6)	1.855(6)	1.921(15)	1.915(14)	1.916(14)	1.908(9)	1.901(8)
M-CO(3)	2.014(6)	2.002(6)	2.011(6)	2.003(6)	1.920(16)	1.926(13)	1.913(14)	1.923(8)	1.923(8)

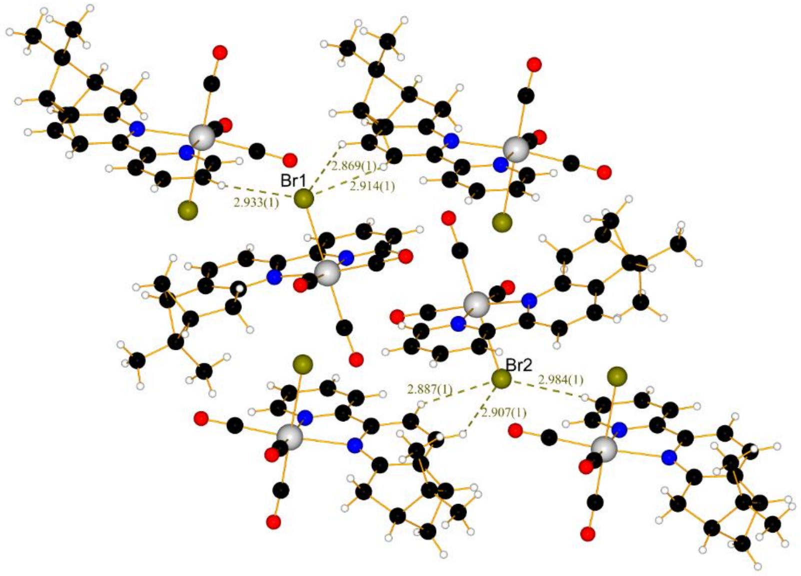

Figure S18. Intermolecular interactions in the single crystal structure of **C1Br**: Br...H contacts are represented as dash bond.

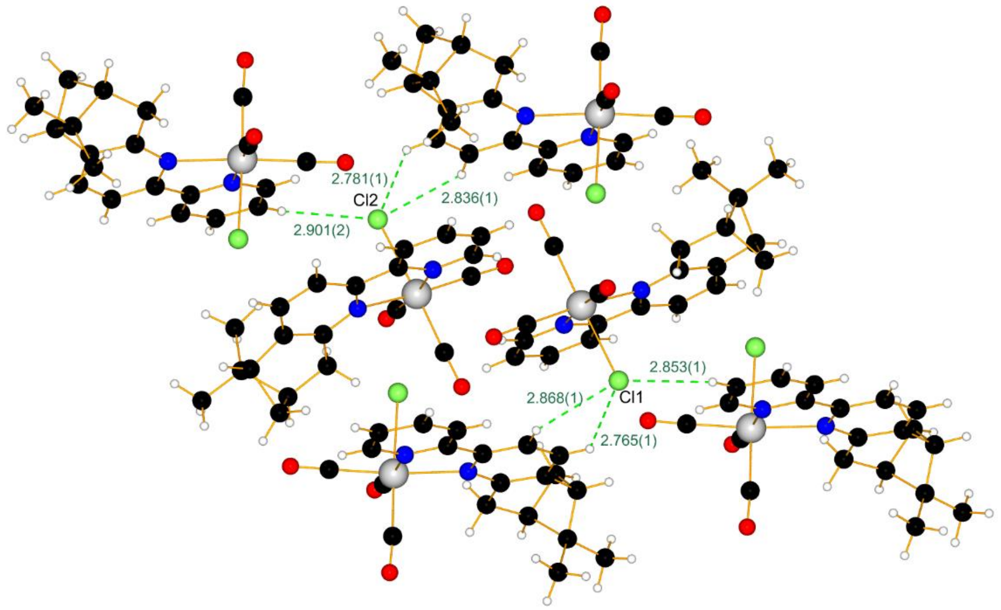

Figure S19. Intermolecular interactions in the single crystal structure of **C1Cl**: Cl…H contacts are represented as dash bond.

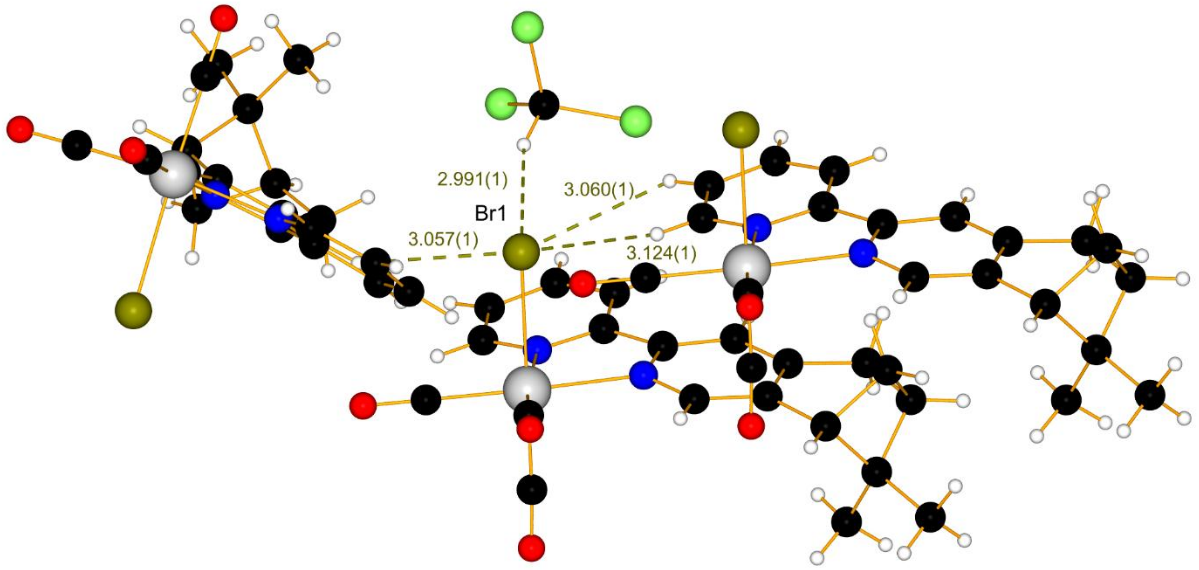

Figure S20. Intermolecular interactions in the single crystal structure of **C2Br** crystallized with one molecule of $CHCl_3$: Br…H contacts are represented as dash bond.

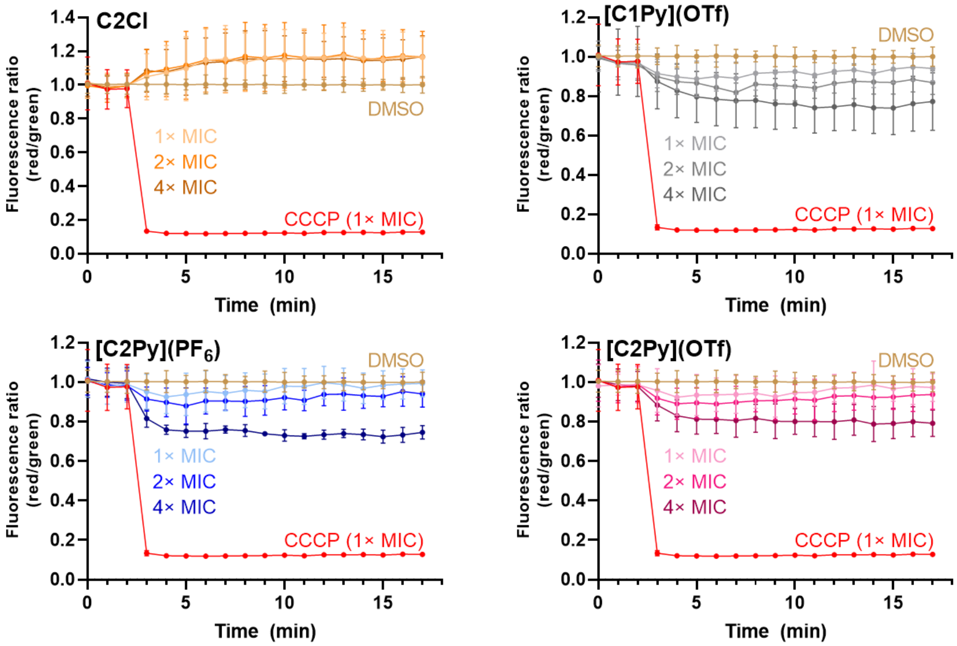

Figure S21. Time-dependent effect of selected complexes on the membrane potential of *S. aureus* cells. Membrane potential was measured as indicated in the experimental section (see also Figure 6 caption in the manuscript). #× labels indicate ×MIC value of the complexes. Data represent the mean of three biological replicates, with error bars indicating the standard deviation of the mean.

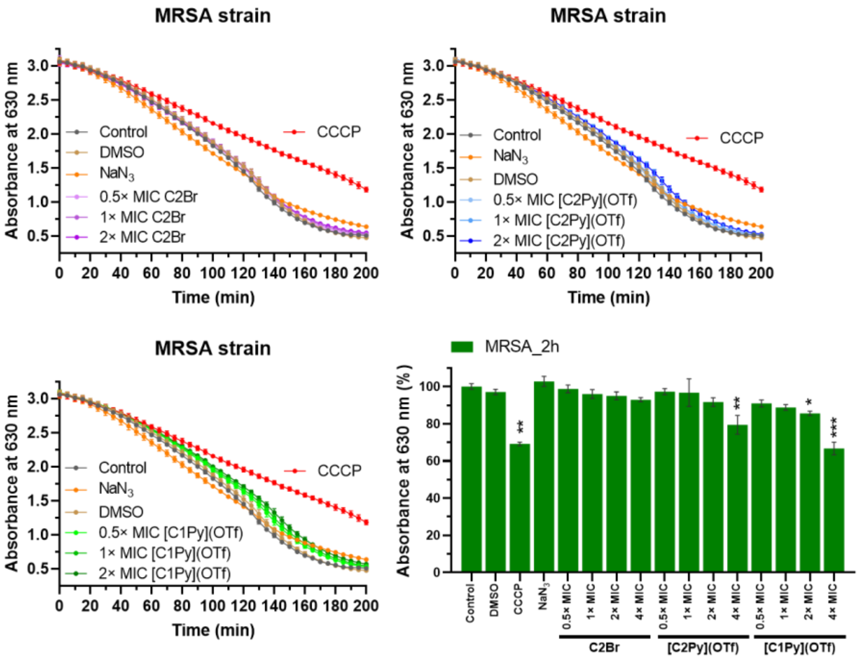

Figure S22. Time- and concentration-dependent activity of selected complexes at 0.5, 1 and 2× their MIC values on the respiratory chain of *S. aureus* MRSA cells and relative effect of the complexes at time 120 min.

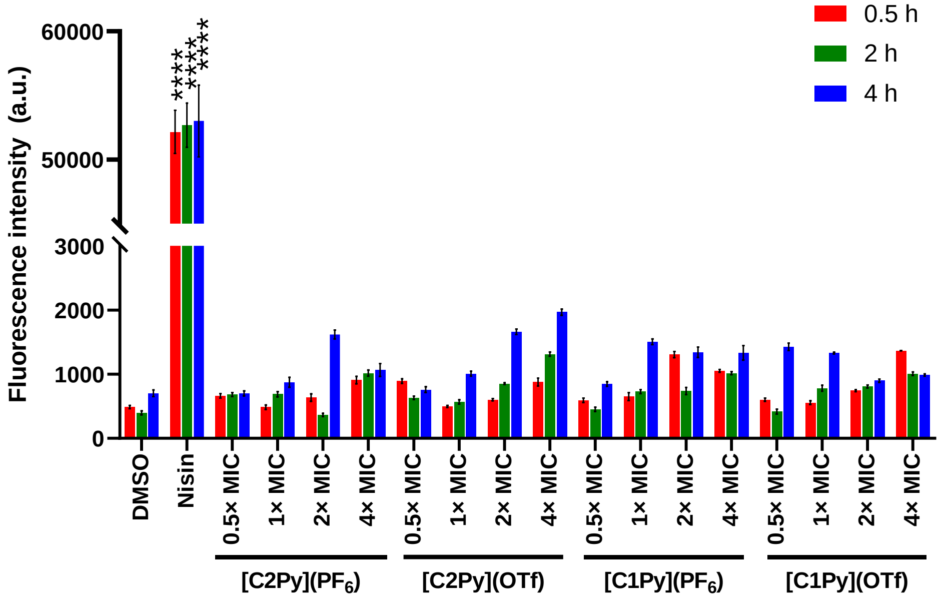

Figure S23. PI fluorescent intensity of *S. aureus* cells treated with DMSO (1%), Nisin (100 µg/mL) and 4 different concentrations of complexes [C1Py]$^+$ and [C2Py]$^+$ at three different time points. Data represent the mean of three biological replicates, with errors bars indicating the standard deviation of the mean.

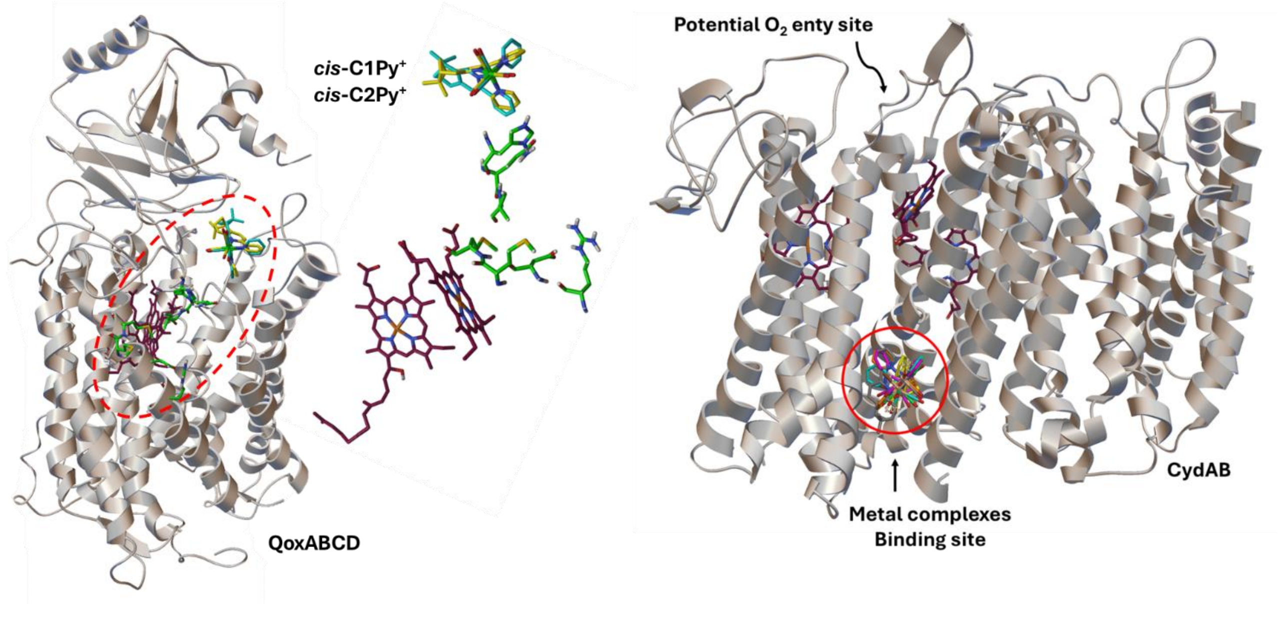

Figure S24. Left: lowest binding poses of *cis*-[C1Py]$^+$ and *cis*-[C1Py]$^+$ with *qoxABCD*. Only subunits I and II of *qoxABCD* are shown. Right: binding poses of complexes in *cydAB*. Note how all complexes bind in the same pocket.

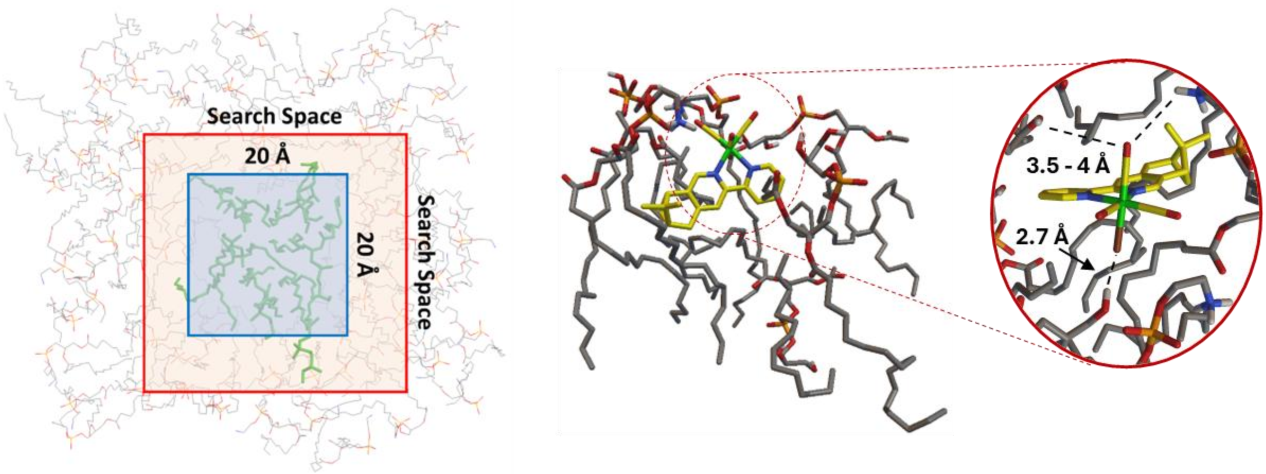

Figure S25. Left: top view of the search space and fully flexible 20x20 Å area (green lipids) defined in the conformational analysis in the semi-flexible POPG:POPE membrane model. Right: view of the lowest-energy insertion conformation of **C2Br** in the semi-flexible POPG:POPE membrane model showing weak, electrostatic H-bonding interaction the lipids' amino and glycero head groups and the compound Br and CO ligands contributing to the conformation's stabilization.
