# Supplementary material for: Pinene-Based Chiral Bipyridine Ligands Drive Potent Antibacterial Activity in Rhenium(I) Complexes
Source: Molecules. 2025 Jul 29;30(15):3183. doi: 10.3390/molecules30153183 (PMC12348105; doi:10.3390/molecules30153183)

checkCIF/PLATON report

Structure factors have been supplied for datablock(s) jh-009

THIS REPORT IS FOR GUIDANCE ONLY. IF USED AS PART OF A REVIEW PROCEDURE FOR PUBLICATION, IT SHOULD NOT REPLACE THE EXPERTISE OF AN EXPERIENCED CRYSTALLOGRAPHIC REFEREE.

No syntax errors found.   CIF dictionary   Interpreting this report

Datablock: jh-009

Bond precision: C-C = 0.0094 A   Wavelength=1.54186

Cell:	a=13.0509(3)	b=11.1063(2)	c=14.5477(3)
	alpha=90	beta=111.263(1)	gamma=90
Temperature:	250 K		

	Calculated	Reported
Volume	1965.10(7)	1965.10(7)
Space group	P 21	P 1 21 1
Hall group	P 2yb	P 2yb
Moiety formula	C20 H18 Br N2 O3 Re	2(C20 H18 Br N2 O3 Re)
Sum formula	C20 H18 Br N2 O3 Re	C40 H36 Br2 N4 O6 Re2
Mr	600.47	1200.95
Dx,g cm-3	2.030	2.030
Z	4	2
Mu (mm-1)	14.670	14.670
F000	1144.0	1144.0
F000'	1122.81	
h,k,lmax	16,14,18	16,12,15
Nref	8927[4696]	5132
Tmin,Tmax	0.074,0.644	0.026,0.327
Tmin'	0.000	

Correction method= # Reported T Limits: Tmin=0.026 Tmax=0.327
AbsCorr = MULTI-SCAN

Data completeness= 1.09/0.57   Theta(max)= 86.096

R(reflections)= 0.0203(5061)   wR2(reflections)= 0.0506(5132)

S = 1.080   Npar= 491

The following ALERTS were generated. Each ALERT has the format
test-name_ALERT_alert-type_alert-level.
Click on the hyperlinks for more details of the test.

Alert level A

```
PLAT029_ALERT_3_A _diffrn_measured_fraction_theta_full value Low .      0.916 Why?
```

Author Response: Low diffraction intensities in high angles

Alert level B

```
PLAT915_ALERT_3_B No Flack x Check Done: Low Friedel Pair Coverage         35 %
```

Alert level C

```
PLAT090_ALERT_3_C Poor Data / Parameter Ratio (Zmax > 18) ........       7.46 Note
PLAT250_ALERT_2_C Large U3/U1 Ratio for Average U(i,j) Tensor ....        2.1 Note
PLAT250_ALERT_2_C Large U3/U1 Ratio for Average U(i,j) Tensor ....        2.4 Note
PLAT342_ALERT_3_C Low Bond Precision on  C-C Bonds ...............    0.00944 Ang.
PLAT361_ALERT_2_C Long   C(sp3)-C(sp3) Bond  C12      - C15      .       1.68 Ang.
PLAT361_ALERT_2_C Long   C(sp3)-C(sp3) Bond  C13      - C14      .       1.68 Ang.
PLAT361_ALERT_2_C Long   C(sp3)-C(sp3) Bond  C15      - C16      .       1.68 Ang.
PLAT361_ALERT_2_C Long   C(sp3)-C(sp3) Bond  C32      - C33      .       1.65 Ang.
PLAT361_ALERT_2_C Long   C(sp3)-C(sp3) Bond  C35      - C36      .       1.67 Ang.
PLAT362_ALERT_2_C Short  C(sp3)-C(sp2) Bond  C10      - C11      .       1.39 Ang.
PLAT362_ALERT_2_C Short  C(sp3)-C(sp2) Bond  C30      - C31      .       1.36 Ang.
PLAT911_ALERT_3_C Missing FCF Refl Between Thmin & STh/L=    0.600        224 Report
PLAT974_ALERT_2_C Check Calcd Negative Resid. Density on       Re2      -1.08 eA-3
PLAT974_ALERT_2_C Check Calcd Negative Resid. Density on       Re1      -1.03 eA-3
```

Alert level G

```
PLAT042_ALERT_1_G Calc. and Reported Moiety Formula Strings Differ     Please Check
PLAT045_ALERT_1_G Calculated and Reported Z Differ by a Factor ...       2.00 Check
PLAT111_ALERT_2_G ADDSYM Detects New (Pseudo) Centre of Symmetry .         81 %Fit
PLAT113_ALERT_2_G ADDSYM Suggests Possible Pseudo/New  Space Group      P21/c Check
PLAT232_ALERT_2_G Hirshfeld Test Diff (M-X)  Re2      --C39      .        5.5 s.u.
PLAT232_ALERT_2_G Hirshfeld Test Diff (M-X)  Re2      --C40      .        5.3 s.u.
PLAT343_ALERT_2_G Unusual sp3      Angle Range in Main Residue for        C35 Check
PLAT791_ALERT_4_G Model has Chirality at C12         (Sohnke SpGr)          R Verify
PLAT791_ALERT_4_G Model has Chirality at C14         (Sohnke SpGr)          R Verify
PLAT791_ALERT_4_G Model has Chirality at C32         (Sohnke SpGr)          R Verify
PLAT791_ALERT_4_G Model has Chirality at C34         (Sohnke SpGr)          R Verify
PLAT912_ALERT_4_G Missing # of FCF Reflections Above STh/L=  0.600        483 Note
PLAT933_ALERT_2_G Number of OMIT Records in Embedded .res File ...          6 Note
PLAT951_ALERT_5_G Calculated (ThMax) and CIF-Reported Kmax Differ           2 Units
PLAT952_ALERT_5_G Calculated (ThMax) and CIF-Reported Lmax Differ           3 Units
PLAT957_ALERT_1_G Calculated (ThMax) and Actual (FCF) Kmax Differ           2 Units
PLAT958_ALERT_1_G Calculated (ThMax) and Actual (FCF) Lmax Differ           3 Units
PLAT978_ALERT_2_G Number C-C Bonds with Positive Residual Density.          0 Info
```

```
 1 ALERT level A = Most likely a serious problem - resolve or explain
 1 ALERT level B = A potentially serious problem, consider carefully
14 ALERT level C = Check. Ensure it is not caused by an omission or oversight
18 ALERT level G = General information/check it is not something unexpected

 4 ALERT type 1 CIF construction/syntax error, inconsistent or missing data
18 ALERT type 2 Indicator that the structure model may be wrong or deficient
 5 ALERT type 3 Indicator that the structure quality may be low
 5 ALERT type 4 Improvement, methodology, query or suggestion
 2 ALERT type 5 Informative message, check
```

It is advisable to attempt to resolve as many as possible of the alerts in all categories. Often the minor alerts point to easily fixed oversights, errors and omissions in your CIF or refinement strategy, so attention to these fine details can be worthwhile. In order to resolve some of the more serious problems it may be necessary to carry out additional measurements or structure refinements. However, the purpose of your study may justify the reported deviations and the more serious of these should normally be commented upon in the discussion or experimental section of a paper or in the "special_details" fields of the CIF. checkCIF was carefully designed to identify outliers and unusual parameters, but every test has its limitations and alerts that are not important in a particular case may appear. Conversely, the absence of alerts does not guarantee there are no aspects of the results needing attention. It is up to the individual to critically assess their own results and, if necessary, seek expert advice.

Publication of your CIF in IUCr journals

A basic structural check has been run on your CIF. These basic checks will be run on all CIFs submitted for publication in IUCr journals (*Acta Crystallographica*, *Journal of Applied Crystallography*, *Journal of Synchrotron Radiation*); however, if you intend to submit to *Acta Crystallographica Section C* or *E* or *IUCrData*, you should make sure that full publication checks are run on the final version of your CIF prior to submission.

Publication of your CIF in other journals

Please refer to the *Notes for Authors* of the relevant journal for any special instructions relating to CIF submission.

PLATON version of 13/07/2021; check.def file version of 13/07/2021

Datablock jh-009 - ellipsoid plot

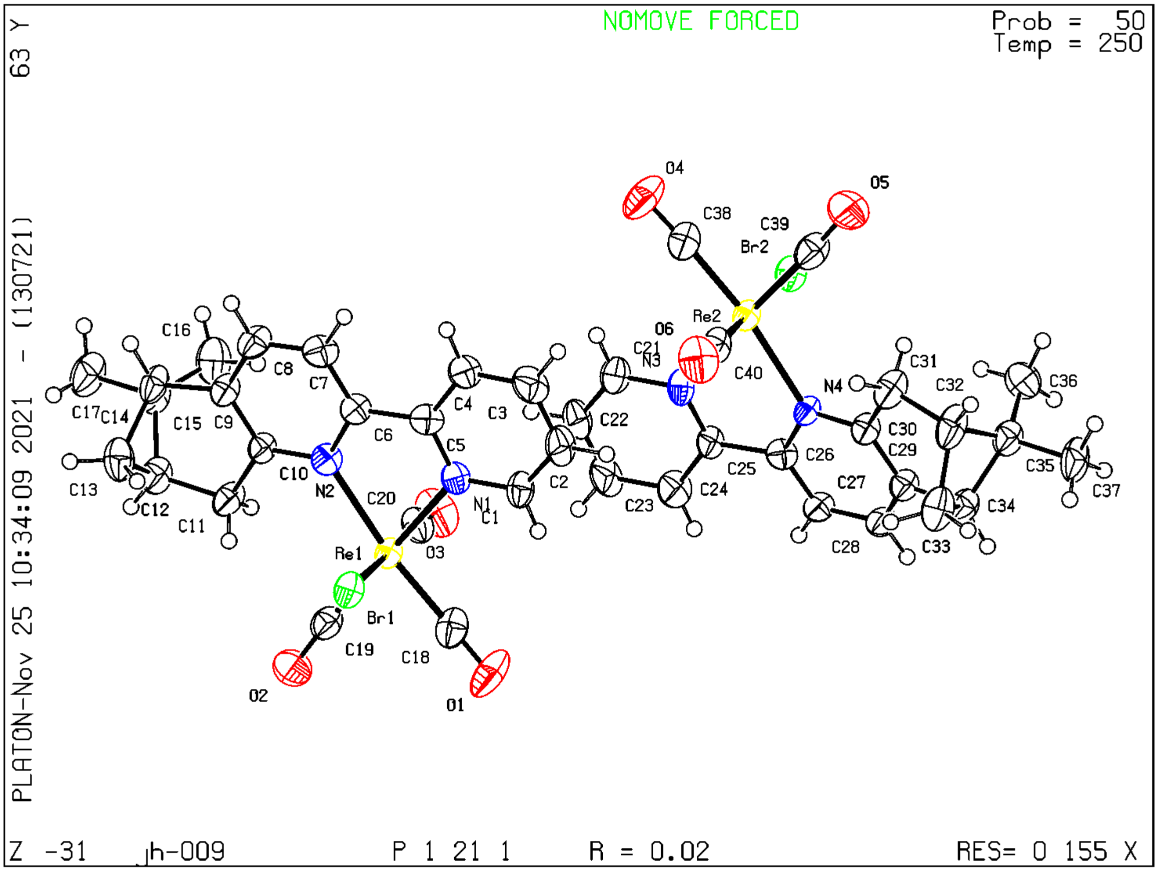

checkCIF/PLATON report

Structure factors have been supplied for datablock(s) jh-010

THIS REPORT IS FOR GUIDANCE ONLY. IF USED AS PART OF A REVIEW PROCEDURE FOR PUBLICATION, IT SHOULD NOT REPLACE THE EXPERTISE OF AN EXPERIENCED CRYSTALLOGRAPHIC REFEREE.

No syntax errors found. CIF dictionary Interpreting this report

Datablock: jh-010

Bond precision: C-C = 0.0199 A Wavelength=1.54186

Cell:	a=6.8637(2)	b=15.8821(4)	c=11.3929(3)
	alpha=90	beta=96.524(2)	gamma=90
Temperature:	250 K		

	Calculated	Reported
Volume	1233.90(6)	1233.90(6)
Space group	P 21	P 1 21 1
Hall group	P 2yb	P 2yb
Moiety formula	C20 H18 Br N2 O3 Re, C H Cl3	C20 H18 Br N2 O3 Re, C H Cl3
Sum formula	C21 H19 Br Cl3 N2 O3 Re	C21 H19 Br Cl3 N2 O3 Re
Mr	719.84	719.84
Dx,g cm-3	1.938	1.937
Z	2	2
Mu (mm-1)	14.730	14.730
F000	688.0	688.0
F000’	679.70	
h,k,lmax	8,19,13	8,18,13
Nref	4458[2317]	3344
Tmin,Tmax	0.030,0.053	0.036,0.280
Tmin’	0.005	

Correction method= # Reported T Limits: Tmin=0.036 Tmax=0.280
AbsCorr = MULTI-SCAN

Data completeness= 1.44/0.75 Theta(max)= 67.553

R(reflections)= 0.0439(3333) wR2(reflections)= 0.1181(3344)

S = 1.116 Npar= 283

The following ALERTS were generated. Each ALERT has the format
test-name_ALERT_alert-type_alert-level.
Click on the hyperlinks for more details of the test.

Alert level B

```
PLAT915_ALERT_3_B No Flack x Check Done: Low Friedel Pair Coverage         49 %
```

Alert level C

```
PLAT234_ALERT_4_C Large Hirshfeld Difference Re1      --C20      .       0.17 Ang.
PLAT241_ALERT_2_C High   'MainMol' Ueq as Compared to Neighbors of        C13 Check
PLAT244_ALERT_4_C Low    'Solvent' Ueq as Compared to Neighbors of        C21 Check
PLAT260_ALERT_2_C Large Average Ueq of Residue Including       Cl1      0.140 Check
PLAT342_ALERT_3_C Low Bond Precision on  C-C Bonds ...............    0.01994 Ang.
PLAT911_ALERT_3_C Missing FCF Refl Between Thmin & STh/L=    0.599         24 Report
PLAT971_ALERT_2_C Check Calcd Resid. Dens.  0.84A   From Re1           1.90 eA-3
PLAT972_ALERT_2_C Check Calcd Resid. Dens.  1.01A   From Re1          -1.70 eA-3
```

Alert level G

```
PLAT033_ALERT_4_G Flack x Value Deviates > 3.0 * sigma from Zero .      0.080 Note
PLAT791_ALERT_4_G Model has Chirality at C12         (Sohnke SpGr)          R Verify
PLAT791_ALERT_4_G Model has Chirality at C14         (Sohnke SpGr)          R Verify
PLAT909_ALERT_3_G Percentage of I>2sig(I) Data at Theta(Max) Still       100% Note
PLAT910_ALERT_3_G Missing # of FCF Reflection(s) Below Theta(Min).          2 Note
PLAT933_ALERT_2_G Number of OMIT Records in Embedded .res File ...          5 Note
PLAT961_ALERT_5_G Dataset Contains no Negative Intensities .......     Please Check
PLAT978_ALERT_2_G Number C-C Bonds with Positive Residual Density.          0 Info
```

0 **ALERT level A** = Most likely a serious problem - resolve or explain
1 **ALERT level B** = A potentially serious problem, consider carefully
8 **ALERT level C** = Check. Ensure it is not caused by an omission or oversight
8 **ALERT level G** = General information/check it is not something unexpected

0 ALERT type 1 CIF construction/syntax error, inconsistent or missing data
6 ALERT type 2 Indicator that the structure model may be wrong or deficient
5 ALERT type 3 Indicator that the structure quality may be low
5 ALERT type 4 Improvement, methodology, query or suggestion
1 ALERT type 5 Informative message, check

It is advisable to attempt to resolve as many as possible of the alerts in all categories. Often the minor alerts point to easily fixed oversights, errors and omissions in your CIF or refinement strategy, so attention to these fine details can be worthwhile. In order to resolve some of the more serious problems it may be necessary to carry out additional measurements or structure refinements. However, the purpose of your study may justify the reported deviations and the more serious of these should normally be commented upon in the discussion or experimental section of a paper or in the "special_details" fields of the CIF. checkCIF was carefully designed to identify outliers and unusual parameters, but every test has its limitations and alerts that are not important in a particular case may appear. Conversely, the absence of alerts does not guarantee there are no aspects of the results needing attention. It is up to the individual to critically assess their own results and, if necessary, seek expert advice.

Publication of your CIF in IUCr journals

A basic structural check has been run on your CIF. These basic checks will be run on all CIFs submitted for publication in IUCr journals (*Acta Crystallographica*, *Journal of Applied Crystallography*, *Journal of Synchrotron Radiation*); however, if you intend to submit to *Acta Crystallographica Section C* or *E* or *IUCrData*, you should make sure that full publication checks are run on the final version of your CIF prior to submission.

Publication of your CIF in other journals

Please refer to the *Notes for Authors* of the relevant journal for any special instructions relating to CIF submission.

PLATON version of 13/07/2021; check.def file version of 13/07/2021

Datablock jh-010 - ellipsoid plot

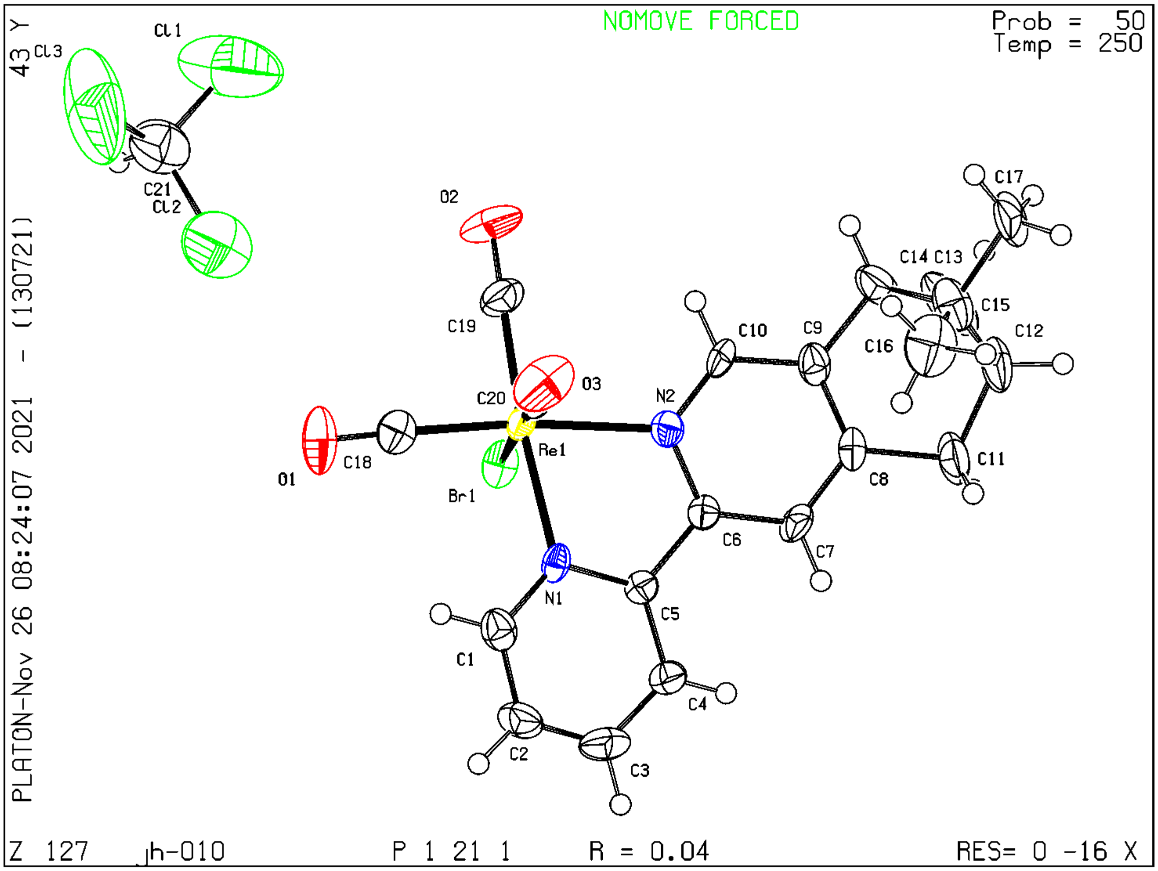

checkCIF/PLATON report

Structure factors have been supplied for datablock(s) jh-012

THIS REPORT IS FOR GUIDANCE ONLY. IF USED AS PART OF A REVIEW PROCEDURE FOR PUBLICATION, IT SHOULD NOT REPLACE THE EXPERTISE OF AN EXPERIENCED CRYSTALLOGRAPHIC REFEREE.

No syntax errors found.   CIF dictionary   Interpreting this report

Datablock: jh-012

```
Bond precision:   C-C = 0.0086 A          Wavelength=1.54186

Cell:             a=12.9643(3)     b=11.0520(2)        c=14.4107(3)
                  alpha=90         beta=110.669(2)     gamma=90
Temperature:      250 K

                 Calculated                      Reported
Volume           1931.89(7)                      1931.89(7)
Space group      P 21                            P 1 21 1
Hall group       P 2yb                           P 2yb
Moiety formula   C20 H18 Cl N2 O3 Re             C20 H18 Cl N2 O3 Re
Sum formula      C20 H18 Cl N2 O3 Re             C20 H18 Cl N2 O3 Re
Mr               556.02                          556.01
Dx,g cm-3        1.912                           1.912
Z                4                               4
Mu (mm-1)        13.771                          13.771
F000             1072.0                          1072.0
F000'            1054.96
h,k,lmax         16,14,18                        16,12,15
Nref             8811[ 4636]                     5408
Tmin,Tmax        0.040,0.662                     0.009,0.229
Tmin'            0.000

Correction method= # Reported T Limits: Tmin=0.009 Tmax=0.229
AbsCorr = MULTI-SCAN

Data completeness= 1.17/0.61       Theta(max)= 88.106

R(reflections)= 0.0173( 5375)                          wR2(reflections)=
                                                       0.0451( 5408)
S = 1.093                    Npar= 492
```

The following ALERTS were generated. Each ALERT has the format
test-name_ALERT_alert-type_alert-level.
Click on the hyperlinks for more details of the test.

Alert level A

```
PLAT029_ALERT_3_A _diffrn_measured_fraction_theta_full value Low .      0.927 Why?
```

Author Response: Low diffraction intensities in high angles

Alert level B

```
PLAT915_ALERT_3_B No Flack x Check Done: Low Friedel Pair Coverage         42 %
```

Alert level C

```
PLAT090_ALERT_3_C Poor Data / Parameter Ratio (Zmax > 18) ........       7.41 Note
PLAT250_ALERT_2_C Large U3/U1 Ratio for Average U(i,j) Tensor ....        2.3 Note
PLAT250_ALERT_2_C Large U3/U1 Ratio for Average U(i,j) Tensor ....        2.1 Note
PLAT342_ALERT_3_C Low Bond Precision on  C-C Bonds ...............    0.00861 Ang.
PLAT361_ALERT_2_C Long   C(sp3)-C(sp3) Bond  C15      - C16      .       1.67 Ang.
PLAT361_ALERT_2_C Long   C(sp3)-C(sp3) Bond  C32      - C35      .       1.67 Ang.
PLAT361_ALERT_2_C Long   C(sp3)-C(sp3) Bond  C33      - C34      .       1.68 Ang.
PLAT361_ALERT_2_C Long   C(sp3)-C(sp3) Bond  C35      - C36      .       1.66 Ang.
PLAT362_ALERT_2_C Short  C(sp3)-C(sp2) Bond  C10      - C11      .       1.36 Ang.
PLAT362_ALERT_2_C Short  C(sp3)-C(sp2) Bond  C30      - C31      .       1.40 Ang.
PLAT911_ALERT_3_C Missing FCF Refl Between Thmin & STh/L=    0.600        191 Report
```

Alert level G

```
PLAT063_ALERT_4_G Crystal Size Possibly too Large for Beam Size ..       0.67 mm
PLAT111_ALERT_2_G ADDSYM Detects New (Pseudo) Centre of Symmetry .         81 %Fit
PLAT113_ALERT_2_G ADDSYM Suggests Possible Pseudo/New  Space Group      P21/c Check
PLAT343_ALERT_2_G Unusual sp3      Angle Range in Main Residue for        C15 Check
PLAT343_ALERT_2_G Unusual sp3      Angle Range in Main Residue for        C35 Check
PLAT432_ALERT_2_G Short Inter X...Y Contact  O4       ..C24              3.00 Ang.
                                           1-x,1/2+y,1-z  =      2_656 Check
PLAT791_ALERT_4_G Model has Chirality at C12         (Sohnke SpGr)          R Verify
PLAT791_ALERT_4_G Model has Chirality at C14         (Sohnke SpGr)          R Verify
PLAT791_ALERT_4_G Model has Chirality at C32         (Sohnke SpGr)          R Verify
PLAT791_ALERT_4_G Model has Chirality at C34         (Sohnke SpGr)          R Verify
PLAT910_ALERT_3_G Missing # of FCF Reflection(s) Below Theta(Min).          1 Note
PLAT912_ALERT_4_G Missing # of FCF Reflections Above STh/L=  0.600        501 Note
PLAT933_ALERT_2_G Number of OMIT Records in Embedded .res File ...          1 Note
PLAT951_ALERT_5_G Calculated (ThMax) and CIF-Reported Kmax Differ           2 Units
PLAT952_ALERT_5_G Calculated (ThMax) and CIF-Reported Lmax Differ           3 Units
PLAT957_ALERT_1_G Calculated (ThMax) and Actual (FCF) Kmax Differ           2 Units
PLAT958_ALERT_1_G Calculated (ThMax) and Actual (FCF) Lmax Differ           3 Units
PLAT961_ALERT_5_G Dataset Contains no Negative Intensities .......     Please Check
PLAT978_ALERT_2_G Number C-C Bonds with Positive Residual Density.          0 Info
```

1 **ALERT level A** = Most likely a serious problem - resolve or explain

```
 1 ALERT level B = A potentially serious problem, consider carefully
11 ALERT level C = Check. Ensure it is not caused by an omission or oversight
19 ALERT level G = General information/check it is not something unexpected

 2 ALERT type 1 CIF construction/syntax error, inconsistent or missing data
15 ALERT type 2 Indicator that the structure model may be wrong or deficient
 6 ALERT type 3 Indicator that the structure quality may be low
 6 ALERT type 4 Improvement, methodology, query or suggestion
 3 ALERT type 5 Informative message, check
```

It is advisable to attempt to resolve as many as possible of the alerts in all categories. Often the minor alerts point to easily fixed oversights, errors and omissions in your CIF or refinement strategy, so attention to these fine details can be worthwhile. In order to resolve some of the more serious problems it may be necessary to carry out additional measurements or structure refinements. However, the purpose of your study may justify the reported deviations and the more serious of these should normally be commented upon in the discussion or experimental section of a paper or in the "special_details" fields of the CIF. checkCIF was carefully designed to identify outliers and unusual parameters, but every test has its limitations and alerts that are not important in a particular case may appear. Conversely, the absence of alerts does not guarantee there are no aspects of the results needing attention. It is up to the individual to critically assess their own results and, if necessary, seek expert advice.

Publication of your CIF in IUCr journals

A basic structural check has been run on your CIF. These basic checks will be run on all CIFs submitted for publication in IUCr journals (*Acta Crystallographica*, *Journal of Applied Crystallography*, *Journal of Synchrotron Radiation*); however, if you intend to submit to *Acta Crystallographica Section C* or *E* or *IUCrData*, you should make sure that full publication checks are run on the final version of your CIF prior to submission.

Publication of your CIF in other journals

Please refer to the *Notes for Authors* of the relevant journal for any special instructions relating to CIF submission.

PLATON version of 13/07/2021; check.def file version of 13/07/2021

Datablock jh-012 - ellipsoid plot

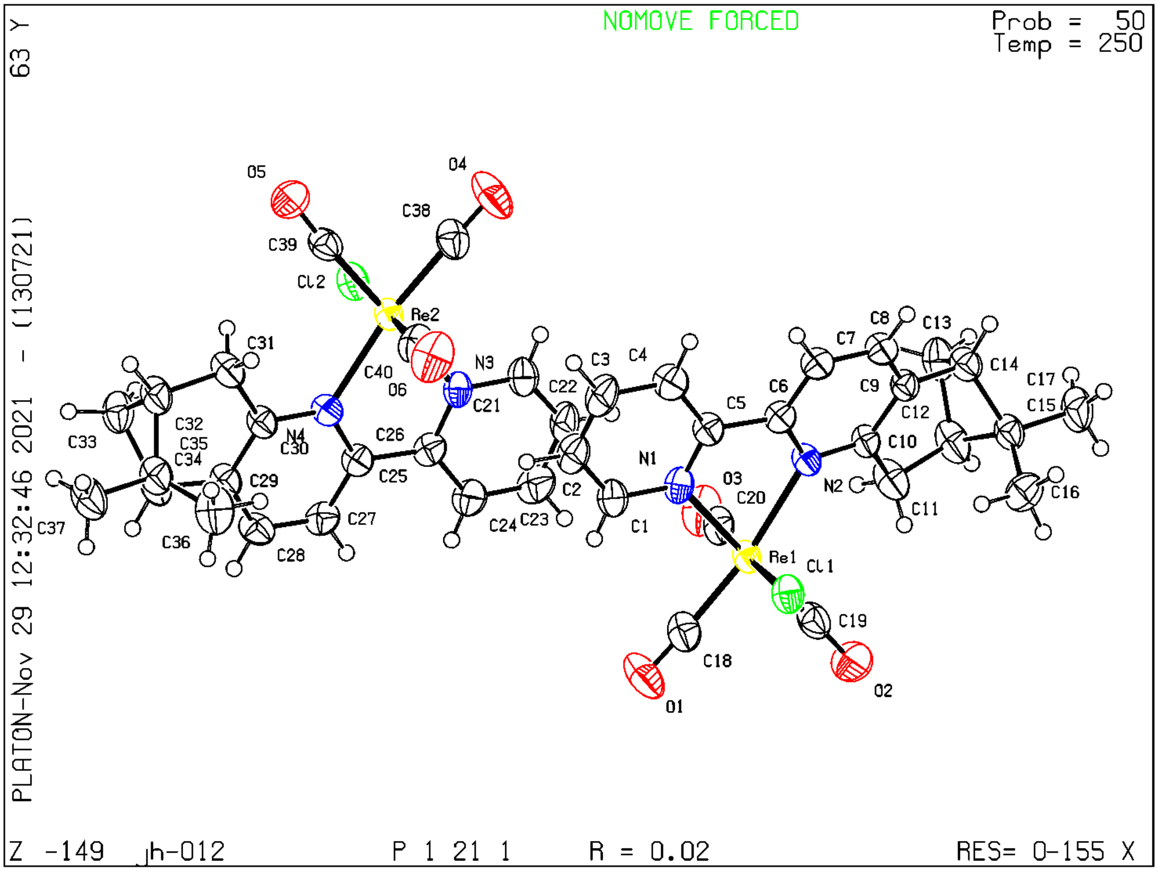

checkCIF/PLATON report

Structure factors have been supplied for datablock(s) jh-017

THIS REPORT IS FOR GUIDANCE ONLY. IF USED AS PART OF A REVIEW PROCEDURE FOR PUBLICATION, IT SHOULD NOT REPLACE THE EXPERTISE OF AN EXPERIENCED CRYSTALLOGRAPHIC REFEREE.

No syntax errors found. CIF dictionary Interpreting this report

Datablock: jh-017

Bond precision:	C-C = 0.0132 A		Wavelength=1.54186
Cell:	a=27.7001(3)	b=15.2103(2)	c=12.9173(1)
	alpha=90	beta=90	gamma=90
Temperature:	250 K		

	Calculated	Reported
Volume	5442.41(10)	5442.41(10)
Space group	P 21 21 21	P 21 21 21
Hall group	P 2ac 2ab	P 2ac 2ab
Moiety formula	C25 H23 N3 O3 Re, C F3 O3 S	2(C25 H23 N3 O3 Re), 2(C F3 O3 S)
Sum formula	C26 H23 F3 N3 O6 Re S	C52 H46 F6 N6 O12 Re2 S2
Mr	748.74	1497.47
Dx,g cm-3	1.828	1.828
Z	8	4
Mu (mm-1)	10.048	10.048
F000	2928.0	2928.0
F000’	2897.43	
h,k,lmax	33,18,15	32,17,14
Nref	9930[5504]	9422
Tmin,Tmax	0.073,0.366	0.054,0.245
Tmin’	0.001	

Correction method= # Reported T Limits: Tmin=0.054 Tmax=0.245
AbsCorr = MULTI-SCAN

Data completeness= 1.71/0.95 Theta(max)= 68.141

R(reflections)= 0.0276(9293) wR2(reflections)= 0.0747(9422)

S = 1.027 Npar= 725

The following ALERTS were generated. Each ALERT has the format

test-name_ALERT_alert-type_alert-level.

Click on the hyperlinks for more details of the test.

Alert level B

```
PLAT029_ALERT_3_B _diffrn_measured_fraction_theta_full value Low .      0.958 Why?
PLAT910_ALERT_3_B Missing # of FCF Reflection(s) Below Theta(Min).         11 Note
```

Alert level C

```
PLAT090_ALERT_3_C Poor Data / Parameter Ratio (Zmax > 18) ........       7.23 Note
PLAT234_ALERT_4_C Large Hirshfeld Difference C38      --C39      .       0.18 Ang.
PLAT234_ALERT_4_C Large Hirshfeld Difference S1       --C51      .       0.16 Ang.
PLAT242_ALERT_2_C Low    'MainMol' Ueq as Compared to Neighbors of        C40 Check
PLAT244_ALERT_4_C Low    'Solvent' Ueq as Compared to Neighbors of         S1 Check
PLAT244_ALERT_4_C Low    'Solvent' Ueq as Compared to Neighbors of         S2 Check
PLAT260_ALERT_2_C Large Average Ueq of Residue Including        S1      0.140 Check
PLAT260_ALERT_2_C Large Average Ueq of Residue Including        S2      0.133 Check
PLAT342_ALERT_3_C Low Bond Precision on  C-C Bonds ...............    0.01316 Ang.
PLAT911_ALERT_3_C Missing FCF Refl Between Thmin & STh/L=    0.600        163 Report
```

Alert level G

```
PLAT042_ALERT_1_G Calc. and Reported Moiety Formula Strings Differ     Please Check
PLAT045_ALERT_1_G Calculated and Reported Z Differ by a Factor ...          2 Check
PLAT063_ALERT_4_G Crystal Size Possibly too Large for Beam Size ..       0.65 mm
PLAT083_ALERT_2_G SHELXL Second Parameter in WGHT  Unusually Large       5.54 Why ?
PLAT143_ALERT_4_G s.u. on c - Axis Small or Missing .............    0.00010 Ang.
PLAT230_ALERT_2_G Hirshfeld Test Diff for    O2       --C24      .        8.3 s.u.
PLAT231_ALERT_4_G Hirshfeld Test (Solvent)   S1       --O8       .        6.9 s.u.
PLAT232_ALERT_2_G Hirshfeld Test Diff (M-X)  Re1      --C24      .        8.2 s.u.
PLAT232_ALERT_2_G Hirshfeld Test Diff (M-X)  Re2      --C49      .        5.2 s.u.
PLAT244_ALERT_4_G Low    'Solvent' Ueq as Compared to Neighbors of        C51 Check
PLAT244_ALERT_4_G Low    'Solvent' Ueq as Compared to Neighbors of        C52 Check
PLAT790_ALERT_4_G Centre of Gravity not Within Unit Cell: Resd.  #          2 Note
              C25 H23 N3 O3 Re
PLAT791_ALERT_4_G Model has Chirality at C12         (Sohnke SpGr)          R Verify
PLAT791_ALERT_4_G Model has Chirality at C14         (Sohnke SpGr)          R Verify
PLAT791_ALERT_4_G Model has Chirality at C37         (Sohnke SpGr)          R Verify
PLAT791_ALERT_4_G Model has Chirality at C39         (Sohnke SpGr)          R Verify
PLAT909_ALERT_3_G Percentage of I>2sig(I) Data at Theta(Max) Still        96% Note
PLAT912_ALERT_4_G Missing # of FCF Reflections Above STh/L=  0.600         29 Note
PLAT978_ALERT_2_G Number C-C Bonds with Positive Residual Density.          0 Info
```

0 **ALERT level A** = Most likely a serious problem - resolve or explain
2 **ALERT level B** = A potentially serious problem, consider carefully
10 **ALERT level C** = Check. Ensure it is not caused by an omission or oversight
19 **ALERT level G** = General information/check it is not something unexpected

2 ALERT type 1 CIF construction/syntax error, inconsistent or missing data

```
  8 ALERT type 2 Indicator that the structure model may be wrong or deficient
  6 ALERT type 3 Indicator that the structure quality may be low
 15 ALERT type 4 Improvement, methodology, query or suggestion
  0 ALERT type 5 Informative message, check
```

It is advisable to attempt to resolve as many as possible of the alerts in all categories. Often the minor alerts point to easily fixed oversights, errors and omissions in your CIF or refinement strategy, so attention to these fine details can be worthwhile. In order to resolve some of the more serious problems it may be necessary to carry out additional measurements or structure refinements. However, the purpose of your study may justify the reported deviations and the more serious of these should normally be commented upon in the discussion or experimental section of a paper or in the "special_details" fields of the CIF. checkCIF was carefully designed to identify outliers and unusual parameters, but every test has its limitations and alerts that are not important in a particular case may appear. Conversely, the absence of alerts does not guarantee there are no aspects of the results needing attention. It is up to the individual to critically assess their own results and, if necessary, seek expert advice.

Publication of your CIF in IUCr journals

A basic structural check has been run on your CIF. These basic checks will be run on all CIFs submitted for publication in IUCr journals (*Acta Crystallographica*, *Journal of Applied Crystallography*, *Journal of Synchrotron Radiation*); however, if you intend to submit to *Acta Crystallographica Section C* or *E* or *IUCrData*, you should make sure that full publication checks are run on the final version of your CIF prior to submission.

Publication of your CIF in other journals

Please refer to the *Notes for Authors* of the relevant journal for any special instructions relating to CIF submission.

PLATON version of 19/02/2022; check.def file version of 19/02/2022

Datablock jh-017 - ellipsoid plot

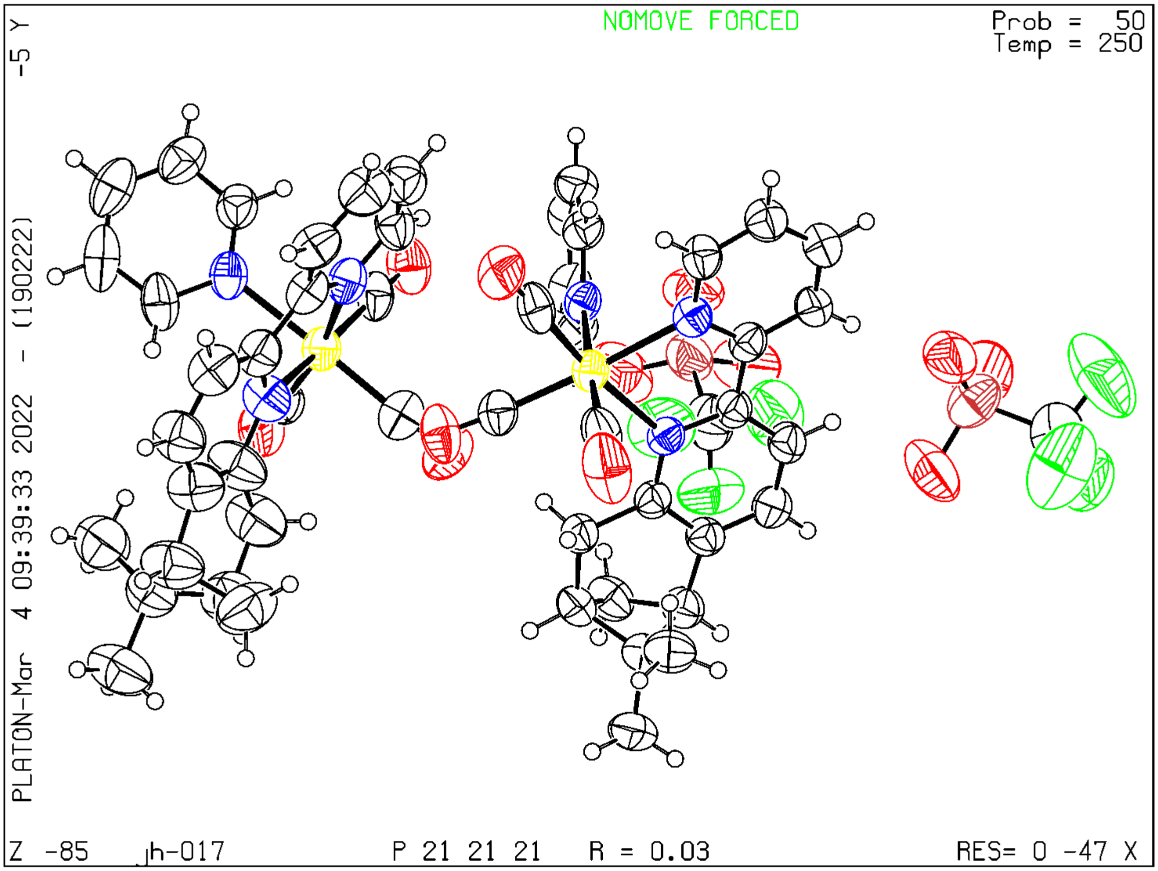

checkCIF/PLATON report

Structure factors have been supplied for datablock(s) jh-015

THIS REPORT IS FOR GUIDANCE ONLY. IF USED AS PART OF A REVIEW PROCEDURE FOR PUBLICATION, IT SHOULD NOT REPLACE THE EXPERTISE OF AN EXPERIENCED CRYSTALLOGRAPHIC REFEREE.

No syntax errors found. CIF dictionary Interpreting this report

Datablock: jh-015

Bond precision: C-C = 0.0186 A Wavelength=0.71073

Cell:	a=10.3305(9)	b=13.2858(8)	c=20.5032(19)
	alpha=90	beta=91.585(7)	gamma=90
Temperature:	250 K		

	Calculated	Reported
Volume	2813.0(4)	2813.0(4)
Space group	P 21	P 1 21 1
Hall group	P 2yb	P 2yb
Moiety formula	C25 H23 N3 O3 Re, C F3 O3 S	C25 H23 N3 O3 Re, C F3 O3 S
Sum formula	C26 H23 F3 N3 O6 Re S	C26 H23 F3 N3 O6 Re S
Mr	748.74	748.73
Dx,g cm-3	1.768	1.768
Z	4	4
Mu (mm-1)	4.458	4.458
F000	1464.0	1464.0
F000’	1461.23	
h,k,lmax	12,16,25	12,16,25
Nref	11328[5919]	10795
Tmin,Tmax	0.453,0.800	0.218,0.503
Tmin’	0.152	

Correction method= # Reported T Limits: Tmin=0.218 Tmax=0.503
AbsCorr = MULTI-SCAN

Data completeness= 1.82/0.95 Theta(max)= 26.216

R(reflections)= 0.0404(9379) wR2(reflections)= 0.0985(10795)

S = 1.068 Npar= 726

The following ALERTS were generated. Each ALERT has the format
test-name_ALERT_alert-type_alert-level.
Click on the hyperlinks for more details of the test.

Alert level B

```
PLAT430_ALERT_2_B Short Inter D...A Contact  O3       ..O6       .       2.80 Ang.
                                                  -1+x,y,z  =      1_455 Check
```

Alert level C

```
PLAT234_ALERT_4_C Large Hirshfeld Difference Re1      --C24      .       0.18 Ang.
PLAT234_ALERT_4_C Large Hirshfeld Difference O2       --C24      .       0.18 Ang.
PLAT234_ALERT_4_C Large Hirshfeld Difference O3       --C25      .       0.17 Ang.
PLAT234_ALERT_4_C Large Hirshfeld Difference C12      --C13      .       0.19 Ang.
PLAT234_ALERT_4_C Large Hirshfeld Difference Re2      --C48      .       0.18 Ang.
PLAT234_ALERT_4_C Large Hirshfeld Difference Re2      --C49      .       0.18 Ang.
PLAT234_ALERT_4_C Large Hirshfeld Difference Re2      --C50      .       0.17 Ang.
PLAT234_ALERT_4_C Large Hirshfeld Difference O5       --C49      .       0.18 Ang.
PLAT234_ALERT_4_C Large Hirshfeld Difference O6       --C50      .       0.17 Ang.
PLAT244_ALERT_4_C Low    'Solvent' Ueq as Compared to Neighbors of         S1 Check
PLAT244_ALERT_4_C Low    'Solvent' Ueq as Compared to Neighbors of         S2 Check
PLAT260_ALERT_2_C Large Average Ueq of Residue Including        S1      0.127 Check
PLAT260_ALERT_2_C Large Average Ueq of Residue Including        S2      0.126 Check
PLAT342_ALERT_3_C Low Bond Precision on  C-C Bonds ...............    0.01857 Ang.
```

Alert level G

```
PLAT111_ALERT_2_G ADDSYM Detects New (Pseudo) Centre of Symmetry .         92 %Fit
PLAT113_ALERT_2_G ADDSYM Suggests Possible Pseudo/New  Space Group      P21/n Check
PLAT244_ALERT_4_G Low    'Solvent' Ueq as Compared to Neighbors of        C51 Check
PLAT244_ALERT_4_G Low    'Solvent' Ueq as Compared to Neighbors of        C52 Check
PLAT432_ALERT_2_G Short Inter X...Y Contact  O4       ..C30      .       3.02 Ang.
                                             1-x,1/2+y,1-z  =      2_656 Check
PLAT790_ALERT_4_G Centre of Gravity not Within Unit Cell: Resd.  #          3 Note
             C F3 O3 S
PLAT791_ALERT_4_G Model has Chirality at C12         (Sohnke SpGr)          R Verify
PLAT791_ALERT_4_G Model has Chirality at C14         (Sohnke SpGr)          R Verify
PLAT791_ALERT_4_G Model has Chirality at C37         (Sohnke SpGr)          R Verify
PLAT791_ALERT_4_G Model has Chirality at C39         (Sohnke SpGr)          R Verify
PLAT910_ALERT_3_G Missing # of FCF Reflection(s) Below Theta(Min).          1 Note
PLAT912_ALERT_4_G Missing # of FCF Reflections Above STh/L=  0.600         58 Note
PLAT978_ALERT_2_G Number C-C Bonds with Positive Residual Density.          0 Info
```

0 **ALERT level A** = Most likely a serious problem - resolve or explain
1 **ALERT level B** = A potentially serious problem, consider carefully
14 **ALERT level C** = Check. Ensure it is not caused by an omission or oversight
13 **ALERT level G** = General information/check it is not something unexpected

0 ALERT type 1 CIF construction/syntax error, inconsistent or missing data
7 ALERT type 2 Indicator that the structure model may be wrong or deficient
2 ALERT type 3 Indicator that the structure quality may be low
19 ALERT type 4 Improvement, methodology, query or suggestion
0 ALERT type 5 Informative message, check

It is advisable to attempt to resolve as many as possible of the alerts in all categories. Often the minor alerts point to easily fixed oversights, errors and omissions in your CIF or refinement strategy, so attention to these fine details can be worthwhile. In order to resolve some of the more serious problems it may be necessary to carry out additional measurements or structure refinements. However, the purpose of your study may justify the reported deviations and the more serious of these should normally be commented upon in the discussion or experimental section of a paper or in the "special_details" fields of the CIF. checkCIF was carefully designed to identify outliers and unusual parameters, but every test has its limitations and alerts that are not important in a particular case may appear. Conversely, the absence of alerts does not guarantee there are no aspects of the results needing attention. It is up to the individual to critically assess their own results and, if necessary, seek expert advice.

Publication of your CIF in IUCr journals

A basic structural check has been run on your CIF. These basic checks will be run on all CIFs submitted for publication in IUCr journals (*Acta Crystallographica*, *Journal of Applied Crystallography*, *Journal of Synchrotron Radiation*); however, if you intend to submit to *Acta Crystallographica Section C* or *E* or *IUCrData*, you should make sure that full publication checks are run on the final version of your CIF prior to submission.

Publication of your CIF in other journals

Please refer to the *Notes for Authors* of the relevant journal for any special instructions relating to CIF submission.

PLATON version of 18/05/2022; check.def file version of 17/05/2022

Datablock jh-015 - ellipsoid plot

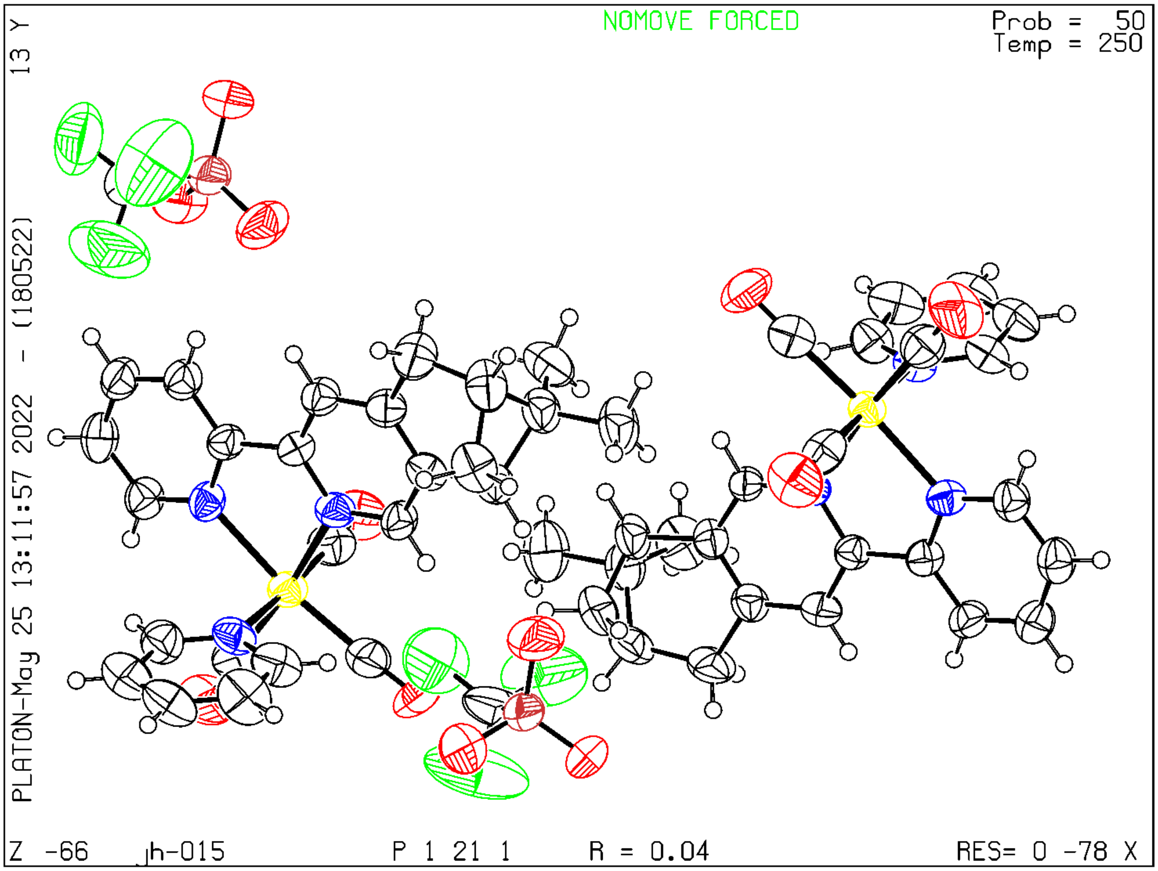

Supplement: Supplementary file 1 [file molecules-30-03183-s001.zip › checkCIF.pdf]
